# Supplementary material for: ECM degradation in the Drosophila abdominal epidermis initiates tissue growth that ceases with rapid cell-cycle exit
Source: Curr Biol. 2022 Mar 28;32(6):1285–1300.e4. doi: 10.1016/j.cub.2022.01.045 (PMC8967408; doi:10.1016/j.cub.2022.01.045)
Supplement: Document S1. Figures S1–S7 [file mmc1.pdf]

Current Biology, Volume *32*

Supplemental Information

ECM degradation in the *Drosophila* abdominal epidermis initiates tissue growth that ceases with rapid cell-cycle exit

John Robert Davis, Anna P. Ainslie, John J. Williamson, Ana Ferreira, Alejandro Torres-Sánchez, Andreas Hoppe, Federica Mangione, Matthew B. Smith, Enrique Martin-Blanco, Guillaume Salbreux, and Nicolas Tapon

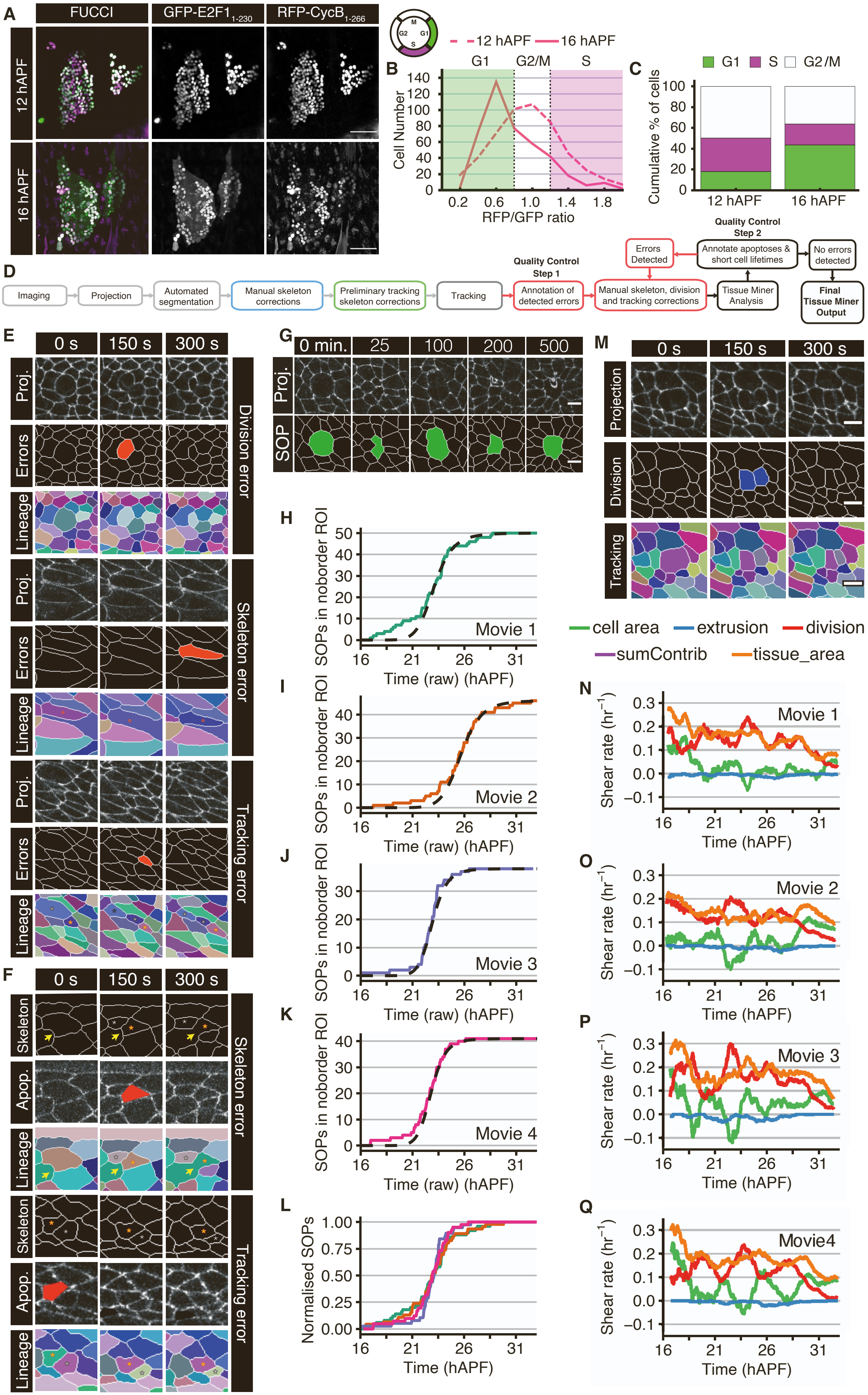

A
FUCCI
GFP-E2F1 1-230
RFP-CycB 1-266
12 hAPF
16 hAPF
B
12 hAPF
16 hAPF
G1
G2/M
S
Cell Number
RFP/GFP ratio
C
G1
S
G2/M
Cumulative % of cells
D
Imaging
Projection
Automated segmentation
Manual skeleton corrections
Preliminary tracking skeleton corrections
Tracking
Quality Control Step 1
Annotation of detected errors
Manual skeleton, division and tracking corrections
Tissue Miner Analysis
Quality Control Step 2
Annotate apoptoses & short cell lifetimes
Errors Detected
No errors detected
Final Tissue Miner Output
E
0 s
150 s
300 s
Proj.
Errors
Lineage
Division error
Skeleton error
Tracking error
F
Skeleton
Apop.
G
0 min.
25
100
200
500
SOP
H
SOPs in noborder ROI
Time (raw) (hAPF)
Movie 1
I
Movie 2
J
Movie 3
K
Movie 4
L
Normalised SOPs
Time (hAPF)
M
Projection
Division
Tracking
cell area
extrusion
division
sumContrib
tissue_area
N
Shear rate (hr^{-1})
O
P
Q
Movie4

Figure S1. Segmentation, tracking and temporal alignment of histoblast movies, Related to Figure 1, Video S1, S2, and Method S1.

(A) Example confocal images of wild-type and pupa expressing the FUCCI cell-cycle reporter markers *GFP-E2F1* (middle) and *RFP-CycB* (right) at the timepoints indicated. Schematic on far-right shows expected combined color for cells at the different stages of the cell cycle. Scale bars = 50μm.

(B) Quantification of normalized RFP/GFP signal ratio per cell at 12 hAPF and 16 hAPF pupae. N = 2 at each timepoint.

(C) Quantification of the percentage of cells at each cell-cycle phase based on normalized RFP/GFP signal ratio with the following thresholds G1 < 0.8 < G2/M < 1.4 < S-phase and highlighted in **B**.

(D) Overview of the pipeline including imaging, skeletonization, correction steps, tracking, quality control steps, and final output for analysis (see **STAR Methods** for details).

(E) Examples of error types detected by Tracker. Top, stills from a movie of a live pupal abdomen expressing *E-cad::GFP* after undergoing contour projection. Middle, error output generated by Tracker, with errors labelled in red. Bottom, lineage output from Tracker with each cell labelled with a unique color. Red and black stars indicate the location of the same cell and daughter cells of the same lineage between frames. Division error: red cell occurs because division has not been accurately detected by Tracker, thus the new cell is detected as an error. Skeleton Error: red cell occurs because of missing junction. When junction returns at 300 s, the cell has a new I.D., which is labelled as an error. Tracking error: red cell occurs due to rapid cell migration and cell division. New daughter cell from division is mistakenly labelled as a pre-existing cell at 150 s (black star, dark purple cell), and pre-existing cell is mistakenly labelled with a new cell I.D. at 150 s (red star, pink cell), which is labelled as an error.

(F) Examples of supposed cell losses (loss of cell I.D) detected by Tissue Miner which are skeleton and tracking errors. Top, skeleton analyzed by Tissue Miner. Middle, stills from a movie of a pupa expressing *E-cad::GFP* after undergoing contour projection, with the lost cell labelled in red by Tissue Miner. Bottom, lineage output from Tracker with each cell labelled with a unique color. Yellow arrows point towards the missing junction. Red and black stars indicate the same cell between frames. Skeleton Error: Missing junction labelled with yellow arrow at 300 s means that the final frame for the brown cell is at 150 s, thus it is labelled as a cell loss event at this time. Tracking Error: Cells are migrating rapidly and Tracker mislabels the green cell as the purple cell at 150 s. The green cell is labelled as an imminent apoptosis at the final frame where it is visible at 0 s.

(G) Top: Stills from a movie of a pupa expressing *E-cad::GFP*, following a sensory organ precursor cell (SOP) undergoing differentiation. Bottom: Skeletonization of SOP as a single cell (green-filled) from the moment of first asymmetric division to simplify the segmentation and tracking process. Scale bar = 5 *μ*m.

(H-L) Time-alignment method for multiple wild-type movies. **(L)** Normalized count of the appearance of sensory organ precursors (SOPs) in the noborder ROI over time. Each movie is time-aligned relative to movie 1 (whose first frame is set to be at 16 hAPF), by an offset determined by fitting Hill functions to the individual movie data shown in **(H-K)**.

(M) Tracking and cell-division masks for input into the Tissue Miner software. Top row: example movie frames showing a cell division. Middle row: cell division mask of the same frames. In the first frame after the division, the newly-produced daughter cells are marked blue. Bottom row: tracking mask, in which each cell is marked with a unique color that persists over time. To match the convention Tissue Miner expects, one daughter cell retains the color of the mother, but will be parsed as a new cell by Tissue Miner. Scale bar = 5 μm.

(N-Q) Contributions to area expansion rate of the noborder ROI from cell area relative rate of change, cell division rate, cell extrusion rate. The sum of these 3 contributions matches the directly measured area expansion rate. Integration of the shear rate leads to the cumulative contributions, e.g. as for movie 1 in **Figure 1F**.

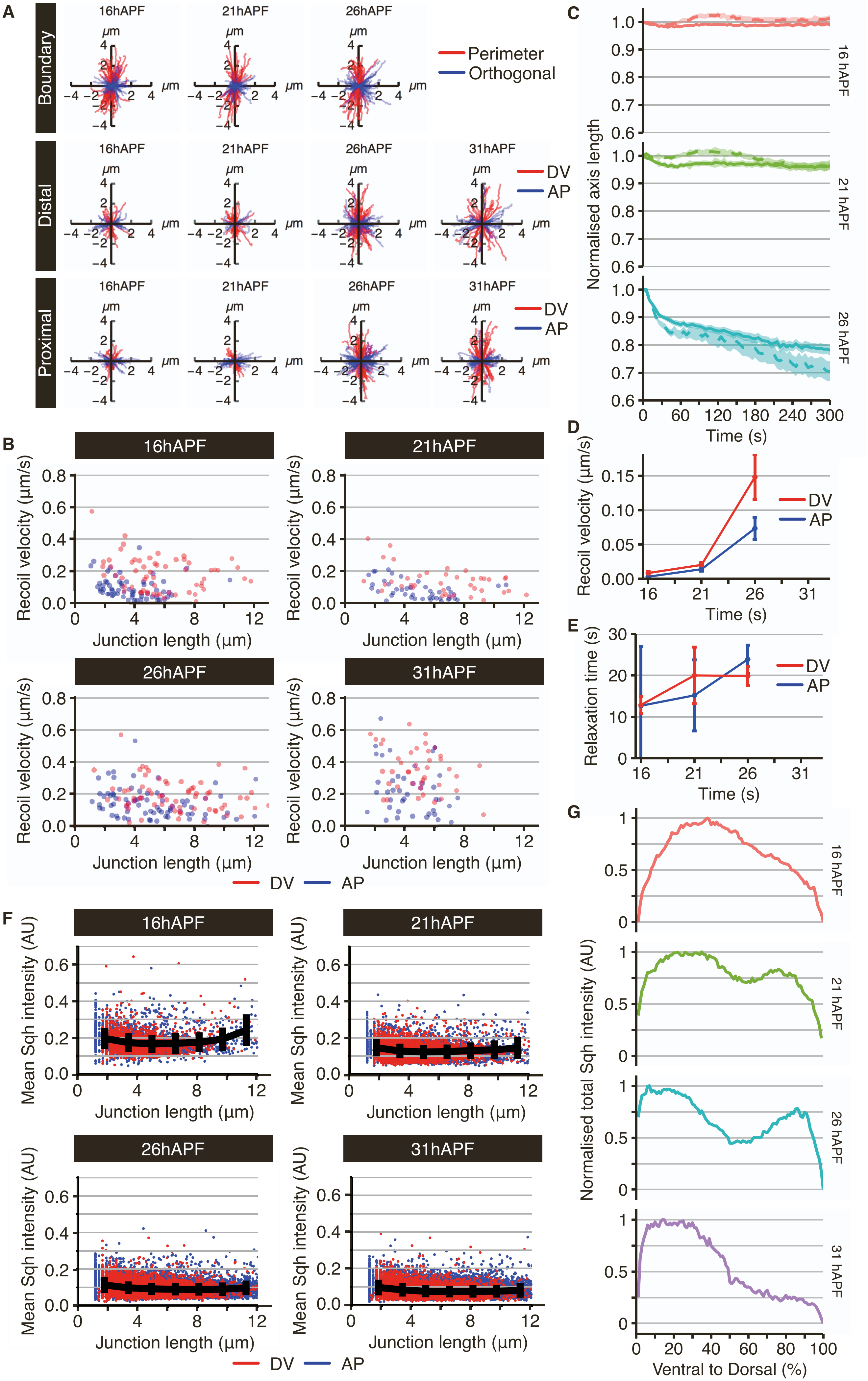

A
Boundary
Distal
Proximal
16hAPF
21hAPF
26hAPF
31hAPF
μm
Perimeter
Orthogonal
DV
AP
B
Recoil velocity (μm/s)
Junction length (μm)
C
Normalised axis length
16 hAPF
21 hAPF
26 hAPF
Time (s)
D
E
Relaxation time (s)
F
Mean Sqh intensity (AU)
G
Normalised total Sqh intensity (AU)
Ventral to Dorsal (%)

Figure S2. Histoblast junction strain increases through development and is independent of junction length or Myo II intensity, Related to Figure 2, Video S3 and S4.

(A) Trajectories of vertices after single junction ablations, relative to their initial positions, in the different populations of histoblasts (see **Figure 2B**) throughout development. Note that for boundary cells, the trajectories of vertices shared with LECs are on the left and experience reduced displacement compared to the vertex within the nest.

(B) Quantification of junction recoil velocity as a function of junction length throughout development reveals no clear correlation.

(C) Normalized length of the short (oriented along the AP axis, dashed) and long (oriented along the DV axis, solid) axes in LECs after annular ablation.

(D, E) Quantification of recoil velocity **(D)** and relaxation time **(E)** along the short (oriented along the AP axis) and long (oriented along the DV axis) axes in LECs after annular ablations.

(F) Sqh::GFP (Myo II) intensity as a function of junction length. Dots are individual data points (red: DV oriented junctions, blue: AP oriented junctions), black line and error bars: binned mean and SD.

(G) Normalized Myo II intensity along the DV axis, across the anterior histoblast nest. Note the dip in intensity at the dorsal side.

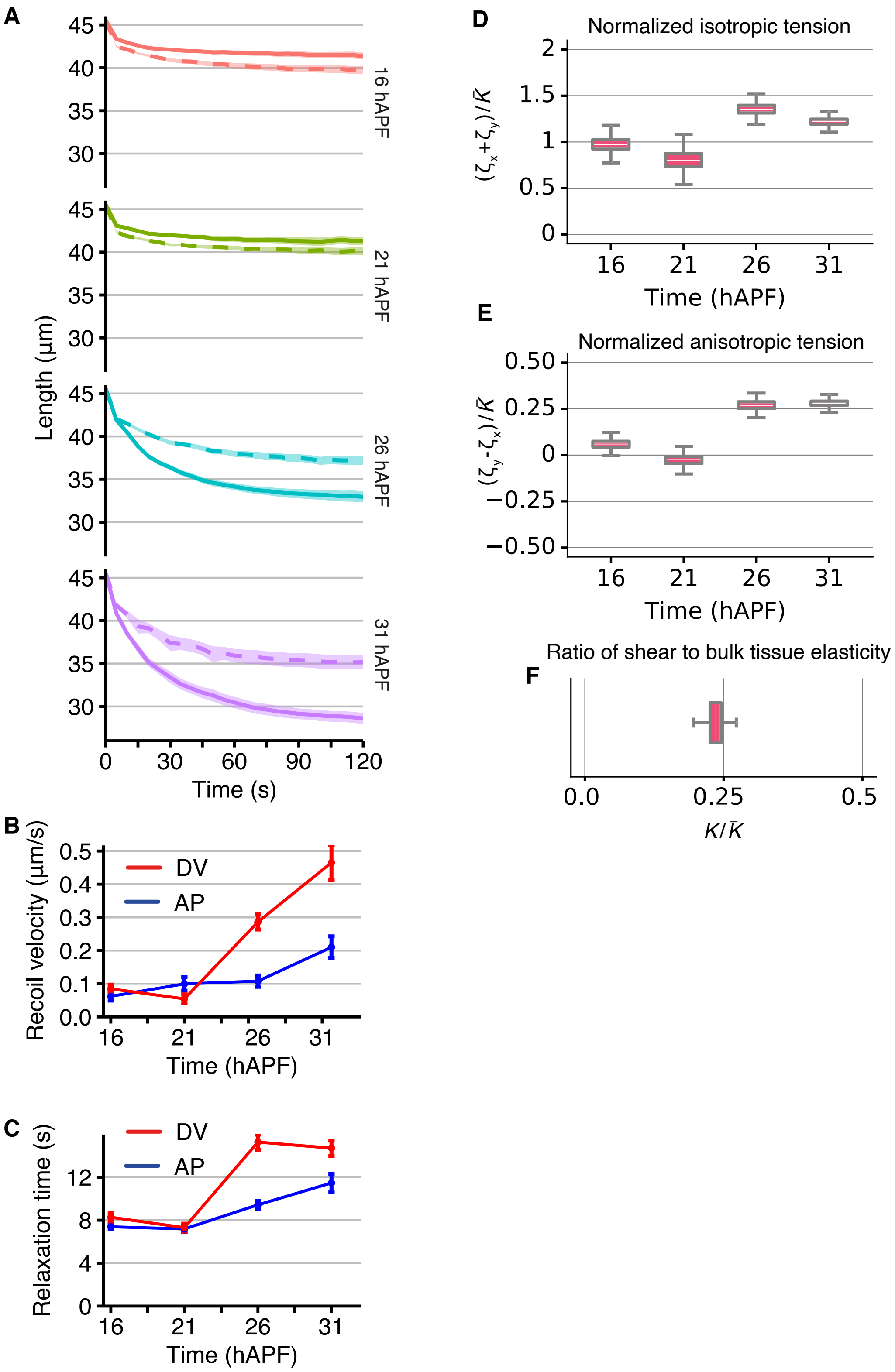

A
Length (μm)
16 hAPF
21 hAPF
26 hAPF
31 hAPF
Time (s)
B
Recoil velocity (μm/s)
DV
AP
Time (hAPF)
C
Relaxation time (s)
DV
AP
Time (hAPF)
D
Normalized isotropic tension
$(\zeta_x+\zeta_y)/\bar{K}$
Time (hAPF)
E
Normalized anisotropic tension
$(\zeta_y-\zeta_x)/\bar{K}$
Time (hAPF)
F
Ratio of shear to bulk tissue elasticity
$K/\bar{K}$

Figure S3. Analysis of annular ablation experiments through histoblast development, Related to Figure 3, and Video S3.

(A) Length of AP (dashed) and DV (solid) axes in excised discs after annular ablation.

(B, C) Quantification of recoil velocity **(B)** and relaxation time **(C)** along the DV and AP axes of excised histoblasts after annular ablation, throughout development.

(D, E) Normalized isotropic (sum of AP and DV tensions, ζ_x+ζ_y, **D**) and anisotropic (difference of DV and AP tensions, ζ_y-ζ_x, **E**) tissue tensions.

(F) Ratio of tissue shear to bulk elastic moduli, obtained from a fit describing the tissue as an elastic material under tension, to measured excised discs deformation following laser ablation (see **Method S1**).

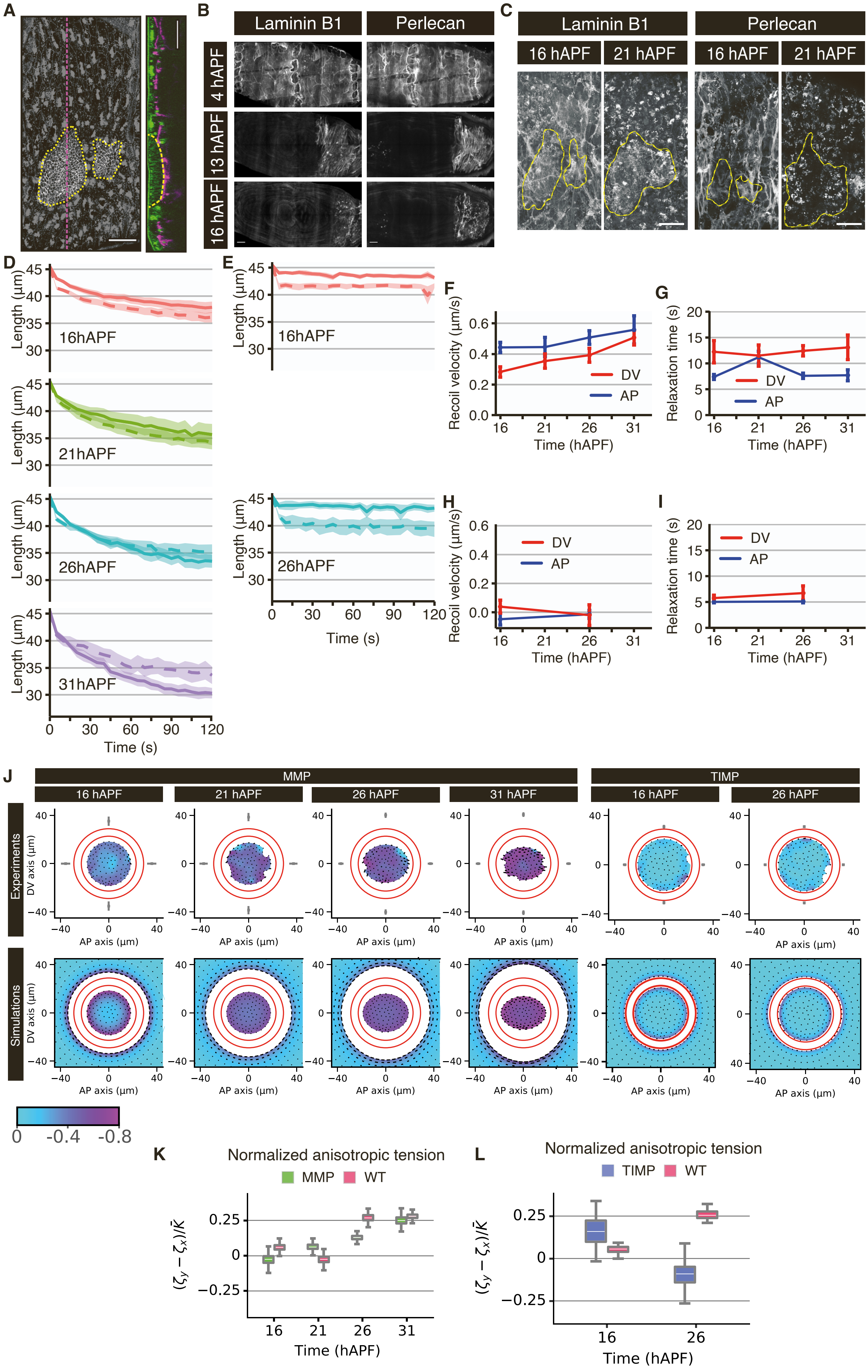

A
B
Laminin B1
Perlecan
4 hAPF
13 hAPF
16 hAPF
C
Laminin B1
Perlecan
16 hAPF
21 hAPF
16 hAPF
21 hAPF
D
Length (μm)
16hAPF
21hAPF
26hAPF
31hAPF
Time (s)
E
16hAPF
26hAPF
Time (s)
F
Recoil velocity (μm/s)
DV
AP
Time (hAPF)
G
Relaxation time (s)
DV
AP
Time (hAPF)
H
Recoil velocity (μm/s)
DV
AP
Time (hAPF)
I
Relaxation time (s)
DV
AP
Time (hAPF)
J
MMP
TIMP
16 hAPF
21 hAPF
26 hAPF
31 hAPF
16 hAPF
26 hAPF
Experiments
Simulations
DV axis (μm)
AP axis (μm)
0
-0.4
-0.8
K
Normalized anisotropic tension
MMP
WT
$(\zeta_y - \zeta_x)/\bar{K}$
Time (hAPF)
L
Normalized anisotropic tension
TIMP
WT
$(\zeta_y - \zeta_x)/\bar{K}$
Time (hAPF)

Figure S4. Tissue mechanics are affected in pupae expressing MMP1 or TIMP, Related to Figure 4, Video S3 and S5.

(A) Snapshot of a pupa expressing *E-cad::Tom* and *Vkg::GFP* (Collagen IV) at 16 hAPF. Yellow dotted lines outline histoblasts. Magenta dotted line indicates position of orthogonal view. Left panel: Maximum projection of *E-cad::Tom*. Right panel: orthogonal projection. Scale bars = 50 μm.

(B) Example confocal images for Perlecan and Laminin B1 basal ECM components during the pre-pupal to early pupal stages. The head everts at 12 hAPF, pushing the abdomen posteriorly and this process is complete by 13 hAPF.

(C) Example confocal images for Perlecan and Laminin B1 basal ECM components during pupal stages. Histoblast nests are outlined in yellow. Scale bars = 50μm.

(D) Length of AP (dashed) and DV (solid) axes in excised cells from MMP1 expressing pupae under the control of *32B-GAL4* after annular ablation.

(E) Length of AP (dashed) and DV (solid) axes in excised cells from TIMP expressing pupae under the control *32B-GAL4* after annular ablation.

(F-I) Quantification of recoil velocity **(F, H)** and relaxation time **(G, I)** of excised histoblasts after laser ablation, along the DV and AP axes throughout development, in MMP1 **(F, G)** and TIMP **(H, I)** expressing pupae.

(J) Experiment (top) and simulation (bottom) deformation plots for excised histoblast discs, at 4 different time points, in pupae expressing MMP1 (left panels) or TIMP (right panels) under the control of *32B-GAL4*. Representation is as in **Figure 3E**. From left to right, n=11, 7, 7, 6, 7, 5 experiments.

(K, L) Normalized anisotropic tension (difference of DV and AP tensions, ζ_y-ζ_x), in pupae expressing MMP1 (green, **K**) and TIMP (blue, **L**), compared to parameters for WT (red, same as **Figure S3E**).

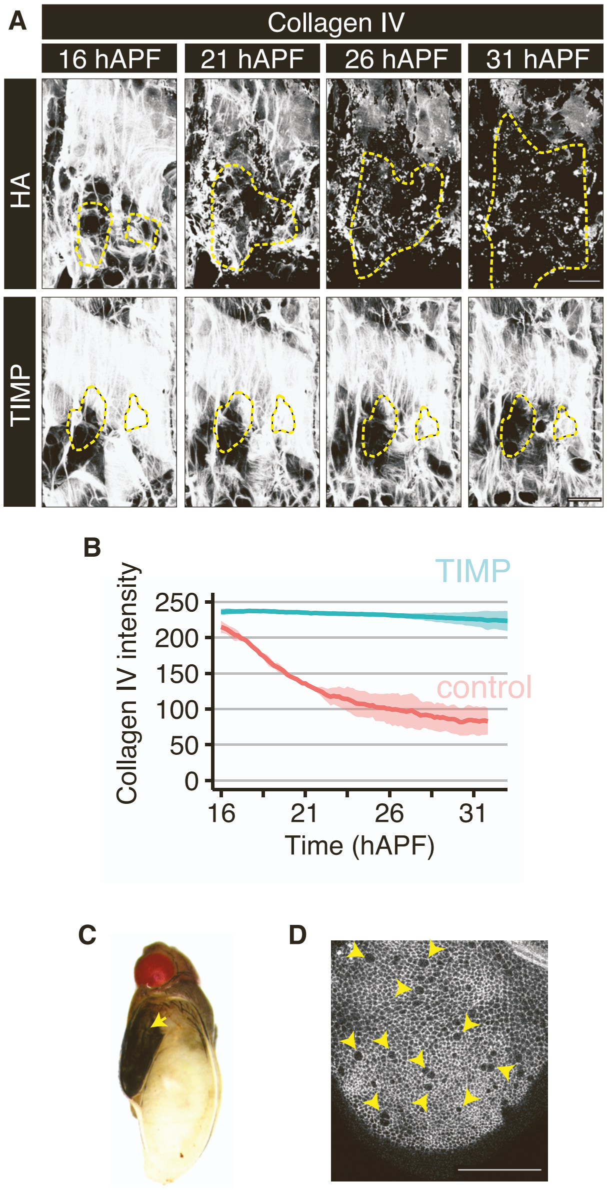
A
Collagen IV
16 hAPF
21 hAPF
26 hAPF
31 hAPF
HA
TIMP
B
TIMP
control
Collagen IV intensity
250
200
150
100
50
0
16
21
26
31
Time (hAPF)
C
D

Figure S5. Blocking ECM degradation in histoblasts with TIMP overexpression does not affect wing disk development, Related to Figure 5 and Video S6.

(A) Snapshots of pupae expressing *Vkg::GFP* (Collagen IV), and overexpressing HA (control) or TIMP under the control of *32B-GAL4* throughout development. Yellow dotted line outlines the histoblasts as seen with *E-cad::Tom* (not shown). Scale bar = 50 μm.

(B) Quantification of the mean intensity of Collagen IV from movies of pupae expressing HA (control) or TIMP under the control of *32B-GAL4* (Error bars: SD, HA n=2, TIMP n=2).

(C) Pharate pupa overexpressing TIMP under the control of *32B-GAL4*. Yellow arrows indicate the normal development of the wing.

(D) Still of wing disc from a movie of a pupa expressing *E-cad::GFP*, and overexpressing TIMP under the control of *32B-GAL4* at the same developmental time as histoblast expansion is inhibited. Note that cell divisions are still occurring (yellow arrowheads). Scale bar = 50 μm.

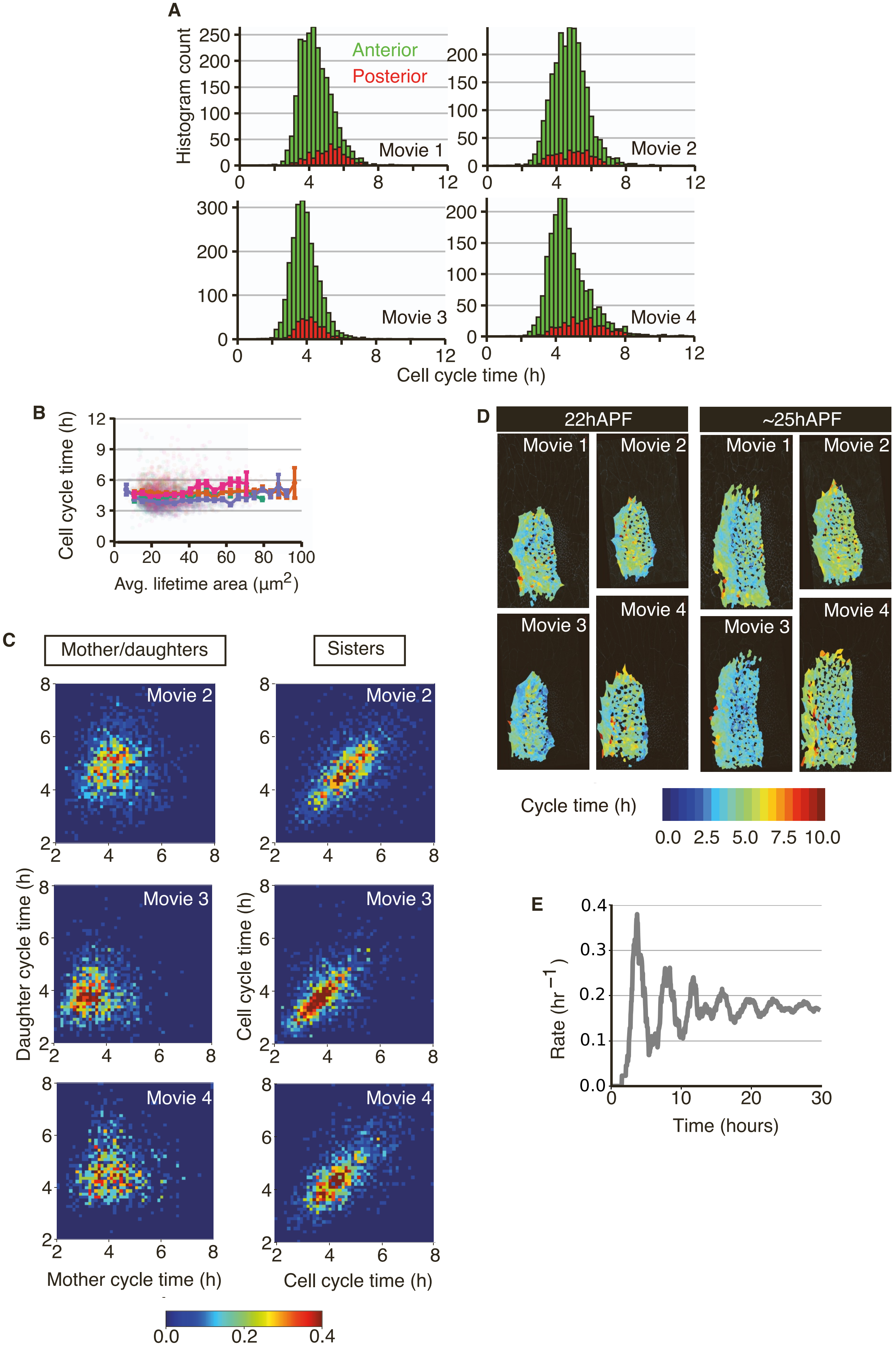

A
Histogram count
Anterior
Posterior
Movie 1
Movie 2
Movie 3
Movie 4
Cell cycle time (h)
B
Cell cycle time (h)
Avg. lifetime area (μm²)
C
Mother/daughters
Sisters
Movie 2
Movie 3
Movie 4
Daughter cycle time (h)
Cell cycle time (h)
Mother cycle time (h)
Cell cycle time (h)
0.0 0.2 0.4
D
22hAPF
~25hAPF
Movie 1
Movie 2
Movie 3
Movie 4
Cycle time (h)
0.0 2.5 5.0 7.5 10.0
E
Rate (hr⁻¹)
Time (hours)

Figure S6. Cell cycle time analyses during abdominal development, Related to Figure 6 and Movie 7.

(A) Histograms of cell cycle times (defined only for cells that are observed appearing and disappearing via division events) in the anterior and posterior nests in movies 1-4.

(B) Cell cycle time as a function of the cell apical area, averaged over the lifetime of the cell.

Solid lines: binned data. Error bars: SEM for the bin. Transparent dots: individual data points. Color code for different movies is as in **Figure 6**.

(C) Probability density of pairs of cell cycle times, where pairs are taken between mother-daughters and sisters. Experimental data from movies 2-4 (see **Figure 6E-F** for movie 1). Cells are taken from the visible anterior nest. High densities of pair along the diagonal indicate high levels of correlation in the pair of cell cycle times.

(D) Snapshots from each wild-type movie with histoblast cells colored according to their cycle time at two intermediate times 22 hAPF and ~25 hAPF. Uncolored (black) cells are SOPs, arrested cells, cells which cannot be tracked from birth to mitosis, or cells with a cycle time larger than 10 hours.

(E) Cell division rate for one realization of a simulation of a cell population growing during 30 h. Cell cycle times are taken from a normal probability distribution with mean 4 h and coefficient of variation 0.2, roughly similar to the experimental distribution in **(A)** (see **Method S1** for details). At t=0 h the simulation starts with 100 cells, at the beginning of their cell cycle. The initial cell synchrony results in damped oscillations in the cell division rate. A moving average is applied as in **Figure 6A**.

A

Anterior nest growth trajectory key quantities	Mean ± SD
(*i*) Starting cell number at 0hAPF	18 ± 2
(*ii*) Cell number after cleavage divisions at 12hAPF	180 ± 20
(*iii*) Estimated cell number end of development 40hAPF	2900 ± 200
(*iv*) Average cleavage divisions per cell (up to 12hAPF)	3.3 ± 0.2
(*v*) Average expansion divisions per cell (14hAPF to end)	4.0 ± 0.2
(*vi*) Average cycle time cleavage - early direct measurements (hours)	2.7 ± 0.3
(*vii*) Average cycle time cleavage - estimate over 0-12hAPF (hours)	3.6 ± 0.2
(*viii*) Average cycle time expansion (hours)	4.5 ± 0.6

B

Parameter	Details on parameters for tissue growth simulations	Base Case Value
N_0	Anterior nests cells at 0hAPF	18
$[ttd]_0$	Mean time to first division at 0hAPF	1.58h
μ_0	Mean cell cycle time up to 3.3hAPF	2.67h
CV_0	Cell cycle time CV up to 3.3hAPF	0.24
μ_1	Mean cell cycle time between 3.3hAPF and 12.5hAPF	4.6h
CV_1	Cell cycle time CV between 3.3 and 12.5hAPF	0.32
$t_{pause\text{-}on}$	Pause in cell division starts	12.5hAPF
t_{main}	Pause in cell division ends	14.7hAPF
η_{sub}	Subset at t_{main} to be noborder ROI	95
$\mu_2(t)$	Mean cell cycle time after 14.7hAPF	Tracks expt. data
CV_2	Cell cycle time CV after 14.7hAPF	0.2
ρ_s	Correlation coefficient of sister cycle times	0.55
s_p	Hill function switch times for arrested cell creation	25.7hAPF
s_a	Hill function switch times for arrested cell creation	24.7hAPF
$h_p = h_a$	Hill function coefficients for arrested cell creation	34
ρ_{SOP}	Probability that a new non-arrested cell is an SOP during SOP generation period	0.09
$t_{SOP\text{-}on}$	SOP generation period start time	20.6hAPF
$t_{SOP\text{-}off}$	SOP generation period stop time	24hAPF

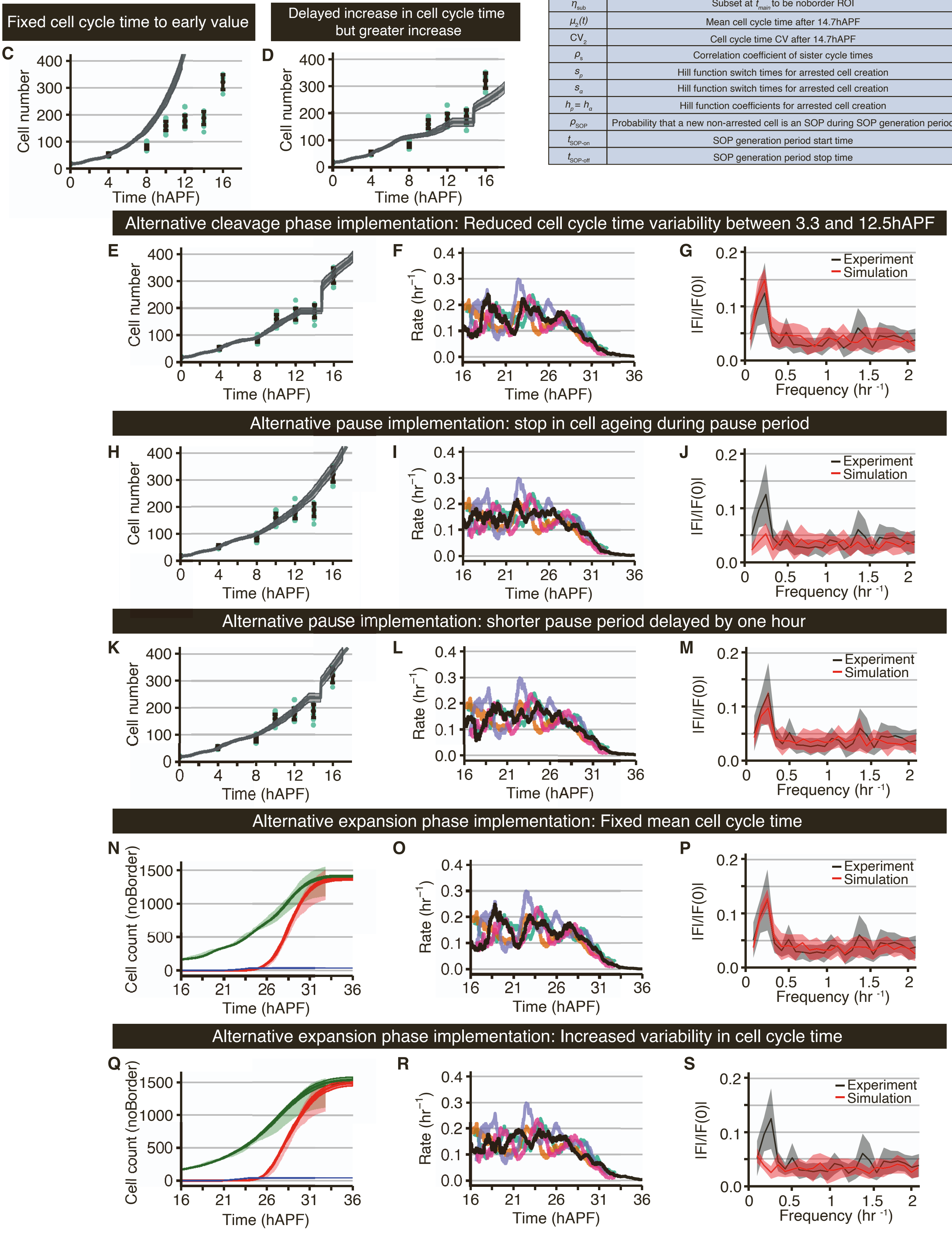

Figure S7. Simulations of histoblast proliferation kinetics, Related to Figure 7 and Method S1.
(A) Anterior nest growth trajectory key quantities. **(*i*)** Anterior only cell counts from **Figure 7C** (n = 15). **(*ii*)** Anterior only cell counts from **Figure 7C** (n = 13). **(*iii*)** Estimate from simulation. The base case simulation reported in **Figure 7** is parameterised to the noborder ROI. Assuming this is a representative subset of the entire anterior nest, the simulation yields an extrapolation for the final cell number in the anterior nest. The reported standard deviation is from n = 10 simulation runs, whose variability is significantly smaller than the experimental variability (see **Figure 7E**). **(*iv*)** Comparing measured cell number at 12 and 0 hAPF and using the formula $N(t) / N_0 = 2^{n_{div.}}$ where N is cell number and $n_{div.}$ the average number of divisions per cell yields this estimate. Compare **Figure 7D**. **(*v*)** Using the estimated final cell number from part (***iii***) and the measured cell number at 14 hAPF (prior to the rapid onset of expansion divisions) yields this estimate. **(*vi*)** Direct measurements available in the first few hAPF (see **Figure 7B**) yields this estimate. Three movies were used, each yielding 20-40 cycle time measurements to generate an average for that movie. The value and uncertainty reported here are the average and standard deviation of those per-movie averages. **(*vii*)** Using the formula $N(t) / N_0 = 2^{n_{div.}}$ as in part (*iv*), the mean divisions per cell $n_{div.} = t / \mu_{\text{cleavage}}$ where t is the time and μ_{cleavage} the mean cycle time of cleavage divisions. Evaluation at $t = 12$ hAPF yields the reported estimate. It is larger than for the direct measurement of part (*vi*). This may indicate that the average cycle time of cleavage divisions really becomes longer, or that some cells do not complete the mean number of cleavage divisions. Either is compatible with the apparent slowing down of cell number increase from 0-12 hAPF which can be seen in **Figure 7C**. **(*viii*)** The coloured data points in **Figure 7B** represent measurements of cycle time during the expansion phase from the four wild-type movies, in the noborder ROI. A simple average of these data points yields the reported value. Colour code represents stages of histoblast growth as highlighted in **Figure7A**.
(B) Table of parameters for the base case of the tissue growth simulation (**Figure 3E-H**). CV: coefficient of variation. Colour code represents stages of histoblast growth as highlighted in **Figure7A**.
(C) Number of anterior cells before 16 hAPF, experiment and simulations. Simulations are as in **Figure 7**, but the mean cycle time is set to be constant and equal to the value measured experimentally before 3 hAPF. The cell number increases much too quickly.
(D) Number of cells before 16 hAPF, experiment and simulations. Simulation results are as in **Figure 7**, but the mean cell cycle time increases to 6.6 h at 4.3 hAPF, instead of increasing to 4.6 h at 3.3 hAPF. This choice leads to a roughly correct number of cells produced around 14 hAPF, but agreement with the measured number of cells over time appears overall less good than in **Figure 7C**.
(E-G) Simulation result with an alternative implementation of the cleavage phase. **(E)** Number of cells in the histoblast before 16 hAPF, as in **Figure 7C**; **(F)** Division rate as a function of time after 16 hAPF, color scheme as in **Figure 7F** and moving average is applied as in **Figure 6A**. **(G)** Normalized absolute value of the Fourier transform of the division rate. Simulation results are as in Figure 7 but the coefficient

of variation of cell cycle time between 3.3 hAPF and 14.7 hAPF has been set to 0.2 instead of 0.32. This results in a peak in the Fourier transform of the division rate which is slightly too high.

(H-J) Simulation result with an alternative implementation of the pause period around 12 hAPF. **(H)** Number of cells in the histoblast before 16 hAPF, as in **Figure 7C**; **(I)** Division rate as a function of time after 16 hAPF, color scheme as in **Figure 7F** and moving average is applied as in **Figure 6A**. **(J)** Normalized absolute value of the Fourier transform of the division rate. Whereas in the base case where divisions were prevented but cell aging continued, here cell aging is paused from 12.2 hAPF and is restarted at 12.7 hours. In this implementation divisions plateau for a short period around 12 hAPF, but the cell number after the pause increases more gradually. The age-stopping pause does not lead to the sudden burst of simultaneous divisions shown in **Figure 7C** and therefore the "re-synchronization", due to setting a large number of cell ages to zero simultaneously, does not occur.

(K-M) Simulation result with an alternative implementation of the pause period around 12 hAPF. **(K)** Number of cells in the histoblast before 16 hAPF, as in **Figure 7C**; **(L)** Division rate as a function of time after 16 hAPF, color scheme as in **Figure 7F** and moving average is applied as in **Figure 6A**; **(M)** Normalized absolute value of the Fourier transform of the division rate. Simulations as in **Figure 7**, but results are obtained with a shorter pause time as the pause starting time is delayed from 12.5 hAPF to 13.5 hAPF; resulting in less pronounced oscillations.

(N-P) Simulation result with an alternative implementation of the expansion phase after 16hAPF. **(N)** Number of cells after 16 hAPF, as in **Figure 7E**; **(O)** Division rate as a function of time after 16 hAPF, color scheme as in **Figure 7F** and moving average is applied as in **Figure 6A**; **(P)** Normalized absolute value of the Fourier transform of the division rate. Simulation results are as in **Figure 7** but the mean cycle time in the main phase is constant at 4.42 h, a value chosen by calculating the average (weighted by number of cells) of the binned cycle time data from **Figure 7B**. There are only subtle differences from the base case **Figure 7F**, for example the second division rate peak is slightly lower.

(Q-S) Simulation result with an alternative implementation of the expansion phase after 16 hAPF. **(Q)** Number of cells after 16 hAPF, as in **Figure 7E**; **(R)** Division rate as a function of time after 16 hAPF, color scheme as in **Figure 7F** and moving average is applied as in **Figure 6A**; **(S)** Normalized absolute value of the Fourier transform of the division rate. Simulation results are as in **Figure 7** but the CV after 14.7 hAPF is doubled to 0.4. The peaks in the division rate become noticeably less sharp and more disordered compared to the base case **Figure 7F**, and this is also reflected in the Fourier transformed data.
