# Supplementary material for: ECM degradation in the Drosophila abdominal epidermis initiates tissue growth that ceases with rapid cell-cycle exit
Source: Curr Biol. 2022 Mar 28;32(6):1285–1300.e4. doi: 10.1016/j.cub.2022.01.045 (PMC8967408; doi:10.1016/j.cub.2022.01.045)
Supplement: Methods S1. Supporting information and methodologies on characterizing Drosophila histoblast growth dynamics, related to Figures 1, 3, 4, 6, 7, S1, S3, S4, S6, and S7 — Figure genotype table, movie segmentation and analysis workflow, and theoretical modeling of histoblast growth dynamics. [file mmc2.pdf]

METHODS S1

FIGURE GENOTYPE TABLE

Figure	Cross	Genotypes
Fig 1E-I; Fig S1B-O; Fig 2A-H; Fig S2A-E; Fig 3; Fig S3; Fig 6A-H, I, J, K; Fig S6; Fig7B, E – G; Fig S7D, E, G, H, J-Q		*y[1] w[*]; TI{TI}shg[GFP] (E-cad::GFP); FRT82B ubi-nlsRFP/+*
Fig 1B, C; Fig 7B-D; Fig S7A-C, F, I	Crossed virgins of<br>*esg^{NP1248}-GAL4/CyO*<br>to males of<br>*UAS-FLP/CyO; act>y+>Gal4 UAS-GFP/TM6b*	*esg^{NP1248}-GAL4/UAS-FLP; act>y+>Gal4 UAS-GFP/+*
Fig 2I-P; Fig S5F, G	Crossed virgins of<br>*E-cad::mKate2*<br>to males of<br>*sqh-GFP.RLC*	*E-cad::mKate2 ; sqh-GFP.RLC*
Fig 4B, D; Fig S4B, C	Crossed virgins of<br>*E-cad::mTomato*<br>to males of<br>*trol (Perl)::GFP* or *lanB1::GFP*	*trol (Perl)::GFP/+ ; E-cad::mTomato / +* and *E-cad::mTomato / lanB1::GFP*
Fig S5A, B		*E-cad::mTomato, vkg-GFP*
Fig 5A		*Mmp1.GFP ; +/+*
Fig 4A-D	Crossed virgins of<br>*w^{iso}* to<br>*vkg-GFP, E-cad::mTomato*	*vkg-GFP, E-cad::mTomato /+*
Fig 4E, G-J, L; Fig S4F, G, J, K; Fig 5C	Crossed virgins of<br>*E-cad::GFP , tubG80^{ts}; 32B-GAL4*<br>to males of<br>*UAS-MMP1 (III)*	*E-cad::GF, tubG80^{ts}/+ ; 32B-GAL4/UAS-MMP1*
Fig 4F-I, K, M; FigS4H-J, L; Fig 5A-C; Fig S5C, D	Crossed virgins of<br>*E-cad::GFP ; 32B-GAL4*<br>to males of<br>*UAS-TIMP (III)*	*E-cad::GFP/+ ; 32B-GAL4/UAS-TIMP*
Fig S5A, B	Crossed virgins of	*vkg-GFP, E-cad::mTomato/+ ; 32B-GAL4/UAS-HA*

	+ ; 32B-GAL4 to males of *vkg-GFP, E-cad::mTomato; UAS-HA* or *vkg-GFP, E-cad::mTomato; UAS-TIMP*	*vkg-GFP, E-cad::mTomato/+ ; 32B-GAL4/UAS-TIMP*
Fig 5A		*E-cad::GFP, tubG80^{ts}/+ ; 32B-GAL4/UAS-TIMP* *MMP1-GFP* *E-cad::GFP; 32B-GAL4/UAS-MMP1 RNAi, MMP2 RNAi*
Fig 5B		*E-cad::GFP, tubG80^{ts}/+ ; 32B-GAL4/UAS-HA* *E-cad::GFP, tubG80^{ts}/+ ; 32B-GAL4/UAS-TIMP* *E-cad::GFP; 32B-GAL4/UAS-MMP1 RNAi, MMP2 RNAi*
Fig 5D, Fig 6G, I		*esg^{NP1248}-GAL4, UAS-FLP/+; act>y+>Gal4 UAS-GFP/UAS-GFP-E2F1$_{1-230}$ RFP-CycB$_{1-266}$*
Fig 5D		*tubG80ts/Ubi-GFP-E2F1$_{1-230}$ Ubi-RFP-CycB$_{1-266}$; 32B-GAL4/UAS-TIMP*
Fig S5E		*tubG80ts/Ubi-GFP-E2F1$_{1-230}$ Ubi-RFP-CycB$_{1-266}$; 32B-GAL4/+*

MOVIE SEGMENTATION AND ANALYSIS

Overview of the segmentation and tracking pipeline

The aim of the image analysis pipeline was to identify, track and classify individual cells within the developing histoblast nests. The image processing was performed on projected cell surfaces which were subsequently segmented (skeletonized) first before being tracked. These two steps were designed to be complementary. The segmentation informed the tracking which highlighted issues with the segmentation. Any errors detected through the cell tracking allowed the segmentation to be corrected and improved. The subsequent analysis in TissueMiner required a segmented and tracked cell image sequence which was of sufficiently good accuracy to provide reliable and meaningful interpretations. Therefore, in addition to an automated tracker, a set of interactive tools were developed to provide the option to manually correct the segmented image sequences and the results from the tracking.

Post-imaging

Input: Microscope creates .czi file with Z-stacks in time

Open .czi Zeiss Microscopy Image file in ImageJ/Fiji and export as .btf (BigTif)

Output: BigTif: Z-stacks in time

Projection
Input: Btf file saved in ImageJ/Fiji, full Z-stacks for each time point
Open surface projection programme in Matlab
Use GUI to manually identify the desired projection regions in every 10th frame
Projection programme automatically interpolates projection for the frames in between
Output: Tif: single projected slice for each time point

Skeletonization
Input: Tif: single projected slice for each time point
Using Skeletor, set threshold to between 0.2-0.3
Generates separate .tiff files of individual skeletonized frames
Use ImageJ to concatenate frames
Output: Tif: skeletonized image for each time point (Skeleton v1)

Manual correction
Input: Tif: Skeleton v1, and Tif: single projected slice for each time point
Add Matlab files in Manual Correction folder to Matlab file path
Add projection and Skeleton v1 images to Matlab file path
Run SkeletonStart.m
Load Projection Tif stack
Load Skeleton Tif stack
Manually correct as many missing or extra junctions as possible using hotkeys
Save corrected skeleton
Output: Tif: Corrected skeletonized image for each time point (Skeleton v2)

Preliminary tracking and further manual correction
Input: Tif: Corrected skeletonized image for each time point (Skeleton v2)
Add Matlab files in Tracking Correction folder to Matlab file path
Add projection and Skeleton v2 images to Matlab file path
Run TrackingCorrectionStart.m
Load Projection Tif stack
'Find cells', Matlab finds the centroids of uploaded skeleton
'Tracking', preliminary tracking identifies points where track is lost due to skeleton errors
'Problems' selected from drop-down menu, this will automatically start selecting lost tracks
Manually correct the junction errors identified
Click 'Export Skeleton' to save corrected Skeleton v3
Binarize stack in ImageJ
Use 'SaveAsSingleTIFFs' Matlab programme to separate into individual tifs
Output: Individual Tif files, corrected binarized skeletonized image for each time point (Skeleton v3)

Tissue Analyser skeleton processing
Input: Skeleton v3: corrected binarized skeletonized image for each time point
Transfer individual Tifs of Skeleton v3 into Tissue Analyzer
Use the Detect Bonds (Save Watershed) function in Tissue Analyser to export Tissue Miner-compatible skeleton into individual folders
No blur and no removal of cells with x pixels

Use Mac terminal to transfer the individual Tifs into individual folders within the same directory
CODE

```
for d in */ ; do (cd "$d" && pwd && cp handCorrection.tif"../dv$(basename "$d").tif"
);done;
```

Concatenate individual tifs to a single Tif stack in ImageJ
This creates an RGB Tif stack in time, use ImageJ to create 8-bit image
Check for any new errors that may have been introduced by Tissue Analyser processing by repeating the preliminary tracking correction
Correct errors and repeat Tissue Analyser processing
Output: Tif: Corrected skeletonized image for each time point (Skeleton #4)

Tracking in Matlab
Input: **Tif file, corrected skeletonized images over time (Skeleton #4)**
Alter AutoTrackingStart.m code to ensure it has the correct filename of skeleton #4
To start tracker:
CODE:

```
cd ~/filepath/Tracker/data
export MATLABPATH=/home/ainsli01/Documents/Tracker:/home/ainsli0
1/Documents/Tracker/data
nohup matlab -nodesktop -nodisplay -noFigureWindows -nosplash -r
"cd('/home/ainsli01/Documents/Tracker/data');AutoTrackingStart; quit" -logfile logfile.out <
/dev/null &
```

This will generate three .tif stacks over time, TrackedCellsRGB: each cell colored with a unique cell I.D., DivisionsRGB: divisions highlighted in blue, ErrorsRGB: errors highlighted in red
Output: RGB Tif stacks of Tracked Cells, Divisions and Errors

Using ErrorRGB output in to correct skeleton
Input: Skeleton v4 and ErrorRGB Tif stack
In ImageJ, separate red channel from ErrorRGB Tif stack, and subtract the skeleton resulting in an image with only red cells
Binarize red cells and use 'Analyze Particles' in ImageJ to generate list of coordinates and time points of red cells
Open skeleton in Image J, use 'SpecifyArea' macro to find errors in list of particles:

IMAGEJ MACRO CODE:

```
macro"SpecifyArea [c]"{run("Specify...");}
```

Enter x,y,t coordinates, automatically takes you to error
Annotate list of errors, label as either: tracking, division, skeleton or edge (edge errors can be ignored)

Output: Skeleton #5 and a list of annotated errors

If skeleton errors above 50, RETURN TO FIRST STAGE OF MANUAL CORRECTION
If skeleton errors below 50, correct skeleton errors and divisions in ImageJ
Correcting missed divisions:

- Using the 'SpecifyArea' macro go through Divisions Tif stack finding the errors annotated as 'Divisions'
- Use 'pick color' to select division blue color, and use 'fill' function (4-connected) to fill just-divided cells that have been missed

Correcting skeleton:

- Correcting raw skeleton tiff stack using 1-pixel thick paintbrush in ImageJ, taking care to only generate junctions that are 1-pixel thick
- Copy-paste new junction into TrackedCellsRGB and Divisions tiff stacks
- Making sure to correct the colors by using the 'pick color' and 'fill' function in ImageJ

Make Tracker output compatible with Tissue Analyser and Tissue Miner
Input: Projected image stack and TrackedCellsRGB and Divisions image stacks
Open 'SaveAsTissueMiner.m' and make sure filenames are correct
Run 'SaveAsTissueMiner'
Output: individual folders containing an individual projection, TrackedCellsRGB and division image for each time point

Correct Tracks using Tissue Analyser
Input: individual folders containing projection, TrackedCellsRGB and division image for each time point, as well a list of tracking errors
Fix broken and swapped tracks using the tools in Tissue Analyser
Output: corrected TrackedCellsRGB files

Tissue Miner analysis and further Quality Control
Input: corrected Tracked Cells and Divisions
Use Tissue Miner to highlight cell I.D.s that disappear in red in final frame visible, these are either apoptoses or tracking and skeleton errors
In ImageJ, separate red channel
Binarize red cells and use 'Analyze Particles' in ImageJ to generate list of coordinates and time points of red cells
Save list of red cells as an Excel file
Use 'SpecifyArea' macro to find errors and annotate list of particles
Correct errors as described in Steps 11 and 13
Output: individual folders containing an individual projection, fully corrected TrackedCellsRGB and division images for each time point

RETURN TO **MANUAL CORRECTION** if there are significant remaining skeleton and tracking errors
Otherwise, continue Tissue Miner data analysis

Projection

The cell projection tool was developed to provide a semi-automated means of creating cell surface projections of histoblast and LEC image stacks. This was necessary as strong cell signal

could often be found above and below the main cell surface which sometimes required manual corrections. The vast data volumes to be processed required an efficient approach with as little manual intervention as possible and a convenient approach to correcting the surface projection where necessary. We carried out the corrections on a sub-sampled image sequence with subsequent spatial and temporal interpolation over the whole sequence. A tool featuring a graphical user interface was developed for this purpose in Matlab.

After sub-sampling in the time domain (typically 1/10 frames) to generate key frames, the maximum intensity projection over the z-depth provided a first estimate where surface markers would be placed at depth levels expressing intensity values in the top. Only a subset of markers was used to keep the number of markers low. These markers could be deleted and added interactively by the user [s1]. Markers could also be copied to the next frame and translated to correct for a z-shift. Markers would then be used to calculate a depthmap by iteratively averaging marker depth values greater than zero for all image values resulting in a smooth, interpolated depthmap.

The surface for the whole sequence was generated from the depthmaps of key frames by means of linear interpolation in the time and spatial domain. Cell surface intensities were obtained by the max intensity in the vicinity of the interpolated surface from the original 3D image volume for each time frame. The cell surface was stored as a 2D image sequence which was then imported into the image segmentation and skeletonization step.

Skeletonization using Skeletor

For the initial segmentation and ground-truthing for the machine-learning segmentation algorithm, we developed a filter-based watershed algorithm in Mathematica which we called Skeletor. Initially, projections were filtered using seven filter kernels, which are as follows: to highlight edges we developed a new convolution matrix which we termed the davisfilter, where the mean intensity of a 3x3 pixel kernel was compared to the mean intensity of an 11x11 pixel kernel, if the mean for the smaller kernel was greater than for the larger kernel then the origin pixel intensity was kept, if not then the origin pixel value was set to zero. The next filter was a modified salt and pepper filter called saltpepper which aimed to de-noise the images, here the three largest and smallest pixel values of an 11x11 kernel were removed, and the nearest pixel value to the mean of remaining pixels was used as the origin pixel intensity value. The third filter was a contrast enhancing median filter which we called medianfilter, where if the origin

pixel intensity was greater than the median for an 11x11 pixel kernel then the origin value was kept, if not then it was replaced with 0. The fourth and fifth filters both calculated the median and the median deviation for an 11x11 pixel kernel, and kept the origin pixel value if it was greater than the median plus the median deviation, if not then filter four, which we termed MADfilter, would subtract the median for the 11x11 kernel to improve contrast; filter five which we termed MADsmoother, would replace the origin pixel value with the median of the 11x11 kernel, smoothing the background. Finally, the sixth and seventh filters were both in-built Mathematica functions which smooth edges, the sixth being an image convolution with a Shen-Castan Matrix function with an exponential radius of 5 pixels and the seventh the CurvatureFlowFilter function with curvature time of 1. The average intensity for each pixel was then calculated from all seven filtered images, and then convolved with a Shen-Castan Matrix with a 2 pixel radius.

Once the image had been filtered, it was segmented using Mathematica's gradient descent watershed algorithm, where junctions were merged if the minimum boundary height was below a user-defined value, normally in the range of 0.2 – 0.3. The perimeter of each component was then obtained and all the perimeters combined to produce the initial skeleton. To remove erroneous segmentation two tests were performed on each junction. The first test compared intensity of junctions to the interior of the cell to remove 'phantom' junctions; specifically, the median plus quartile deviation intensity for the cell interior was measured and set as a threshold, if the median intensity for each junction was below this value it was removed. The second test removed junctions that had a meandering topology; specifically, the length of a straight line between each junction vertexes was divided by the length of each junction and if the value was below 0.85 (i.e. junctions were 15% longer than a straight line) then they were removed. Both of these tests mainly removed erroneous junctions in the LECs with few junctions in the histoblasts being removed.

Skeletonization using machine learning

For the movies wild-type1-3, skeletonization was carried out using Skeletor. For wild-type4, we used a UNet Neural Network [s2] trained using wild-type1-3 data segmented with Skeletor and manually corrected. We used a 3D version of the network treating the time as a z component of input data. Different training conditions, varying the loss functions, optimizer and learning rate, were tested. After training, predictions were obtained from new images, then used in Tissue Analyzer to segment the epithelia.

Manual Correction of skeletons – Skeleton Correction Tool

A second interactive tool was developed to verify and correct the result of the skeletonization in an efficient manner. The tool was written in Matlab and featured a graphical user interface and the ability to use a Wacom Tablet to correct missing or extra cell junctions. The tool allows the overlay of original cell surface and skeletonized images, also blending these image layers together. To aid this process, cells could be identified from the skeletonized image and their centroid position was marked on the surface image. Potential segmentation errors could be easily spotted as the markers were off the cell center. In addition, a previous developed automated cell centroid seed tracker [s3] was incorporated into the tool as a means of quick, preliminary tracking to highlight potential issues caused by segmentation errors. This would support the user to identify and correct such errors.

The tracking results from the offline tracker could also be shown as an additional image layer. Drawing and erasing of cell junctions on the skeletonized image could be performed using a pen on the Wacom Drawing Tablet. Hotkeys allowed the fast switching between different editing modes which contributes to an efficient workflow. The corrected skeleton image sequence was exported for the automated offline tracking step.

In order to quantify the changes at each stage of corrections in Figure S1B, a thickened version of the original Skeletor output skeleton was created was subtracted from the wild type movie post 'Manual Correction' and post 'Manual Tracking Correction' in ImageJ. The 'Analyze Particles' function was used in ImageJ to count the remaining pixels.

Automated Tracking procedure – Tracking Tool

The tracking algorithm was designed to fully automatically track cells in the projected skeletonized images. This was achieved in a three-step process and was performed frame by frame for all cells in the sequence. The first iteration tracked cells by identifying suitable matching cell candidates by means of the best-fit of the cell area. Only cells with a good-fit were included in this sequence with the aim is to get a first estimate of the cell surface motion. In a second step, the motion (flow) field of the whole cell surface was calculated through the interpolation of individual movement vectors from the initial tracking results. In the third step the tracking is guided by the flow estimates. Finally, the tracked cell sequence was further

processed to determine the cell lineages and divisions and exported as a number of different image sequences. The following paragraphs describe these steps in more detail:

1. Find initial tracks

The initial tracking is performed by identifying potential matching candidates in the next image frame. The area of each cell in a frame is obtained from the labelled binary skeleton image. Any cell in the next frame that slightly overlaps with the source cell area was a potential candidate. For each target candidate, the cell areas were aligned by centroid position. Similarities in terms of the overlapping cell area were used to determine a good fit. The confidence c (a metric for a good-fit) was calculated for each candidate as the ratio between the overlapping aligned cell area and the total combined area of source and target cells.

$$c = 1 - \frac{\sum A \cap B}{\sum A \cup B}$$

This would exclude fast moving smaller cells that were not overlapping which made the flow guided tracking step necessary.

Once a list of candidates and their confidences was established, the best candidate for each source cell was identified amongst the several potential target candidates. Each source cell was assigned the target cell with the highest confidence first. This is performed iteratively for all source cells for each iteration starting with the highest confidences first and iterating down as long as the confidences are above the minimum threshold of 0.7. The source cells were thus competing for target cell candidates as neighboring cells could have the same potential target candidates which ensured that the best candidate was assigned for each source cell. It is important to note that in the first iteration of the tracking we only used cells with high confidences ≧0.7, i.e. cells which we could be confident about to be correct. In a final step new track IDs were assigned to cells which were not paired due to having a low confidence value.

This first tracking step returned an image matrix consisting of 16-bit grayscale images. The color zero (black) denoted the cell boundaries. Each cell area had a uniform numerical value which denoted the track ID. The track ID remained the same for matching cells in subsequent frames. New cells or broken tracks received with a new track ID.

2. Calculate the flow field estimate

In this step an interpolated flow field from the cell movements of the previous tracking step was calculated. The displacement vector for each cell was calculated from the centroid positions of the tracked cells. The flow field was calculated for each position in the image matrix by a weighted average of all the displacement vectors with a weighting factor which was the inverse of the distance to all the other cell positions in the frame. The influence of nearby cells is thus much greater than cells at greater distances. The interpolation was completed when all the values in the image matrix had been calculated. This step returned the interpolated flow field for each frame in the sequence.

3. Re-track all cells by using the flow estimate

The final cell tracking was performed by taking into account the flow estimates of cells. As in the first tracking step, any cell in the next frame that slightly overlapped with the source cell area was a potential candidate. However, in this step, the source cell area was translated by the average flow field displacement of that area obtained from the initial tracking. The overlap between the translated area of source and target cells was then calculated as in step 1 above.

The assignment process was more complex as in the first tracking iteration. The aim was to find the best candidate for each source cell amongst the several potential target candidates. However, this was performed over several steps as all the source cells needed to be paired, not just the most obvious fits. During the first step, each source cell was assigned the candidate cell with the highest confidence first. This was run competitively for all source cells for each iteration starting with the highest confidences first and iterating down as long as the confidences were above the minimum threshold of 0.2. The source cells were thus competing for target cell candidates as neighboring cells could have the same potential target candidates which ensures that the best candidate was assigned for each source cell.

In a second step, the above process was repeated for not yet assigned source cells by going through the remaining candidates. In a third iteration, the not yet assigned source cells were assigned by distance up to a max distance to target cells.

Finally, any unassigned cells were treated as new cells which were assigned a new cellID and denoting the start of a new track sequence.

This second tracking step returned an image matrix consisting of 16-bit grayscale images. The color zero (black) denoted the cell boundaries. Each cell area had a uniform numerical value denoting the track ID. The track ID remained the same for a matching cell in subsequent frames. New cells or broken tracks received with a new track ID.

4. Exporting tracking result

The track sequence was exported as an RGB image sequence whereby each cell track was given a unique RGB color. Divisions were identified by a sudden increase in cell size in a tracked sequence which also coincided with a new cell track emerging in a subsequent frame in its locality. The ratio of the cell size change between frames was used a measure to identify divisions. A cell which underwent such transformation was identified as the mother cell while a new cell it is vicinity was labelled as the daughter cell. From these divisions, a lineage sequence could be created, highlighting cell divisions in different shades of blue. Potential errors in the lineage emerged when new cells did not originate from a division event or when the lineage was not clear. Cells with such issues were labelled in red in an additional 'Error' sequence and exported for further manual inspection and correction if deemed necessary.

5. Manual Correction of Tracking

The output from the offline tracker, including the lineage and error sequences were imported into ImageJ for further inspection and correction. Cells highlighted in red 'Error', i.e., cells with a new cell ID that do not originate from a division were labelled manually using the 'Analyze Particles' function in ImageJ. This creates a list of red cells that are manually checked and annotated according to the type of error, either a 'Tracking' error due to the cell migrating quickly, or a 'Division' error where Tracker has failed to pick up a division, or a 'Skeleton' error that remains. Using this information, each type of error was corrected in a different way. If the number of remaining skeleton errors was significant (above 50), then only the input skeleton would be corrected, and the automatic tracking process would start again. If the number of remaining skeleton errors was below 50, then all the errors would be corrected manually using the coordinates lifted from the error layer. First the 'Division' errors were corrected in the division layer using the 'fill' function in ImageJ to fill daughter cells of missed divisions in the correct shade of blue. Then the 'Skeleton' errors were corrected in both the division layer and the unique cell ID. layer using ImageJ. White lines were added or removed manually in the division channel in ImageJ, taking care to ensure all lines were maintained at a 1-pixel thickness. Then the affected area was copied and pasted into the unique cell ID.

channel to ensure the white lines were the same. Using the fill function ensured the colors in the unique cell ID. channel were unaffected. Finally, the 'Tracking' errors were corrected in Tissue Analyser [s4,s5]. Once the affected coordinates were found, it was possible to swap tracks around, join truncated tracks or create new cell IDs wherever necessary. The corrected lineage sequence was finally converted and exported into a format that was readable by Tissue Miner.

Tracking validation and quality control

All sequences went through a thorough process of quality control. The tracker supported this approach by highlighting potential issues in an 'error' sequence. Once the skeleton errors were corrected, any subsequent errors were due to tracking errors and cell division errors. A typical sequence featured between five and six thousand cell tracks. The tracker would typically highlight a total of around 300 (5-6%) of these tracks as having potential issues which required inspection: Approx. 1-2% of the tracks had tracking errors whereby the tracker did not correctly identify a matching cell. These could be amended by linking up broken tracks or swapping tracks. However, most issues, approx. 3-4% of all the tracks arose at or in the vicinity of dividing cells when mother or daughter cells were not identified, swapped or not classified as being part of the lineage. Not all these issues were genuine errors that required correction, but all these issues required inspection by an operator to ensure a high level of quality control.

Tissue Miner Analysis

Once these correction steps have been completed using ImageJ and Tissue Analyser, Tissue Miner was then used to extract data from the tracked cells;[s5,s6] see Supplementary Theory for details. Tissue Miner was also used to identify any remaining errors by detecting loss of cell I.D., as well as detecting with an anomalously short 'cycle time' in between divisions. Any errors that were detected using these criteria were corrected in the manner described under 'Manual Correction of Tracking', and then the final data is re-entered into Tissue Miner.

Temporal alignment of wild-type movies

A salient feature of abdomen development is the formation of the sensory organs of the adult abdomen, which are mechanosensory bristles that arise through multiple stereotypical rounds of asymmetric cell divisions from a single progenitor, the Sensory Organ Precursor (SOP) [s7]. We tracked the SOP lineages and excluded them from all subsequent analysis on histoblast growth and proliferation (Figure S1E). However, we noticed that the emergence of SOPs over time, identified by the initial asymmetric division, followed a sigmoidal temporal distribution

that could readily be fitted by a Hill function (Figure S1F-J). To allow comparison of the growth parameters, these curves were used to temporally align our wild type movies (wild-type1-4) (Figure S1J).

SUPPLEMENTARY THEORY

In this Supplementary Theory, we first discuss analysis of segmented and tracked movies of histoblast nests (section 1). We then describe simulations for the dynamics of the number of cells in the histoblast (section 2), and a continuum elastic theory, simulated using a finite-element method, that we have used to analyse deformations following laser ablation experiments (section 3).

1. Analysis of tracked and segmented movies

Each tracked and segmented movie is stored on disk in the form of a consecutive series of folders, one for each frame of the movie, to be passed on to the Tissue Miner analysis package[S5,S6]. Each frame-folder contains three images: the original confocal projection image, which is used only as a background on which to plot analysed quantities (e.g., Figure 1e); a tracking mask, comprising a skeleton in which each cell is given a unique colour which persists over time, encoding the tracking; a division mask, comprising a skeleton in which each pair of newly-divided daughter cells in a given frame is highlighted.

Tissue Miner parses these images, converting the information they encode into a series of tables describing the trajectory of the movie: vertex positions in each frame, cell centroid coordinates, cell areas, division events, cell extrusions, and so on. Exhaustive detail is given in Refs.[S5,S6] which we will not recapitulate here.

For the purpose of our analyses, three aspects of Tissue Miner are particularly useful. First, the table *cellsDB*, which contains 'dynamic' information on each cell through time – the position and area of a cell in each frame, for example. Second, the table *cellinfoDB* contains meta-information on each cell. For example, whether and when a cell appears by a division event, or is present from the start of the movie, and whether a cell disappears by dividing itself, or by exiting the field-of-view. Thirdly, Tissue Miner contains a number of ready-made scripts which automate common types of analysis. For example, such scripts can be used to obtain the decomposition of tissue deformation into various cellular contributions (see Figure 1).

Tissue Miner's data can be interrogated and manipulated in both the R and Python languages (we have mostly used R), which allows to create customised analyses such as our quantification of arrested cell appearance (Figure 6J).

1.1. **Definition of noborder ROI.** During development, cells both enter and leave the movie field of view. For much of our analysis, it is convenient to work with a population of histoblast cells that are not affected by this. We therefore define the 'noborder' ROI. It contains histoblast cells that are members of lineages of which no member encounters the boundary of the field of view at any point in the movie. In practice, the noborder ROI ends up being a subset of the anterior histoblast nest, since the posterior nest disappears entirely from view during the course of the movie.

1.2. **Isotropic shear decomposition.** Following Ref.[S5], we perform a decomposition of the tissue area expansion rate of the noborder ROI, $v = \frac{1}{A}\frac{dA}{dt}$ with A the tissue area, as follows:

$$v = \frac{1}{a}\frac{da}{dt} + k_d - k_e \ , \qquad [1]$$

where a is the average cell area, k_d is the cell division rate and k_e the cell extrusion rate. This decomposition can be understood from the simple relation:

$$A = a \times N \ , \qquad [2]$$

where N is the number of cells in the tissue. Differentiating this relation gives Eq. [1], following the identification $\frac{1}{N}\frac{dN}{dt} = k_d - k_e$.

Integrating Eq. 1 between t_0 and t yields the cumulative shear relation

$$\ln \frac{A(t)}{A_0} = \ln \frac{a(t)}{a_0} + \int_{t_0}^{t} dt k_d - \int_{t_0}^{t} dt k_e \ , \qquad [3]$$

with $A_0 = A(t_0)$ and $a_0 = a(t_0)$. In Figures S1N-Q different terms of Eq. [1], calculated from finite differences between movie frames, are plotted as a function of time. Summing these contributions yields a cumulative shear decomposition plot (Figures 1F-I) which corresponds to Eq. [3], up to time discretization errors.

1.3. **Cell elongation calculation.** In laser ablation experiments reported in Figures 3 and S4, we obtain the change in cell elongation before and after ablation. We use cell elongation obtained from cell segmentations, as described in Ref.[S6]. Briefly, the cell centroid $\mathbf{r}_c$ is defined by an integral over all points $\mathbf{r}$ within the cell, which has area A_c:

$$\mathbf{r}_c = \frac{1}{A_c} \int \mathbf{r} \, \mathrm{d}A \ . \tag{4}$$

The components $Q_{xx} = -Q_{yy}$ and $Q_{xy} = Q_{xy}$ of the 2D nematic tensor describing cell elongation are calculated as follows:

$$\begin{aligned} Q_{xx} &= \frac{1}{A_c} \int \cos(2\phi) \, \mathrm{d}A \\ Q_{xy} &= \frac{1}{A_c} \int \sin(2\phi) \, \mathrm{d}A \ , \end{aligned} \tag{5}$$

in which ϕ is the angle between the vector $\mathbf{r} - \mathbf{r}_c$ and the x axis (approximately the anteroposterior axis of the animal in our case). The elongation norm (Figure 6D) is defined as $|Q| = \sqrt{Q_{xx}^2 + Q_{xy}^2}$.

1.4. **Calculation of cell cycle time correlations.** To calculate correlations between cell cycle time of pairs of cells (Figure 6), we proceed as follows. We consider the set of dividing cells of the anterior nest which can be tracked from their birth to their division, D, and for every cell $x \in D$ we denote the cell cycle time τ_x. For a given pairing condition (for instance, mother and daughters), we find all cell pairs (x, y) for which the pairing condition is satisfied, and the Pearson correlation coefficient ρ is then calculated as:

$$\rho = \frac{1}{N} \frac{\sum\limits_{(x,y)} (\tau_x - \tau_X)(\tau_y - \tau_Y)}{\sigma_X \sigma_Y} \ , \tag{6}$$

$$\tau_X = \frac{1}{N} \sum_{(x,y)} \tau_x \ , \tag{7}$$

$$\tau_Y = \frac{1}{N} \sum_{(x,y)} \tau_y \ , \tag{8}$$

$$\sigma_X^2 = \frac{1}{N} \sum_{(x,y)} (\tau_x - \tau_X)^2 \ , \tag{9}$$

$$\sigma_Y^2 = \frac{1}{N} \sum_{(x,y)} (\tau_x - \tau_Y)^2 \ , \tag{10}$$

where N is the number of pairs (x, y). In practice we use the scipy.stats.pearsonr function. By construction, $\rho = 0$ if all possible pairs in D are included, and $\rho = 1$ if only identical cells are included (i.e., considering only pairs (x, x) for $x \in D$).

To obtain the Spearman correlation coefficient ρ_S for a given pairing condition, we find all cell pairs (x, y) for which the pairing condition is satisfied. We then directly use the scipy.stats.spearmanr function on this data.

We also calculated correlation coefficients defined such that, for every cell x, a cell y is chosen randomly among possible pairs (for instance, when a mother has two daughters, only one daughter is associated to each mother). For different pairing conditions, we formed set of pairs defined in this way a 100 times, and calculated the average correlation coefficients. We found similar results with this method than by taking into account all possible pairs: the Spearman correlation coefficient was $\rho_S = 0.06 \pm 0.03$ for mother-daughters (mean$\pm$standard deviations are obtained from 4 WTs).

2. Simulation of tissue growth

Here we describe simulations conducted to model proliferation of the anterior-nest histoblast cells. The simulations do not attempt to resolve spatial behaviour, rather they aim to model the increase of cell number over time and the timing and intensity of waves of cell division. We first describe general features of the simulations before specifying to two distinct versions we have used. Briefly, we track a time-evolving population of $N(t)$ cells, consisting of $N_d(t)$ dividing cells, $N_a(t)$ arrested cells, and $N_{\mathrm{SOP}}(t)$ non-proliferative sensory organ precursors (SOPs). Each dividing cell is assigned a cell cycle time τ, stochastically selected from a specified distribution $P(\tau)$. At the end of the cell cycle time, a dividing cell divides to give rise to two daughter cells. After division, the type of daughter cells is determined stochastically according to a time-changing probability.

In practice the cell cycle time distribution $P(\tau)$, with mean μ and standard deviation σ, is chosen to be bivariate normal,

$$P_{\mathrm{bvn.}}(\tau_1, \tau_2) = \frac{1}{2\pi\sigma^2\sqrt{1-\rho_s^2}} \exp\left(-\frac{z}{2(1-\rho_s^2)}\right), \qquad [11]$$

where

$$z \equiv \frac{(\tau_1-\mu)^2+(\tau_2-\mu)^2-2\rho_{\rm s}(\tau_1-\mu)(\tau_2-\mu)}{\sigma^2} \ , \qquad [12]$$

and $\rho_{\rm s}$ is the correlation coefficient, such that correlated cycle times τ_1 and τ_2 are selected in pairs for sister cells (see Figure 6F, S6C). The mean μ and standard deviation σ of the cycle time distribution may vary in time, as described further below. The correlation coefficients between sister cells is set to the measured experimental average between wild-types, $\rho_{\rm s} = 0.55$ (Figure 6F).

A simulation timestep consists of incrementing each cell's age a and then handling any resulting divisions events. Cells divide when their age exceeds their cycle time τ_i, i.e.

$$\text{cell } i \text{ divides if } a_i > \tau_i \ . \qquad [13]$$

The default behaviour is to create a pair of daughter cells that will each divide in turn. In Figure S6E, we show the cell division rate for a simple simulation where all cells are dividing (no SOP or arrested cells), cell cycle times of sister cells are taken from the probability distribution $P_{\rm bvn.}(\tau_1,\tau_2)$ with $\mu = 4$h, $\sigma/\mu = 0.2$ and ρ_s=0.55, and all cells are at age 0 at the beginning of the simulation.

In simulations shown in other panels, a division event can lead to the generation of zero, one or two arrested cells, with respective probabilities p_0, p_1, p_2, that change in time according to:

$$p_0(t) = 1 - p(t) \qquad [14]$$

$$p_1(t) = p(t)(1-\alpha(t)) \qquad [15]$$

$$p_2(t) = p(t)\alpha(t) \ . \qquad [16]$$

The time changing variables p and α are set to be Hill-functions:

$$p(t) = \frac{\left(\frac{t}{s_p}\right)^{h_p}}{1+\left(\frac{t}{s_p}\right)^{h_p}}, \quad \alpha(t) = \frac{\left(\frac{t}{s_\alpha}\right)^{h_\alpha}}{1+\left(\frac{t}{s_\alpha}\right)^{h_\alpha}} \ , \qquad [17]$$

with switch times s_p, s_α and Hill coefficients h_p, h_α fitted to experimental data as in Figures 6K, L and reported in Table S2. Before 14.7 hAPF the probabilities p and α are set to 0.

Creation of SOP cells is handled simply by setting a window of simulation time t during which there is a fixed probability that a newly-born cell which is not arrested becomes a SOP:

$$\text{SOP probability } = \begin{cases} p_{\text{SOP}} & \text{if } t_{\text{SOP-on}} < t < t_{\text{SOP-off}} \\ 0 & \text{otherwise .} \end{cases} \qquad [18]$$

In the experiments, "SOP" cells, once specified, continue to proliferate but do so in a specialised manner: they undergo one in-plane division followed by one or two out-of-plane divisions.. Therefore, for the purpose of our analysis and simulation, they are treated as non-dividing cells once they have been created. The fitted simulation parameter p_{SOP} encodes the probability per newly-created cell that the cell is an SOP, conditioned to the cell not being arrested.

We now describe the details of simulations. Where relevant we will give parameters for the base case simulation. Parameters are either determined by experimental data, or are free parameters that are chosen to produce simulations results which match experimental data visually. Modifications from the base case described below, are described in the captions of Figure S7.

The full simulation begins at 0 hAPF and connects the available observations pre-16 hAPF to those in the main movies post-16 hAPF. Here for simplicity we neglect the possibility of cell delamination. We separate the simulation into two phases.

2.1. **First phase: from 0hAPF to 14.7hAPF.** The simulation begins with $N_0 = 18$ cells at 0 hAPF (average number of cells measured in the anterior nest at this time, Figure 7C). In the first phase, we can compare simulation results to measurements of anterior nest cell number, taken from fixed animals at this stage (Figure 7C).

During this phase, cell cycle times could be measured in live movies taken in the first ~ 3 hAPF (Figure 7B). Therefore the distribution of cycle times in the first 3.3 hAPF of simulations is set to match available cell cycle measurements taken at this stage: $\mu_0 = 2.67$ h, and the coefficient of variation $\text{CV}_0 \equiv \sigma_0/\mu_0 = 0.24$, where σ_0 is the cycle time standard deviation (determined parameters from experimental data in Figure 7B). The mean time-to-division from 0 hAPF for the first set of divisions was measured to be on average ~ 1.1

h shorter than μ_0. We mimic this in simulation by shifting the first round of divisions forward by ~ 1.1 h, so that the mean time-to-division from 0 hAPF is $[ttd]_0 = 1.58$ h.

For times $t_{\text{trans}} = 3.3$hAPF $\lesssim t \leq 14.7$ hAPF, no experimentally-measured cycle times are available. Measurements of anterior nest cell numbers (Figure 7C) suggest a pause, or at least a dramatic reduction, in cell divisions at around 12 hAPF. We therefore introduce a pause in divisions in the simulation at $t_{\text{pause-on}} = 12.5$ h (chosen parameter).

In between 3.3hAPF and 12.5hAPF, we chose for simplicity a constant mean cell cycle time. Keeping the early, ≤ 3.3hAPF value of mean cell cycle time μ_0 does not account for the measured number of cells in the anterior nest (Figure S7A). Instead we therefore set the mean cell cycle time to $\mu_1 = 4.6$h, chosen to roughly match the measured cycle times at the start of the second phase (see Figure 7B). This number leads to predicted increase in cell numbers in agreement with experiments (see Figure 7C and compare with Figure S7A). The coefficient of variation in this stage is also a free parameter, which we chose to be $\text{CV}_1 = \sigma_1/\mu_1 = 0.32$, large enough to avoid oscillatory peaks stronger than experimentally measured after 16hAPF (chosen parameter, see Figure S7C-E). As illustrated in Figure S7B and the associated caption, the pair of parameters μ_1 and t_{trans} are reasonably well constrained considering that no cycle time measurements are available for direct comparison. Indeed, t_{trans} cannot decrease by ~ 1 h because it would contradict the early cycle time measurements. On the other hand, increasing t_{trans} by ~ 1 h would require to increase the cell cycle time μ_1 to compensate for the excess number of cells created, and we find that the cell number measurement is then not matched as well as with our choice of parameters (Figure S7B). Of course, we cannot rule out that the cycle time during the phase 3.3hAPF $\leq t \leq 14.7$ hAPF changes in some more complex manner, but the simple choices made here represent a parsimonious explanation for the measured data.

In between 12.5hAPF and 14.7hAPF, the increase in the number of cells stalls significantly (Figure 7C). This is followed by a sharp increase in the number of cells around 16hAPF, which we attribute to a burst in cell division. Here we assume that the preceding pause in anterior nest cell number increase is due to inhibition of cell division, while cell ages are still increasing. Therefore in simulations when the pause is released

there is a sudden increase in cell number as a sub-population of cells that have exceeded their cycle time during the pause all divide at once. The population of cells that experiences this sudden division is effectively 're-synchronised' in terms of birth times, which has a sharpening effect on the division rate peaks in the main phase (compare Figure S7F-H in which a different implementation of the pause, which does not result in such re-synchronisation, was used). Therefore, as well as matching the cell number measurements of Figure 7C, another consideration when choosing the pause period was to attain comparable peak sharpness as shown in Figure 7F (see Figure S7I-K for the outcome with a delay in the pause). The only other free parameter with an effect on this sharpness, CV_1, has a far weaker role, because it only affects the decorrelation of division times prior to the subsequent re-synchronisation caused by the pause period.

Although we do not have live experimental data in between ~ 3.3hAPF and 14.7hAPF, it seems reasonable to describe the pause in cell number increase occuring around 12hAPF (Figure 7C) as the end of the 'cleavage' divisions characteristic of the early stages, and the subsequent cell number increase around $14-16$hAPF as the beginning of the 'expansion' divisions characteristic of the main experimental window.

2.2. **Second phase: from 14.7hAPF to 40hAPF.** At 14.7hAPF, a subset of $n_{\text{sub.}} = 95$ cells is selected to represent the noborder ROI of the movies. The mean cycle time $\mu_2(t)$ is hereafter set to track the experimental data as shown in Figure 7B (determined parameter, a 3rd-order polynomial fit to the data up to 28 hAPF and a constant value afterwards), while the coefficient of variation is fixed at $CV_2 = 0.2$ (determined parameter), a representative CV over all time and over all movies. The Hill functions for $p(t)$ and $\alpha(t)$ have switch times of $s_\alpha = 24.7$ hAPF, $s_p = 25.7$ hAPF and Hill coefficients $h_p = h_\alpha = 34$, found by manual fitting of the collected time-aligned experimental data in Figures 6K, L.

During this phase, SOPs are generated with a probability of $p_{\text{SOP}} = 0.09$ per new cell between $t_{\text{SOP-on}} = 20.6$ and $t_{\text{SOP-off}} = 24$ hAPF, conditioned on the cell not being arrested (chosen parameters).

The full simulation is not compared on a movie-by-movie basis to the post-16 hAPF experiments. Rather we create a ribbon from the mean and SD of the experimental

cell number data, for the comparison in Figure 7E, and aim for a simulation which goes roughly through the middle of the ribbon. Creating the experimental ribbon is subtle, because of arbitrary differences in the size of the noborder ROI across experiments (the noborder ROI is not biologically meaningful, as it simply comprises lineages not affected by contact with the boundary of the field-of-view). We normalise each movie's noborder cell number as $N_{\mathrm{dl}}(t) = N(t)/N_f$, where $N(t)$ is the total number of cells and N_f is the cell number of that movie evaluated in the 'first common frame' – the earliest frame that exists in all movies once the movies have been time-aligned. The dimensionless cell number $N_{\mathrm{dl}}(t)$ is used to calculate a time-dependent mean $\bar{N}_{\mathrm{dl}}(t)$ and associated SD across the movies. This mean and SD are finally remultiplied by $\bar{N}_f$, the average of N_f across movies, to give a representative ribbon of cell number as shown in Figure 7E. The SOP cell number and arrested cell number are treated in the same way.

We perform 10 runs of the simulation with different random seeds. These lead to the simulation mean and SD shown in Figures 7C and 7E. The simulation division rate in Figure 7F is one representative run, showing that the amplitude and period of oscillations in cell division rate are comparable to experiment.

3. Active elastic deformation model for annular ablations

In this section we describe an active elastic model for the tissue deformation following laser ablation. The tissue is described as an elastic material, connected to an external material by elastic links, and subjected to active anisotropic tension. We solve the model numerically using a finite element method and compare solutions to experimentally measured deformations. This is done by segmenting cell centers and cell shapes before and after annular ablation to obtain the deformation field of the cut circular piece of tissue, and comparing it to model predictions. We also take into account the deformation of the outer boundary of the cut, measured along two orthogonal axis.

3.1. **Elastic model for the constriction of the excised disc.** We consider a 2D linear-elastic material, with shear and bulk moduli K and $\bar{K}$ respectively, subjected to a homogeneous, possibly anisotropic, active tension and adhering to a substrate with elastic bonds of elasticity k. We denote $\mathbf{u}$ the displacement field, t_{ij} the tension tensor with latin

indices referring to x, y cartesian coordinates. The force balance then reads

$$\partial_i t_{ij} - k u_j = 0 \ , \qquad [19]$$

and the constitutive equation for the total tension tensor reads

$$t_{ij} = 2K\tilde{u}_{ij} + \bar{K} u_{kk}\delta_{ij} + t_{ij}^{\mathrm{act}}, \qquad [20]$$

with t_{ij}^{act} the active tension tensor, $u_{ij} = \frac{1}{2}(\partial_j u_i + \partial_i u_j)$ is the strain tensor, $\tilde{u}_{ij} = \left(u_{ij} - \frac{1}{2}u_{kk}\delta_{ij}\right)$ its anisotropic shear component, and u_{kk} the isotropic shear. We consider here $t_{ij}^{\mathrm{act}} = \zeta_x \delta_{ix}\delta_{jx} + \zeta_y \delta_{iy}\delta_{jy}$ with ζ_x and ζ_y the principal components of the active tension. We assume that the tissue tension after laser ablation is the sum of the pre-existing uniform tension tensor t_{ij}^{act} (identified here with the active tension, in an active elastic description of the material) and additional elastic stresses arising from deformation following laser ablation.

Combining the force balance equation [19] and the constitutive equation [20] results in the following PDE in terms of the components of the displacement $u_i(x, y)$:

$$\partial_i \left[2K\tilde{u}_{ij} + \bar{K} u_{kk}\delta_{ij} + t_{ij}^{\mathrm{act}}\right] - k u_j = 0, \qquad [21]$$

For a patch of material, Eq. [21] is supplemented with the traction-free boundary condition

$$t_{ij} n_i = \left[2K\tilde{u}_{ij} + \bar{K} u_{kk}\delta_{ij} + t_{ij}^{\mathrm{act}}\right] n_i = 0, \quad \text{at } \partial\Omega, \qquad [22]$$

where n_i is the outer normal to the domain Ω at the boundary $\partial\Omega$.

Before proceeding further we note that if the active tension is uniform ($t_{ij} = \zeta\delta_{ij}$) and the elastic external resistance vanishes ($k = 0$), the force balance equation [21] together with the boundary condition [22] is solved in polar coordinates (r, θ) by the radial displacement field:

$$u_r = -\frac{\zeta}{2\bar{K}} r \ , \qquad [23]$$

corresponding to a uniform isotropic shear, $u_{kk} = \partial_r u_r + \frac{u_r}{r} = -\frac{\zeta}{\bar{K}}$. As excised discs area contraction occurs non-uniformly at early developmental times (see deformation field at 16hAPF in Figure 3C), we conclude that these simplifying hypothesis do not apply in practice.

3.2. **Numerical resolution of the equations using a finite element method.** To solve Eq. [21]-[22] numerically we employ a finite element method (FEM). In the finite element method, the weak form of Eq. [21] is obtained by multiplying Eq. [21] by an arbitrary test function $w(x,y)$ and integrating over Ω

$$\begin{aligned} 0 &= \int_\Omega \left\{ \partial_i \left[2K \left(u_{ij} - \frac{1}{2} u_{kk}\delta_{ij} \right) + \bar{K} u_{kk}\delta_{ij} + t^{\text{act}}_{ij} \right] - k u_j \right\} w dx dy \\ &= \int_\Omega \left\{ -(\partial_i w) \left[2K \left(u_{ij} - \frac{1}{2} u_{kk}\delta_{ij} \right) + \bar{K} u_{kk}\delta_{ij} + t^{\text{act}}_{ij} \right] - k u_j w \right\} dx dy, \end{aligned} \quad [24]$$

where to get to the last line we have integrated by parts the term in the square brackets, and used the divergence theorem and Eq. [22] to eliminate the term in the boundary. This is the weak form of Eqs. [21] and [22]. We now consider a discretisation of our domain Ω into n_t triangles Ω_t with n vertices. We discretise the displacement as

$$u_i^h(x,y) = \sum_{a=1}^{n} u_i^a N^a(x,y), \quad [25]$$

where the superscript ^{h} indicates that the solution is approximate, a is an index denoting the label of a vertex in the discretisation and $N^a(x,y)$ is its basis function; here we employ linear interpolants, also known as tent functions. In a Galerkin FEM, the set of basis functions $N^a(x,y)$ is also employed as a set of weight functions. The discrete version of Eq. [24] becomes

$$\begin{aligned} &\sum_{b=1}^{n} \int_\Omega \left[-\partial_i N^a \left(K \left(u_j^b \partial_i N^b + u_i^b \partial_j N^b - u_k^b \delta_{ij} \partial_k N^b \right) \right.\right. \\ &\qquad \left.\left. + \bar{K} u_k^b \delta_{ij} \partial_k N^b \right) - k N^a N^b u_j^b \right] dx dy = \int_\Omega t^{\text{act}}_{ij} \partial_i N^a dx dy \\ &\sum_{b=1}^{n} \left[\int_\Omega \left[\partial_i N^a \left(K \left(\delta_{jk} \partial_i N^b + \delta_{ik} \partial_j N^b - \delta_{ij} \partial_k N^b \right) + \bar{K} \delta_{ij} \partial_k N^b \right) \right.\right. \\ &\qquad \left.\left. + k N^a N^b \delta_{jk} \right] dx dy \right] u_k^b = - \int_\Omega t^{\text{act}}_{ij} \partial_i N^a dx dy, \end{aligned} \quad [26]$$

which we can write as a linear system of equations

$$\left(K \mathsf{A}^K + \bar{K} \mathsf{A}^{\bar{K}} + k \mathsf{A}^k \right) \mathsf{u} = \zeta_x \mathsf{B}^x + \zeta_y \mathsf{B}^y. \quad [27]$$

Here

$$\mathsf{u} = \begin{pmatrix} u_x^1 \\ u_y^1 \\ \vdots \\ u_x^n \\ u_y^n \end{pmatrix}, \tag{28}$$

is the vector of unknowns,

$$\mathsf{B}^x = \begin{pmatrix} -\int_\Omega \partial_x N^1 dxdy \\ 0 \\ \vdots \\ -\int_\Omega \partial_x N^n dxdy \\ 0 \end{pmatrix}, \qquad \mathsf{B}^y = \begin{pmatrix} 0 \\ -\int_\Omega \partial_y N^1 dxdy \\ \vdots \\ 0 \\ -\int_\Omega \partial_y N^n dxdy \end{pmatrix}, \tag{29}$$

are the vectors in the right-hand side, and the matrices have the form

$$\mathsf{A}^X = \begin{pmatrix} A^X_{1,x,1,x} & A^X_{1,x,1,y} & A^X_{1,x,2,x} & A^X_{1,x,2,y} & \cdots & A^X_{1,x,n,x} & A^X_{1,x,n,y} \\ A^X_{1,y,1,x} & A^X_{1,y,1,y} & A^X_{1,y,2,x} & A^X_{1,y,2,y} & \cdots & A^X_{1,y,n,x} & A^X_{1,y,n,y} \\ A^X_{2,x,1,x} & A^X_{2,x,1,y} & A^X_{2,x,2,x} & A^X_{2,x,2,y} & \cdots & A^X_{2,x,n,x} & A^X_{2,x,n,y} \\ A^X_{2,y,1,x} & A^X_{2,y,1,y} & A^X_{2,y,2,x} & A^X_{2,y,2,y} & \cdots & A^X_{2,y,n,x} & A^X_{2,y,n,y} \\ \vdots & \vdots & \vdots & \vdots & \ddots & \vdots & \vdots \\ A^X_{n,x,1,x} & A^X_{n,x,1,y} & A^X_{n,x,2,x} & A^X_{n,x,2,y} & \cdots & A^X_{n,x,n,x} & A^X_{n,x,n,y} \\ A^X_{n,y,1,x} & A^X_{n,y,1,y} & A^X_{n,y,2,x} & A^X_{n,y,2,y} & \cdots & A^X_{n,y,n,x} & A^X_{n,y,n,y} \end{pmatrix}, \tag{30}$$

for $X = K, \bar{K}, k$, with components

$$\begin{aligned} \mathsf{A}^K_{a,j,b,k} &= \int_\Omega \left[(\partial_i N^a) \left(\partial_i N^b\right) \delta_{jk} + (\partial_k N^a) \left(\partial_j N^b\right) - (\partial_j N^a) \left(\partial_k N^b\right) \right] dxdy, \\ \mathsf{A}^{\bar{K}}_{a,j,b,k} &= \int_\Omega (\partial_j N^a) \left(\partial_k N^b\right) dxdy, \\ \mathsf{A}^k_{a,j,b,k} &= \int_\Omega N^a N^b dxdy \delta_{jk}. \end{aligned} \tag{31}$$

To compute these integrals, we partition the domain Ω into the triangles Ω_t and use Gaussian integration to approximate the integral in each Ω_t,

$$\int_\Omega f(x,y)dxdy = \sum_{t=1}^{n_t} \int_{\Omega_t} f(x,y)dxdy = \sum_{t=1}^{n_t}\sum_{g=1}^{n_g} w_g f(x_g^t, y_g^t) J^t \ , \qquad [32]$$

where w_g are the Gaussian integration weights, (x_g^t, y_g^t) the coordinates of the Gauss points in triangle t, and J^t the triangle area.

3.3. **Least-squares procedure for parameter fitting from annular ablation experiments.** From annular ablation experiments, we can extract (1) cell-centre displacements $u_i(x_c, y_c)$, (2) relative cell area change $\lambda(x_c, y_c)$ and (3) cell elongation change in the form of a nematic tensor $Q_{ij}(x_c, y_c)$, where (x_c, y_c) represent the cell centre right before ablation. For the cell centre displacements, we remove the center-of-mass motion of the inner disc. We would then like to use the FEM solution to fit the parameters $\boldsymbol{p} = (K/\bar{K}, \zeta_x/\bar{K}, \zeta_y/\bar{K}, k/\bar{K})$; note that these parameters are normalised by $\bar{K}$ and form a set of independent parameters in Eq. [21]. We first define the function

$$\begin{aligned}\mathcal{L}(\boldsymbol{p}; u, \lambda, Q_{ij}) = \frac{1}{2}\sum_c \Big[& k_u \left|u_i^{cc}(x_c, y_c) - u_i^h(x_c, y_c; \boldsymbol{p})\right|^2 \\ & + k_\lambda \left(\lambda(x_c, y_c) - \partial_i u_i^h((x_c, y_c; \boldsymbol{p})\right)^2 \\ & + k_Q \left|Q_{ij}(x_c, y_c) - \tilde{u}_{ij}^h(x_c, y_c; \boldsymbol{p})\right|^2 \Big] \ ,\end{aligned} \qquad [33]$$

where k_u, k_λ and k_Q are penalty parameters (for the fits in this article we have used $k_u = 1/R^2$ with R the radius of the disc before deformation, $k_\lambda = k_Q = 1$ although we checked that the fits are relatively insensitive to variations of the relative weight of the displacement and shear penalty parameters within a range $k_u/k_\lambda = 0 \leftrightarrow 20/R^2$). This function penalises deviations of the finite element solution $u^h(x,y)$ obtained for the set of parameters $\boldsymbol{p}$ from experimental data. To fit experimental data and obtain the model parameters $\boldsymbol{p}$, we then minimise

$$\boldsymbol{p} = \underset{\arg\min}{\boldsymbol{p}'} \ \mathcal{L}(\boldsymbol{p}'; u, \lambda, Q_{ij}), \qquad [34]$$

for which we use a L-BFGS algorithm as implemented in the function `minimize` of the python `Scipy` package. We compute gradients of $\mathcal{L}$ as

$$\frac{\partial \mathcal{L}}{\partial \boldsymbol{p}} = \sum_c \left[k_u \left(u_i^{cc}(x_c, y_c) - u_i^h(x_c, y_c; \boldsymbol{p}) \right) \frac{\partial u_i^h}{\partial \boldsymbol{p}} \right. \\ + k_\lambda \left(\lambda(x_c, y_c) - \partial_i u_i^h((x_c, y_c; \boldsymbol{p}) \right) \frac{\partial(\partial_i u_i^h)}{\partial \boldsymbol{p}} \\ \left. + k_Q \left(Q_{ij}(x_c, y_c) - \tilde{u}_{ij}^h(x_c, y_c; \boldsymbol{p}) \right) \frac{\partial \tilde{u}_{ij}^h}{\partial \boldsymbol{p}} \right]. \quad [35]$$

To compute $\frac{\partial u_i^h}{\partial \boldsymbol{p}}, \frac{\partial \left(\partial_i u_i^h\right)}{\partial \boldsymbol{p}}, \frac{\partial \tilde{u}_{ij}^h}{\partial \boldsymbol{p}}$, we note that

$$\frac{\partial u_i^h}{\partial \boldsymbol{p}} = \sum_{a=1}^n N^a \frac{\partial u_i^a}{\partial \boldsymbol{p}}, \quad \frac{\partial \left(\partial_i u_i^h\right)}{\partial \boldsymbol{p}} = \sum_{a=1}^n (\partial_i N^a) \frac{\partial u_i^a}{\partial \boldsymbol{p}}, \\ \frac{\partial \tilde{u}_{ij}^h}{\partial \boldsymbol{p}} = \frac{1}{2} \sum_{a=1}^n \left[\partial_j N^a \delta_{ik} + \partial_i N^a \delta_{jk} - \partial_k N^a \delta_{ij} \right] \frac{\partial u_k^a}{\partial \boldsymbol{p}}, \quad [36]$$

and $\frac{\partial u_i^a}{\partial \boldsymbol{p}}$ are the components of $\frac{\partial \mathsf{u}}{\partial \boldsymbol{p}}$. To calculate these variations, we note that for instance varying K by δK, the solution $\mathsf{u} + \delta \mathsf{u}$ satisfies

$$\left((K + \delta K) \mathsf{A}^K + \bar{K} \mathsf{A}^{\bar{K}} + k \mathsf{A}^k \right) (\mathsf{u} + \delta \mathsf{u}) = \zeta_x \mathsf{B}^x + \zeta_y \mathsf{B}^y, \quad [37]$$

and since u is a solution of the original problem, we have up to first order terms

$$\left(K \mathsf{A}^K + \bar{K} \mathsf{A}^{\bar{K}} + k \mathsf{A}^k \right) \delta \mathsf{u} = -\delta K \mathsf{A}^K \mathsf{u}, \quad [38]$$

so

$$\left(K \mathsf{A}^K + \bar{K} \mathsf{A}^{\bar{K}} + k \mathsf{A}^k \right) \frac{\partial \mathsf{u}}{\partial K} = -\mathsf{A}^K \mathsf{u}. \quad [39]$$

Equivalently, one finds that

$$\left(K \mathsf{A}^K + \bar{K} \mathsf{A}^{\bar{K}} + k \mathsf{A}^k \right) \frac{\partial \mathsf{u}}{\partial k} = -\mathsf{A}^k \mathsf{u}, \\ \left(K \mathsf{A}^K + \bar{K} \mathsf{A}^{\bar{K}} + k \mathsf{A}^k \right) \frac{\partial \mathsf{u}}{\partial \zeta_x} = \mathsf{B}^x, \\ \left(K \mathsf{A}^K + \bar{K} \mathsf{A}^{\bar{K}} + k \mathsf{A}^k \right) \frac{\partial \mathsf{u}}{\partial \zeta_y} = \mathsf{B}^y. \quad [40]$$

Note that variations with respect to the normalised parameters $(K/\bar{K}, \zeta_x/\bar{K}, \zeta_y/\bar{K}, k/\bar{K})$ can be directly obtained from the variations with respect to the non-normalised parameters (K, ζ_x, ζ_y), e.g. $\frac{\partial \mathsf{u}}{\partial(K/\bar{K})} = \bar{K}\frac{\partial \mathsf{u}}{\partial K}$. In practice, we perform a single fit including all times and mutants and minimise the function $\mathcal{L} = \sum_t \sum_M \mathcal{L}_t^{\mathrm{M}}$ where t =16 hAPF, 21 hAPF, 26 hAPF, 31 hAPF, and M = WT, MMP and TIMP, with the restriction that $K/\bar{K}$ is constant over time and across mutants, and data for TIMP are only for t=16 and 26 hAPF.

Together with cell centre displacements, relative cell area change and cell elongation change in the inner patch obtained from our segmented movies, we also included the displacement of the X and Y axes of the outer ablation contour, which are treated as a cell centre displacement in the data (but without an associated changes in cell area and cell elongation). To get a measure of variability of the fit, we performed a bootstrapping analysis: once we got the best fit for the data, we used the list of residuals (the errors in displacement, relative cell area changes and cell elongation change for each cell) to produce new, synthetic samples by assigning to each cell the displacement, relative cell area change and cell elongation change predicted by the fit plus one of the residuals chosen from the list of residuals with replacement. The fit was then repeated for each of these synthetic samples to obtain a distribution for the fitting parameters, which we report in box plots for the different mutants in Figures 3F-H, Figures S3D-F, 4J-M, S4K-L. We produced 1000 of these synthetic samples.

We show simulations results using the best fit parameters for the different conditions in Figures 3E and S4J. Results from experiments (top) and simulations (bottom) are shown on the computational mesh for comparison. We use a colormap to represent the isotropic shear u_{ii}, with linear interpolation on each triangle of the mesh. We use lines to represent the anisotropic shear $\tilde{u}_{ij}$, plotted at each node of the mesh. To plot these lines, we note that, since it is a nematic tensor, the anisotropic shear $\tilde{u}_{ij}$ can be written as $S(n_i n_j - \delta_{ij}/2)$ for some S and $\boldsymbol{n}$, which represent the magnitude (S) and orientation ($\boldsymbol{n}$) of the shear. We then find S and $\boldsymbol{n}$ and plot the vector $S\boldsymbol{n}$ as a line. To obtain the experimental values for isotropic and anisotropic shear in the computational mesh, we interpolate the results in the nodes of the mesh using Gaussian kernels centred at the

nodes with a width of $R_g = 3\mu m$, which is also used as a cutoff; each experimental point then contributes with a Gaussian weight to mesh nodes located within a radius R_g and these weights are normalised to add up to 1 for each node.

METHOD S1 REFERENCES

S1. Tetley, R.J., Staddon, M.F., Heller, D., Hoppe, A., Banerjee, S., and Mao, Y. (2019). Tissue Fluidity Promotes Epithelial Wound Healing. Nat Phys *15*, 1195-1203. 10.1038/s41567-019-0618-1.

S2. Çiçek, Ö., Abdulkadir, A., Lienkamp, S.S., Brox, T., and Ronneberger, O. (2016). 3D U-Net: Learning Dense Volumetric Segmentation from Sparse Annotation. In Medical Image Computing and Computer-Assisted Intervention – MICCAI 2016, pp. 424-432. 10.1007/978-3-319-46723-8_49.

S3. Heller, D., Hoppe, A., Restrepo, S., Gatti, L., Tournier, A.L., Tapon, N., Basler, K., and Mao, Y. (2016). EpiTools: An Open-Source Image Analysis Toolkit for Quantifying Epithelial Growth Dynamics. Dev Cell *36*, 103-116. 10.1016/j.devcel.2015.12.012.

S4. Aigouy, B., Farhadifar, R., Staple, D.B., Sagner, A., Roper, J.C., Julicher, F., and Eaton, S. (2010). Cell flow reorients the axis of planar polarity in the wing epithelium of Drosophila. Cell *142*, 773-786. 10.1016/j.cell.2010.07.042.

S5. Etournay, R., Merkel, M., Popovic, M., Brandl, H., Dye, N.A., Aigouy, B., Salbreux, G., Eaton, S., and Julicher, F. (2016). TissueMiner: A multiscale analysis toolkit to quantify how cellular processes create tissue dynamics. Elife *5*. 10.7554/eLife.14334.

S6. Etournay, R., Popovic, M., Merkel, M., Nandi, A., Blasse, C., Aigouy, B., Brandl, H., Myers, G., Salbreux, G., Julicher, F., and Eaton, S. (2015). Interplay of cell dynamics and epithelial tension during morphogenesis of the Drosophila pupal wing. Elife *4*, e07090. 10.7554/eLife.07090.

S7. Fabre, C.C., Casal, J., and Lawrence, P.A. (2008). The abdomen of Drosophila: does planar cell polarity orient the neurons of mechanosensory bristles? Neural Dev *3*, 12. 10.1186/1749-8104-3-12.
