# Supplementary material for: ECM degradation in the Drosophila abdominal epidermis initiates tissue growth that ceases with rapid cell-cycle exit
Source: Curr Biol. 2022 Mar 28;32(6):1285–1300.e4. doi: 10.1016/j.cub.2022.01.045 (PMC8967408; doi:10.1016/j.cub.2022.01.045)
Supplement: Document S2. Article plus supplemental information [file mmc10.pdf]

Article

Current Biology

ECM degradation in the *Drosophila* abdominal epidermis initiates tissue growth that ceases with rapid cell-cycle exit

Highlights

- Growth of the fly abdominal epidermis is triggered by ECM degradation
- Abdominal progenitors exhibit an oscillatory proliferation rate during expansion
- Tissue tension does not decrease as growth terminates
- Developmental growth termination occurs by a rapid transition to cell-cycle exit

Authors

John Robert Davis, Anna P. Ainslie, John J. Williamson, ..., Enrique Martin-Blanco, Guillaume Salbreux, Nicolas Tapon

Correspondence

guillaume.salbreux@unige.ch (G.S.), nic.tapon@crick.ac.uk (N.T.)

In brief

Davis et al. use live imaging and computational methods to quantitatively analyze developmental growth in the *Drosophila* adult abdominal epidermis. Abdominal growth is initiated by the degradation of the basement membrane on which the epidermal progenitors are attached and terminated by a rapid exit from the cell cycle rather than a gradual slowdown.

Davis et al., 2022, Current Biology *32*, 1285–1300
March 28, 2022 © 2022 The Authors. Published by Elsevier Inc.
https://doi.org/10.1016/j.cub.2022.01.045

Current Biology

CellPress OPEN ACCESS

Article

ECM degradation in the *Drosophila* abdominal epidermis initiates tissue growth that ceases with rapid cell-cycle exit

John Robert Davis,[1,6] Anna P. Ainslie,[1,6] John J. Williamson,[2,6] Ana Ferreira,[1,6] Alejandro Torres-Sánchez,[2] Andreas Hoppe,[3] Federica Mangione,[1] Matthew B. Smith,[2] Enrique Martin-Blanco,[4] Guillaume Salbreux,[2,5,7,*] and Nicolas Tapon[1,7,8,9,*]

[1]Apoptosis and Proliferation Control Laboratory, The Francis Crick Institute, 1 Midland Road, London NW1 1AT, UK
[2]Theoretical Physics of Biology Laboratory, The Francis Crick Institute, 1 Midland Road, London NW1 1AT, UK
[3]Faculty of Science, Engineering and Computing, Kingston University, Kingston-upon-Thames KT1 2EE, UK
[4]Instituto de Biología Molecular de Barcelona, Consejo Superior de Investigaciones Científicas, Parc Científic de Barcelona, C/Baldiri Reixac, 4-8, Torre R, 3era Planta, 08028 Barcelona, Spain
[5]Department of Genetics and Evolution, University of Geneva, Quai Ernest Ansermet 30, 1211 Geneva, Switzerland
[6]These authors contributed equally
[7]Senior authors
[8]Twitter: @taponlab
[9]Lead contact
*Correspondence: guillaume.salbreux@unige.ch (G.S.), nic.tapon@crick.ac.uk (N.T.)
https://doi.org/10.1016/j.cub.2022.01.045

SUMMARY

During development, multicellular organisms undergo stereotypical patterns of tissue growth in space and time. How developmental growth is orchestrated remains unclear, largely due to the difficulty of observing and quantitating this process in a living organism. *Drosophila* histoblast nests are small clusters of progenitor epithelial cells that undergo extensive growth to give rise to the adult abdominal epidermis and are amenable to live imaging. Our quantitative analysis of histoblast proliferation and tissue mechanics reveals that tissue growth is driven by cell divisions initiated through basal extracellular matrix degradation by matrix metalloproteases secreted by the neighboring larval epidermal cells. Laser ablations and computational simulations show that tissue mechanical tension does not decrease as the histoblasts fill the abdominal epidermal surface. During tissue growth, the histoblasts display oscillatory cell division rates until growth termination occurs through the rapid emergence of G0/G1 arrested cells, rather than a gradual increase in cell-cycle time as observed in other systems such as the *Drosophila* wing and mouse postnatal epidermis. Different developing tissues can therefore achieve their final size using distinct growth termination strategies. Thus, adult abdominal epidermal development is characterized by changes in the tissue microenvironment and a rapid exit from the cell cycle.

INTRODUCTION

During development, tissue growth must be tightly coordinated in time and space.[1–3] However, due to the difficulty of measuring quantitative growth parameters in living organisms, we lack the ability to analyze factors that dictate developmental growth with the high level of precision achieved in expanding populations of unicellular organisms or animal cells in culture.[4,5] For instance, while much evidence links proliferation rates with cell geometry, mechanical tension, and compression,[3,6] this relationship has not been systematically examined throughout the growth of a model tissue. Likewise, embryonic development in metazoans relies on key transitions in modes of cell division, such as from rapid cleavage cycles to slower asynchronous cell cycles at the mid-blastula transition.[7] However, it has remained difficult to quantify the impact of these transitions on developmental growth in living organisms and ascertain whether the timings of these transitions are developmentally controlled or cell-intrinsic events.

The recent development of analytical tools that capture cell and tissue deformations,[8,9] combined with progress in live imaging *in vivo*,[10] have transformed our ability to derive quantitative parameters that can be used to understand and model complex developmental processes. The *Drosophila* wing disc has been extensively used as a model of tissue growth;[11] however, as its growth occurs predominantly during the larval stages when the animal is mobile, quantitation of cellular growth dynamics on intact animals is difficult (Figure 1A). *Drosophila* histoblasts are a population of precursor cells that give rise to the adult abdominal epidermis, and whose growth occur mainly during the pupal stages when the animal is sessile (Figure 1A). Histoblasts are therefore amenable to live imaging during their entire growth period and are an ideal system to exploit tools developed for analyzing tissue deformation.[12–14]

This is an open access article under the CC BY license (http://creativecommons.org/licenses/by/4.0/).

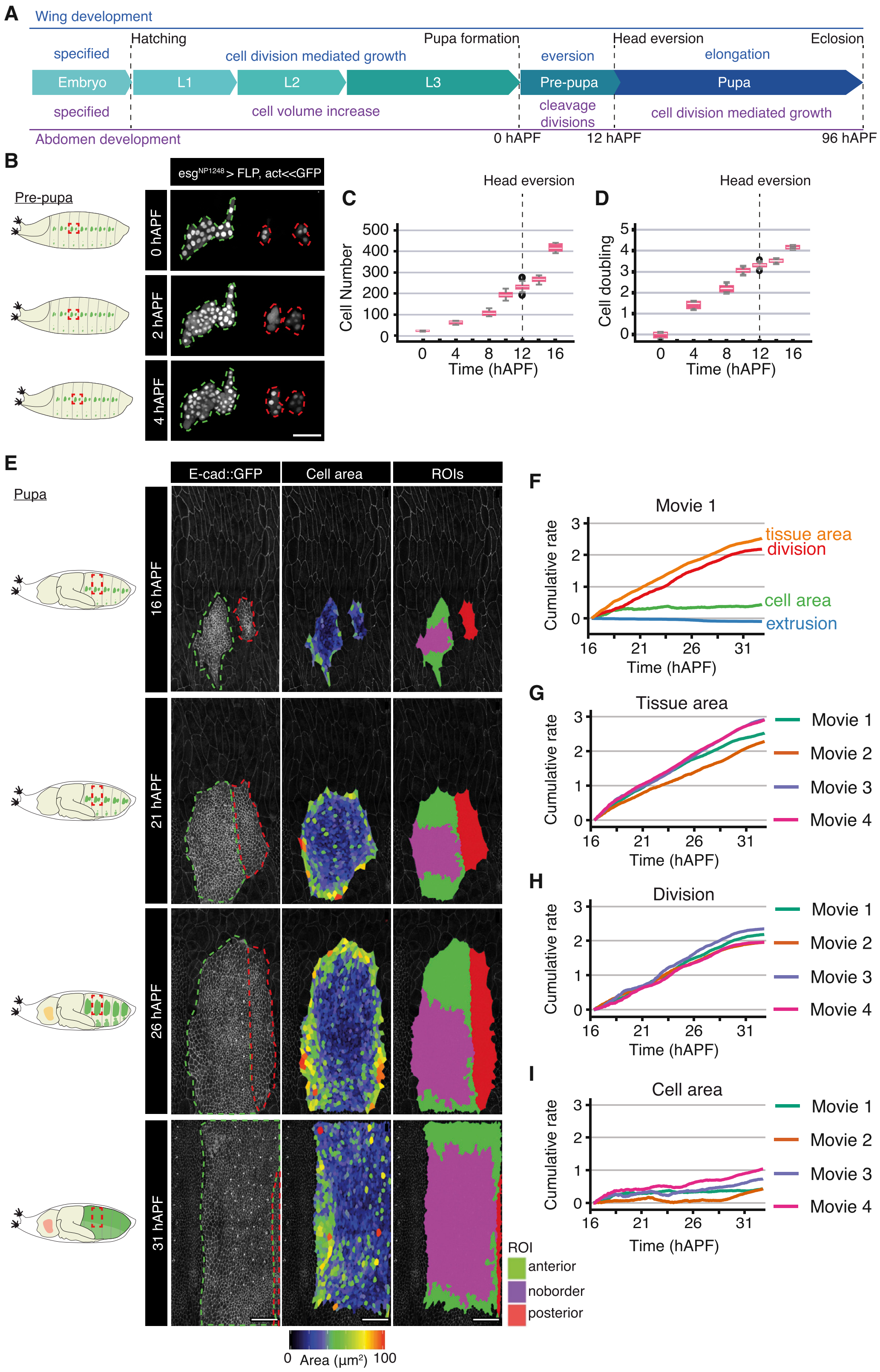

(legend on next page)

The adult *Drosophila* abdominal epidermis develops from small islands (nests) of progenitor cells called histoblasts, which are specified during embryonic development.[16,17] Each abdominal hemisegment contains four nests: two located dorso-laterally (dorsal anterior and dorsal posterior), one located ventrally, and one spiracular nest located laterally.[18,19] During the larval period, the histoblasts are quiescent (G2 arrest).[18,20,21] They are induced to enter the cell cycle by the pulse of the steroid hormone ecdysone that triggers the larval/pupal transition (0 h after puparium formation [APF]).[22] Following a period of three cleavage divisions, where cell numbers increase but total tissue volume stays constant, the histoblasts enter an expansion phase (around 14–16 hAPF) where they proliferate and grow, displacing the surrounding larval epithelial cells (LECs, large polyploid cells that formed the larval epidermis). The LECs are extruded and engulfed by circulating macrophages (hemocytes).[13,14,22–25] LEC death requires both ecdysone signaling and displacement by the expanding histoblast nests.[13,22–25] Once the histoblasts cover the entire abdominal surface, proliferation ceases and differentiation proceeds to give rise to the adult abdomen.

Here, using live imaging and cell tracking, we analyze the growth of the dorsal histoblast nests at the cellular and tissue scale to uncover how cellular behaviors give rise to tissue growth kinetics. We reveal that histoblast tissue growth is predominantly driven by cell divisions, which are initiated by remodeling of the extracellular matrix (ECM) and characterized by oscillations in proliferation rates due to a small variability in cell-cycle time. Finally, growth termination occurs through an abrupt transition to arrest without a preceding slowing down of the cell cycle and is independent of mechanical compression.

RESULTS

Measuring proliferation kinetics of the *Drosophila* dorsal abdominal epidermis identifies a pause between the cleavage and expansion phases

To obtain quantitative data on the cleavage division phase of histoblast development, we quantified the number of histoblasts from 0 hAPF onward, during the prepupal and early pupal stages (Figures 1B–1D). Before ~3 hAPF, we could follow cell division by time lapse and measure an average cell cycle time of ~2.7 h (Figure 1B; Video S1). Later, between ~4 and ~16 hAPF, continuous live imaging of histoblasts was not possible because of the extensive movements of the pupae. Therefore, we quantified the number of cells in the anterior histoblast nest in fixed images at different times between 0 hAPF up until 16 hAPF, when live imaging was feasible again (Figures 1C and 1D). This indicated that the short cell-cycle time measured before 3 hAPF was not maintained at later times but instead was slowing down (Figures 1C and 1D). Our quantification was consistent with roughly three cleavage divisions occurring up to ~10 hAPF (Figure 1D). However, cell numbers plateau between ~10 and ~14 hAPF, before undergoing a sudden rise between ~14 and ~16 hAPF. This supports previous observations[22] that proliferation occurs in two phases, a cleavage phase, followed by an expansion phase. Moreover, we have identified a pause in proliferation as histoblasts transition between the two phases.

During larval development, the histoblasts are arrested in the G2 phase of the cell cycle.[18,20,21] To determine whether histoblasts are in a specific cell-cycle phase during the proliferation pause, we used the FUCCI cell-cycle marker system (Figure S1A).[26] At 12 hAPF, only 18% of cells were in G1, while 50% were in G2/M (Figure S1C). As the cells at this time point show little or no division (Video S1; Figure 1D), we conclude that around 50% of cells are accumulated in G2. In contrast, at 16 hAPF during the expansion phase, 44% of cells are in G1, and 36% are in G2/M (Figure S1C). Thus, during the transition between the cleavage and expansion phases, the histoblasts tend to accumulate in G2 before undergoing a burst of mitoses between 14 and 16 hAPF (Figure 1C).

The dorsal histoblast nests expand through cell division

To quantitate the expansion phase of abdominal development, we imaged cell junctions with *E-cad::GFP* from 16 to ~ 33 hAPF[27] (STAR Methods; Figure 1E). The resultant four wild-type movies (designated Movies 1–4) were processed and analyzed using a custom-built pipeline to track cells (Figures 1E and S1; STAR Methods; Video S2). We then defined an anterior "no border region of interest" (noborder ROI) comprised complete lineages of anterior nest histoblasts that never contact the edge of the image frame (Figure 1E, right column; Video S2; Methods S1). The emergence of specialized cells called sensory organ precursors (SOPs) was used to temporally align four wild-type movies (Figures S1G–S1L; STAR Methods).

Figure 1. Quantitative analysis of cellular contributions to abdominal growth

(A) Schematic overview on timing of tissue growth during *Drosophila* wing and abdominal development.

(B) Left: schematic representation of pupal body plan, adapted from Hartenstein,[15] with histoblasts in green and imaging region shown in red dashed box. Right: time-lapse confocal images of histoblast nests at the times indicated during early pre-pupal stages. Histoblasts are labeled by driving *nls-GFP* expression in this tissue. Anterior and posterior nests are outlined in green and red, respectively. Note that the nests are sometimes split at the end of larval development (0 hAPF), as is the case here for the posterior nest. Scale bars, 50 μm. Dorsal is to the top; anterior is to the left. See also Video S1.

(C) Quantification of total dorsal histoblast numbers (anterior + posterior nests) from fixed images during early pupal stages. n = 15, 3, 8, 12, 13, 8, and 7 at successive time points.

(D) Cell doublings [$\log_2$(N/N0)] of the histoblasts during early pupal stages.

(E) Left: similar schematic representation as in (B). Right: left column are confocal images of histoblasts in live pupa expressing *E-cad::GFP* at the time points indicated. Anterior and posterior nests are outlined in green and red, respectively. At 16 hAPF, the anterior and posterior histoblast nests are separate and surrounded by the LECs. The nests then spread and eventually occupy the entire surface of the segment. Middle column displays heatmaps showing cell areas. Right panels are labeled regions of interest (ROI): anterior (green + magenta), posterior (red), and the "no border" ROI (magenta). Scale bars, 50 μm. See also Video S2 and Methods S1.

(F) Decomposition of the cumulative area expansion rate of the tissue into contributions from cell division, cell area change, and cell extrusion (wild-type movie 1, noborder ROI). See also Figure S1.

(G–I) Cumulative tissue area expansion rate (G) and contributions from cell division (H) and cell area (I) for four different wild-type movies. Movies are time-aligned based on the appearance of sensory organ precursor (SOP) cells (Figure S1L). See also Methods S1.

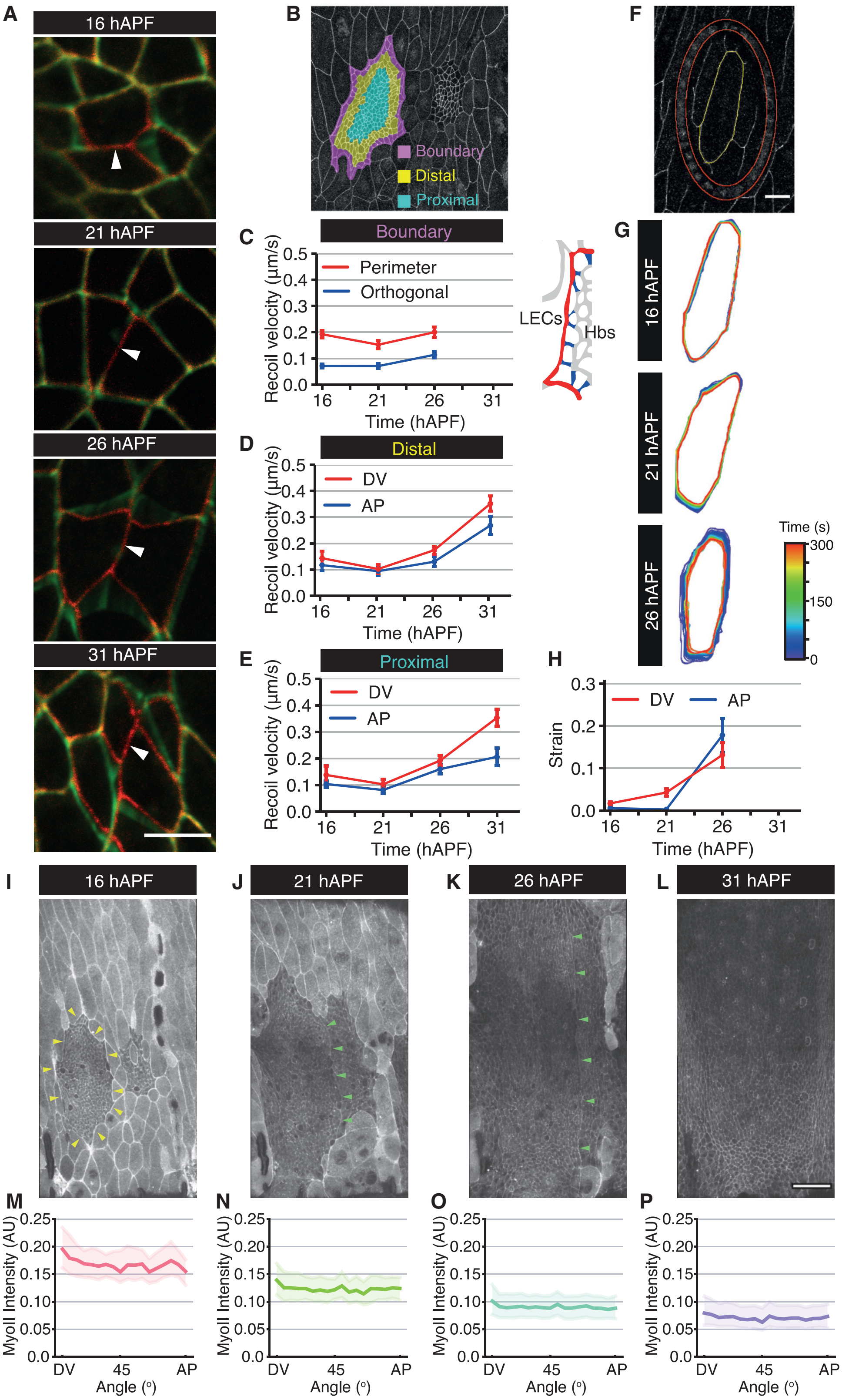

Figure 2. Junctional tension increases through development

(A) Snapshots of laser ablation experiments. Red color, before laser ablation; green color, time frames after laser ablation. Scale bars, 5 µm.

(B) Confocal image of dorsal histoblast nests at 16 hAPF where the position of boundary, distal, and proximal cells is highlighted.

(legend continued on next page)

To analyze tissue growth, we performed a shear decomposition analysis of the segmented wild-type movies using Tissue Miner.[9,28] We focused our analysis on the expansion kinetics of the histoblast nests by examining how the relative rates of change in cell area and cell number (cell division or extrusion) contribute to the area expansion rate of the tissue (Figures 1F–1I and S1N–S1Q). This quantitative analysis showed that the dorsal histoblast nests grow primarily via cell division (Figures 1F and 1H), as previously proposed.[22] The average cell area increase contributed modestly to tissue area expansion (Figures 1F and 1I). Cell loss ("extrusion"), through either cell death or delamination, had a minimal negative contribution to histoblast nest growth (Figure 1F). The cell area contribution to growth is far more variable than that of cell divisions (Figures 1H and 1I). Furthermore, cell areas in the anterior nest are also spatially variable, with cells around the periphery of the nest having a larger apical area than their counterparts in the nest center (Figure 1E, middle column). This may be due to their location at the interface with LECs, which have been shown to exert forces on the boundary histoblasts when undergoing apoptosis.[13,24] By 31 hAPF, the rate of division was minimal (Figure 1H), suggesting that the histoblasts had arrested by this stage, consistent with previous reports.[13,22] Thus, histoblast proliferation during pupal development consists of four steps: cleavage, pause, expansion, and arrest.

Tissue tension does not decrease during abdominal development

Work in cell culture and several *in vivo* systems has suggested that mechanical constraints influence proliferation; for instance, cells under tension are generally more likely to divide than cells under compression.[3,6] Indeed, mechanical compression, or a decrease in tension, have been suggested as mechanisms for developmental growth termination in model tissues such as the *Drosophila* wing imaginal disc.[3,6] We therefore wished to examine whether there were changes in tissue mechanics during abdominal development that could influence histoblast expansion and growth arrest.

To explore whether mechanical stresses were changing in expanding histoblast nests over time, we performed single-junction ablations in different regions of the anterior nest (Figures 2A and 2B). We calculated the recoil velocity of junction vertices, which is often used as a proxy for mechanical tension and found two distinct behaviors depending on the location of junctions (Figures 2A–2E and S2A). Junctions near the LEC/histoblast border showed consistent recoil velocities throughout development, with higher velocities along junctions shared with LECs (perimeter) compared with junctions that have a vertex shared with other histoblasts (orthogonal) (Figures 2C and S2A). Junctions away from the boundary showed an increased recoil after 21 hAPF, as well as a bias toward the dorsal-ventral (DV) axis (Figures 2D, 2E, and S2A). We tested whether this increase in junction recoil after ablation was associated with cell shape changes but found no clear correlation between junction length and recoil velocity at any developmental stage (Figure S2B). As LECs are contiguous with the histoblasts, we also examined whether they experienced changes in tension. Using laser ablation, we excised individual LECs and followed the subsequent LEC deformation (Figures 2F–2H and S2C–S2E; Video S3). At 16 and 21 hAPF, LECs showed minimal shape changes (Figures 2G, 2H, and S2C). At 26 hAPF, however, there was a large reduction in apical area and an increase in recoil velocity upon ablation (Figures 2G, 2H, S2C, and S2D). Furthermore, the shape contraction was slightly more pronounced along the anterior-posterior (AP) than the DV axis. This is consistent with a recently reported increase in recoil velocity in single LEC junction ablations between 20 and 27 hAPF.[25] Thus, in contrast to reports in other tissues such as the *Drosophila* wing disc,[29] we observed that recoil velocities upon ablation of histoblasts and LECs increase over developmental time.

Elevated junctional myosin II (Myo II) levels are one possible explanation for the temporal increase in recoil velocity. Examining junctional Myo II intensity (Figures 2I–2P, S2F, and S2G; Video S4) showed that the highest levels in histoblasts are at the histoblast-LEC boundary[25] (Figures 2I–2K), likely explaining the large recoil after ablation along perimeter junctions (Figure 2C). However, Myo II junctional levels globally decline throughout development, while also switching from a slight intensity bias along DV junctions at 16 hAPF to being isotropic by 31 hAPF (Figures 2M–2P). We also observed no strong dependency of Myo II intensity on junction length (Figure S2F). We conclude that the increase in recoil velocity over time is not linked to an increase in Myo II junctional levels.

Junctional tension is only one of the components generating forces within the tissue, so we wondered whether the large-scale tissue tension was changing over time in the same way as junctional recoil velocity. To test this, we performed apical annular cuts with a diameter of about 10 cells in a defined region of the anterior histoblast nests at different time points (Figure 3A; Video S3). Consistent with observations of single-junction ablations, the strain and recoil velocity measured in the AP and DV directions were increasing over time (Figures 3B and S3A–S3C; STAR Methods). In addition, the spatial pattern of cell area contraction within excised discs exhibited a striking change

(C–E) Quantification throughout development of recoil velocity for single junction ablations for cells located along the boundary (C; perimeter junctions n = 38, 31, and 29 and orthogonal junctions n = 39, 31, and 29 at successive times), or in the distal (D; DV junctions n = 16, 19, 30, and 20 and AP junctions n = 16, 9, 24, and 21) and proximal (E; DV junctions n = 16, 11, 33, 21 and AP junctions n = 21, 22, 38, and 22) positions within the nest. See also Figure S2.

(F) Snapshot of an LEC at 16 hAPF in the resting state after an annular ablation in an *E-cad::GFP*-expressing animal. Excised LECs were segmented (yellow) and their shape change analyzed to assess strain. See also Video S3 and Figure S2.

(G) Representative LEC segmentations after ablation, temporally overlaid for each developmental stage examined.

(H) Quantification of Hencky's true strain for LEC AP (short) and DV (long) axes after ablation.

(I–L) Example apical projections of confocal images from a pupa expressing *Sqh::GFP* (Myo II) and *E-cad::mKate2* at the indicated time points. Note the higher levels of Myo II intensity at the histoblast-LEC boundary (yellow arrowheads) and the Myo II supra-cellular cable at the histoblast AP nest boundary (green arrowheads).[30] Scale bars, 50 μm. See also Video S4.

(M–P) Median apical Myo II intensity along junctions as a function of binned junction angles (solid line denotes median intensity and ribbon denotes the interquartile range) at different times (as in I–L). See also Figure S2.

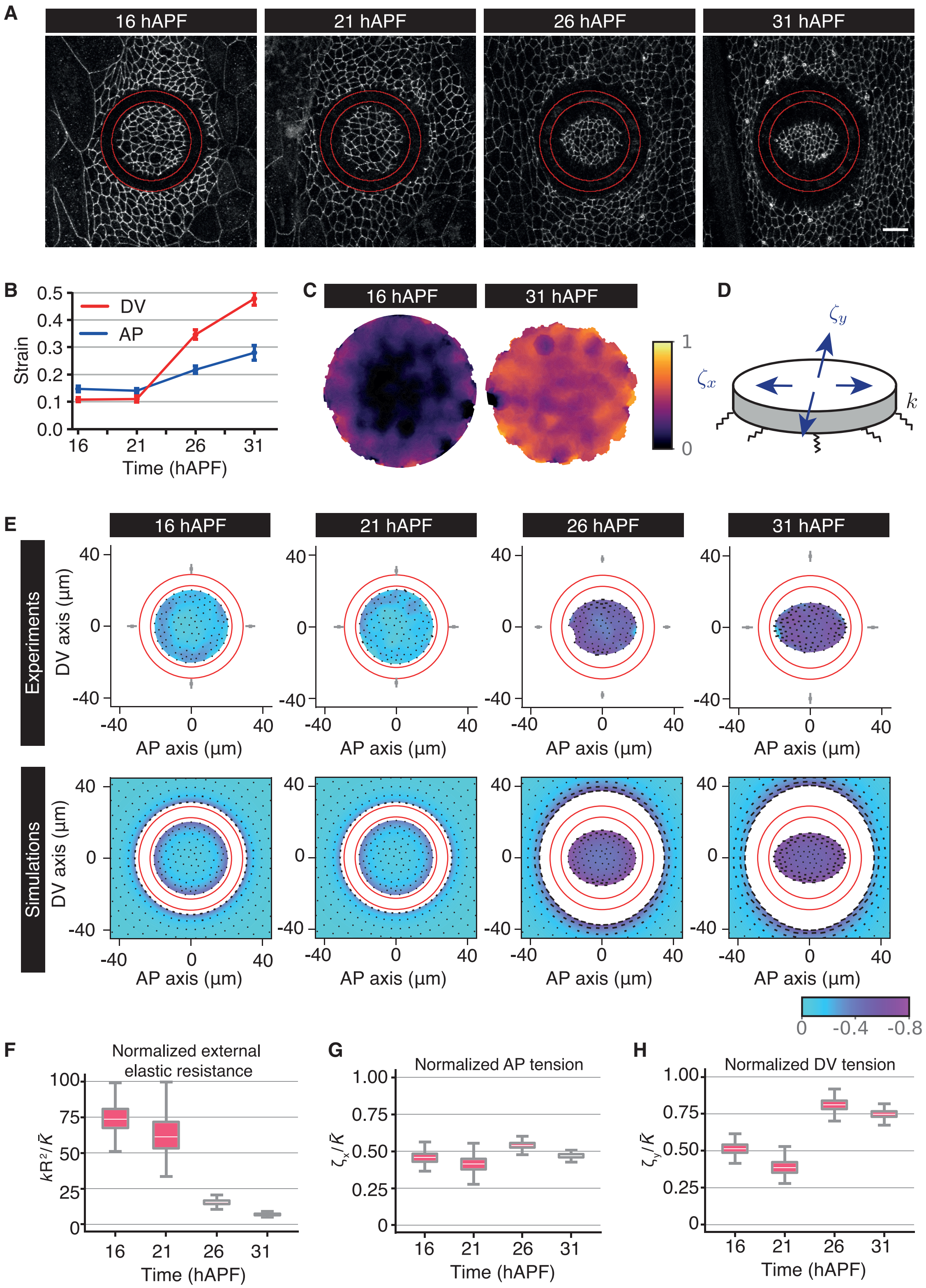

Figure 3. Deformation of excised histoblasts reveals a reduction in external resistance to histoblast movement

(A) Example time-lapse confocal images of histoblasts at the resting state following an annular ablation (ablated region outlined in red) in *E-cad*::*GFP*-expressing animals at different stages of abdominal development. Scale bars, 5 μm. See also Video S3.

(legend continued on next page)

over time. At 16 hAPF, cell area contraction was mostly confined to the disc edge, but at later developmental time points this edge bias had disappeared (Figure 3C). Since a free elastic disc under isotropic tension should constrict uniformly (Methods S1), we reasoned that this observation indicated that cell movement was limited by an external elastic resistance at early time points. We therefore compared experimental patterns of isotropic shear (relative cell area change) and anisotropic shear (change in cell elongation) with a continuum model where the tissue is described as an elastic material under active tension, adhering to an external substrate through elastic links (Figures 3D–3H and S3D–S3F). Fitting this model to spatial profiles of excised discs, and the deformation of the outer boundary of the ablated region (Figure 3E), we found that the parameter describing external resistance to disc deformation was strongly decreasing during development (Figure 3F). The model also indicated that the tissue internal AP tension, normalized to a cell elastic modulus, was roughly constant over time (Figure 3G), while the normalized DV tension increased after 26 hAPF (Figure 3H). We therefore conclude that the build-up of compressive stresses does not occur as the histoblasts cover the entire abdominal surface, and therefore is not responsible for growth arrest, as has been suggested for other tissues such as the wing imaginal disc.[3]

The basal extracellular matrix is degraded during histoblast expansion

Why does the external resistance to tissue deformation appear to decrease over time? The apical surface of the histoblasts and LECs is in contact with the pupal cuticle, while the basal surface is attached to a basement membrane containing collagen IV[31] (Viking [Vkg] in flies) (Figure S4A). We therefore quantified basal ECM dynamics during pupal abdominal development (Video S5). We investigated the dynamics of the three major *Drosophila* basal ECM components, perlecan (*Drosophila* Trol), collagen IV, and laminin B1 (LanB1) from 4 to 32 hAPF. During the pre-pupal stages (4–12 hAPF), all three components are present as a dense network across the entire abdomen (Figures 4A and S4B) but are slowly degraded (Figures 4A, 4B, and S4B; Video S5). At 12 hAPF, head eversion compresses the ECM network along the AP axis, leading to an increase in ECM component intensity (Video S5; Figures 4A, 4B, and S4B). This is followed by degradation of all three ECM components across the entire abdominal region from around 13 hAPF, consistent with previous work indicating that collagen IV under the histoblasts is degraded between 16 and 28 hAPF.[31] We found that perlecan is degraded at a faster rate than collagen IV and LanB1 (Figure 4B; Video S5). We examined matrix degradation at a higher spatial resolution after 16 hAPF. Both perlecan and collagen IV are degraded at a similar rate under both cell populations, and by 21 hAPF we only detect a residual punctate signal in hemocytes (Figures 4C, 4D, and S4C; Video S5). LanB1, on the other hand, is degraded under the LECs, but is only partially lost under the histoblasts, (Figures 4D and S4C; Video S5).

To test whether the reduction in basal ECM components caused the loss of external mechanical resistance inferred from annular ablations, we overexpressed matrix metalloprotease 1 (MMP1) or the tissue inhibitor of metalloproteases (TIMPs, an endogenous MMP inhibitor) in the LECs (*32B-GAL4* driver) and performed annular cuts on the histoblasts. MMP1 overexpression resulted in greater strain and recoil velocity than TIMP overexpression, although both showed fairly consistent values throughout development (Figures 4E, 4F, and S4D–S4I). As the wild-type strain values showed an increase after 21 hAPF, we wanted to compare the strain values in both MMP and TIMP overexpression with those of wild type. At early time points, MMP overexpression exhibit higher strain after ablation than wild type, but by 26 hAPF both conditions were similar (Figures 4G and 4H). Conversely, at 16 hAPF, TIMP overexpression showed slightly lower strain after ablation than wild type, and by 26 hAPF TIMP overexpression had failed to show any increase in strain (Figures 4G and 4H). It was not possible to perform and analyze ablations in TIMP overexpression pupae at 31 hAPF due to internalization of E-cadherin (see later time points in Video S6). We next examined area contraction across the excised discs in MMP1 and TIMP overexpressing pupae. At 21 hAPF, MMP1 overexpressing pupae exhibited less restriction to the edge of discs compared with wild type, and the overall deformation magnitude of the disc was more pronounced (Figure 4I). In contrast, in TIMP overexpression at 26 hAPF, the area deformation profile was concentrated near the boundary of the disc, and the overall deformation magnitude was reduced compared with wild type (Figure 4I). These results are intuitively consistent with the ECM providing external resistance to tissue deformation, as resistance decreases when the ECM is degraded early (MMP1 overexpression) and increases when the ECM persists for longer (TIMP overexpression). To obtain a quantitative readout, we fitted our continuum model to experimental deformation profiles in MMP1 and TIMP overexpression, in the same manner as for annular ablations in wild-type pupae (Figure S4J). This confirmed that external resistance to the disc deformation was decreasing early in MMP1 overexpression (Figure 4J) and was strongly

(B) Quantification of Hencky's true strain in the dorsal-ventral (DV) and anterior-posterior (AP) axes of excised histoblasts throughout development. n = 23, 24, 29, and 16 experiments at successive times. See also Figure S3.

(C) Averaged spatial map of absolute value of relative area change after excision at 16 and 31 hAPF, plotted on the undeformed discs (n = 20 [16 hAPF] and n = 15 [31 hAPF] experiments). As time progresses, the relative area change becomes more homogeneous in the disc.

(D) The excised disc is described as an elastic material, subjected to anisotropic active tension ζ_x, ζ_y, adhering to a substrate through elastic links, with effective elastic modulus per area k.

(E) Experiment (top) and simulation (bottom) deformation plots of excised histoblast discs. Color code: relative area change, black lines: anisotropic shear or change in cell elongation. Deformation fields are plotted on the deformed disc and are obtained from measurements before and 3 m after ablation for experiments. Red circles indicate ablated region. Gray squares and error bars in experimental plots: deformation of the outer circle of the ablated ring (mean ± 95% confidence interval), averaged between top/down and left/right deformations. Data are obtained from n = 20, 12, 13, and 15 experiments at successive times. See also Methods S1.

(F–H) Fitted model parameters to excised disc deformations, as a function of time. (F) Normalized ratio of external elastic modulus per area k to tissue elastic modulus $\overline{K}$. (G and H) Normalized AP and DV tensions, respectively. See also Figure S3.

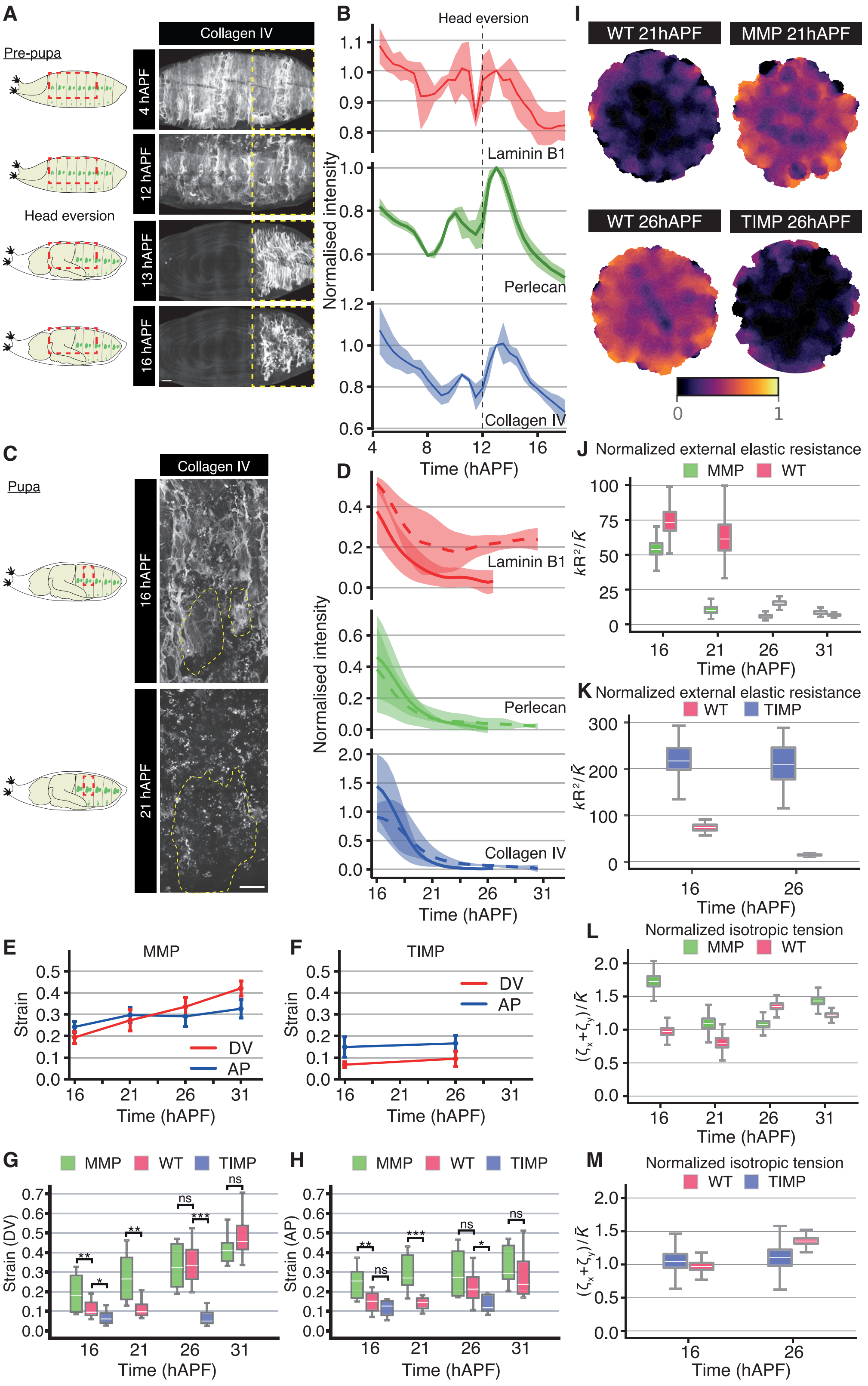

Figure 4. Basal extracellular matrix degradation induces a reduction in external resistance

(A) Left: similar schematic representation as in Figure 1B. Right: example time-lapse confocal images of collagen IV during the early pupal stages. Region of quantification is outlined in yellow. Scale bars, 50 μm. See also Video S5.

(legend continued on next page)

increased in TIMP overexpression (Figure 4K). Tissue tension was generally similar to wild type in these perturbations, except for an increase of isotropic tension at 16 hAPF in MMP1 overexpression (Figures 4L, 4M, S4K, and S4L). Overall, these results are consistent with the progressive disappearance of essential ECM components after 13 hAPF, resulting in reduced external resistance to deformation of the histoblast epithelium.

Basal ECM remodeling is necessary for histoblast nest expansion

The expansion phase of abdominal development is initiated at a similar time to ECM degradation, following the cleavage stage (0–10 hAPF) and pause in proliferation (10–14 hAPF) (Figure 1). We therefore investigated whether blocking ECM remodeling by overexpressing TIMP (Figures S5A and S5B) had any effect on histoblast proliferation. Strikingly, no proliferation was observed in the histoblasts during the time period observed, from 16 to 31 hAPF (Figure 5A; Video S6), compared with the extensive proliferation seen in the wild-type histoblasts over the same time period (Figure 1; Video S2). The lack of proliferation was not due to early pupal death, since our experimental samples developed to the pharate adult stage with a normal head, thorax, and wings (Figures 5B and S5C). Moreover, a pupal wing imaged simultaneously with the abdomen in a TIMP-expressing pupa proliferated normally at 16 hAPF (Figure S5D; Video S6). In contrast, the larval abdomen failed to be replaced by the histoblasts, as evidenced by the lack of pigmentation and sensory bristles (Figures 5B and S5C). We examined the effects of TIMP or MMP1 overexpression on histoblast cell numbers at 16 hAPF, when the cells have entered the expansion phase (Figure 5C). Premature ECM degradation by MMP1 overexpression does not induce a significant increase in cell numbers at 16 hAPF, suggesting that histoblasts in wild-type conditions are proliferating at their maximum rate (Figure 5C). In contrast, upon TIMP expression, histoblast numbers average 259 ± 46 at 16 hAPF, which is very similar to the number in wild type during the proliferation pause (266 ± 18.5 at 14 hAPF; Figure 1C). This suggests that ECM remodeling is required for histoblasts to transition from the cleavage to the expansion phase.

How is ECM degradation triggered? The two matrix metalloproteases, MMP1 and MMP2, are responsible for most basement membrane turnover in *Drosophila*.[32] To identify potential mechanisms for ECM degradation, we imaged a transcriptional reporter for the secreted *Drosophila* MMP1.[33] While *mmp1* is not expressed in histoblasts, it is expressed in the LECs (Figure 5A). To examine whether MMP expression in the LECs led to the degradation of the basal ECM, we depleted *mmp1* and *mmp2* using RNAi specifically in LECs (*32B-GAL4* driver). Similar to TIMP overexpression, this resulted in histoblasts failing to proliferate and a naked abdomen in pharate adults (Figures 5A and 5B). Therefore, MMP expression in the LECs promotes basal ECM degradation, allowing the growth and expansion of the histoblasts.

Histoblasts divide with a narrow distribution of cycle times, uncorrelated with cell geometry

To analyze in detail the growth kinetics of histoblasts during the expansion phase following ECM degradation, we examined the cell division rate (number of cell divisions per unit time, relative to total cell number) from 16 hAPF onward (Figure 6A). This revealed an oscillatory behavior with a period of ~4 h, with three oscillatory peaks observed before the cell division rate started to decrease (around 28 hAPF) (Figure 6A). All movies exhibited similar dynamics in the decay of the cell division rate, although the oscillation peaks were variable in time between the different animals (Figure 6A). We explored whether the average cell-cycle duration was oscillating through time and found that, in contrast to the cell division rate, the average cell-cycle time (time between two divisions ~4.5 ± 0.6 h, mean ± SD over four analyzed movies) varied only weakly in time (Figures 6B and S6A).

A large body of work suggests that geometric constraints influence cell division, for instance cells with a larger surface area are generally more likely to divide than cells with a constrained area.[3,6,34] We therefore wondered whether there was a relationship between cell-cycle time (time between two divisions) and cellular geometric features. Surprisingly, the cell-cycle time was neither correlated with histoblast initial area at birth or average lifetime area (Figures 6C and S6B), nor mean cell elongation (Figure 6D). This suggests that geometric constraints do not strongly influence histoblast cell-cycle time and therefore do not account for the observed oscillations in cell division rate.

To further explore the basis for the cell division rate temporal oscillations, we examined whether cycle times of mother and daughter cells were correlated but found only a weak correlation

(B) Quantification of ECM components intensity within region shown in (A). Intensity values are normalized to values at 13 hAPF once head eversion is complete (ribbons: SD, n = 3 for each genotype). See also Figure S4.
(C) Left: similar schematic representation as in Figure 1B. Right: example time-lapse confocal images of collagen IV during the pupal stages. Histoblasts are outlined in yellow. Scale bars, 50 μm. See also Video S5.
(D) Quantification of ECM components intensity underneath LECs (solid lines) and histoblasts (dotted lines), after subtraction and normalization to the lowest measured ECM component intensities under LECs (STAR Methods), throughout pupal development (ribbons: SD, n = 3 for each genotype). See also Figure S4.
(E and F) Quantification of Hencky's true strain of excised histoblasts after laser ablation, along the DV and AP axes throughout development, in pupae expressing MMP1 (E; n = 12, 7, 7, and 6) and TIMP (F; n = 7, 9) under the control of *32B-GAL4*. See also Video S3 and Figure S4.
(G and H) Quantification of Hencky's true strain along the AP (G) and DV (H) axes, calculated from annular ablations in pupae expressing MMP1 or TIMP under the control of *32B-GAL4*. Wild-type data are repeated from Figure 3. Mann-Whitney test was used to compare populations, with *p > 0.05; **p > 0.01; ***p > 0.001. From left to right for both plots n = 12, 23, 7, 7, 24, 7, 29, 9, 6, and 16. See also Figure S4.
(I) Averaged spatial map of absolute value of relative area change after excision at 21 hAPF for MMP and wild type, and at 26 hAPF for TIMP and wild type, plotted on the undeformed discs. The relative area change is more homogeneous in the MMP perturbation than in the wild type, indicating reduced external resistance to tissue deformation. From left to right, n = 12, 7, 13, and 5 experiments.
(J–M) Fitted model parameters for excised discs deformation, as a function of time, compared with parameters for wild type (red; data as in Figures 3F and S3D).
(J and K) Normalized ratio of external elastic modulus per area k to tissue bulk elastic modulus $\overline{K}$ in pupae expressing MMP1 (green, J) and TIMP (blue, K).
(L and M) Normalized isotropic (sum of AP and DV tensions, $\zeta_x+\zeta_y$) tensions in pupae expressing MMP1 (green, L) and TIMP (blue, M). See also Methods S1.

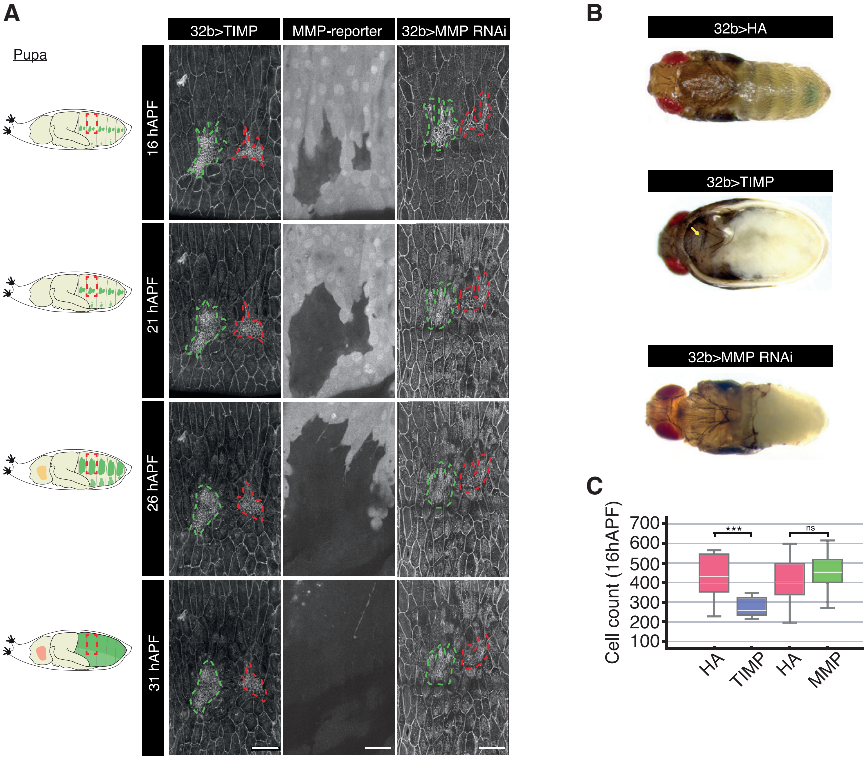

Figure 5. Basal ECM remodeling is necessary for histoblast nest expansion

(A) Left: similar schematic representation as in Figure 1B. Right: left column displays example time-lapse confocal images of a pupa expressing *E-cad::GFP* and overexpressing TIMP under the control of *32B-GAL4* at the time points indicated. Middle column displays example confocal images of the epidermis in a pupa expressing an MMP1-GFP reporter. Right column displays example time-lapse confocal images of a pupa expressing *E-cad::GFP* and overexpressing MMP RNAi under the control of *32B-GAL4* at the time points indicated. Anterior and posterior nests are outlined in green and red, respectively. Scale bars, 50 μm. See also Video S6.

(B) Images of pharate pupae expressing HA (control, top panel), TIMP (middle panel), and MMP RNAi (bottom panel) under the control of *32B-GAL4*. Yellow arrow indicates the normal development of the thorax. In contrast, the abdominal cuticle is unpigmented and devoid of sensory bristles.

(C) Quantification of total dorsal histoblast numbers from confocal images at 16 hAPF in pupae expressing HA (control, pink), TIMP (blue), and MMP RNAi (green) under the control of *32B-GAL4*. Mann-Whitney test was used to compare populations, with *p > 0.05; **p > 0.01; ***p > 0.001. From left to right for both plots, n = 22, 10, 26, and 13.

coefficient (Figures 6E and S6C, Pearson correlation coefficient $\rho = 0.04 \pm 0.04$). In contrast, we noticed that sister cell-cycle times were clearly correlated (Figures 6F and S6C, $\rho = 0.55 \pm 0.04$ for sisters). Given that cycle times are not correlated between generations, we wondered what other factors could explain the oscillations in proliferation rate. We noted that there is a relatively narrow cell-cycle time distribution (Figure S6A, coefficient of variation [CV] of cell-cycle times 0.22 ± 0.02, mean ± SD over four movies). Could the oscillations in cell division rate be attributed to this small variation in cell-cycle time? To test this, we simulated a growing population of cells with cell-cycle times chosen stochastically from a distribution comparable to the experimentally measured cell-cycle time distribution. We observed no obvious spatial patterns in cell-cycle time (Figure S6D); therefore, for simplicity, we did not take possible spatial relations in cell-cycle time into account. If cells initiate growth sufficiently synchronously, the cell division rate shows a damped oscillatory behavior, with the oscillation amplitude decaying as the population becomes progressively unsynchronized (see Figure S6E). This suggests that a narrow distribution of cell-cycle time together with synchronized initiation of growth (as the cells are released from the pause by ECM degradation) can contribute to the oscillatory behavior in cell division rates.

Histoblasts transition to growth arrest

We wished to determine why cell division rates decay strongly after ~28 hAPF (Figure 6A). In the *Drosophila* wing, growth termination is associated with a gradual increase in cell-cycle time from around 6 to 10 h increasing to over 20 to 30 h at the larval-pupal transition.[35–39] In contrast, we found that histoblast cell-cycle times were nearly constant throughout development, with a slight decrease around 22 hAPF, and do not increase at later stages to an extent that can account for growth arrest (Figure 6B). The discrepancy between average cycle times and average cell division rate implies that a fraction of histoblasts stop dividing. We therefore computationally labeled cells that did not divide until the end of our movies, which we denote as arrested histoblasts. Labeling these arrested cells revealed a spreading pattern of emerging arrested histoblasts, which progressively cover the entire histoblast nest (Figure 6G; Video S7). These arrested cells start to appear with a low probability around 24 hAPF, and after 28 hAPF nearly all newborn cells are arrested (Figure 6H). To determine at which phase of the cell cycle the histoblasts arrest, we quantified the intensity of FUCCI markers during histoblast expansion (Figure 6G). This revealed a gradual disappearance of the RFP-CycB marker, such that by 31 hAPF all cells are only expressing the G1 marker GFP-E2F1 (Figures 6G and 6I). Thus, following the expansion period, growth termination in the histoblasts occurs through G1/G0 arrest.

Do arrested histoblasts appear in a systematic spatial pattern within the nest? To test this, we labeled all tracked arrested cells in the final frame of our movies (which, by our definition, have all exited the cell cycle) according to their time of birth and found no consistent spatial trend across all wild-type movies (Figure 6J). We therefore treated the appearance of arrested cells as a stochastic process and quantified, for every cell division, the probability to give rise to one or two arrested cells (p) and, given that at least one of the daughter cells is arrested, the probability that both the daughter cells are arrested (α) (Figures 6K and 6L). The probability p and α increased sharply from about 0 before 24 hAPF to almost 1 after 28 hAPF, indicating that histoblasts

CellPress
OPEN ACCESS

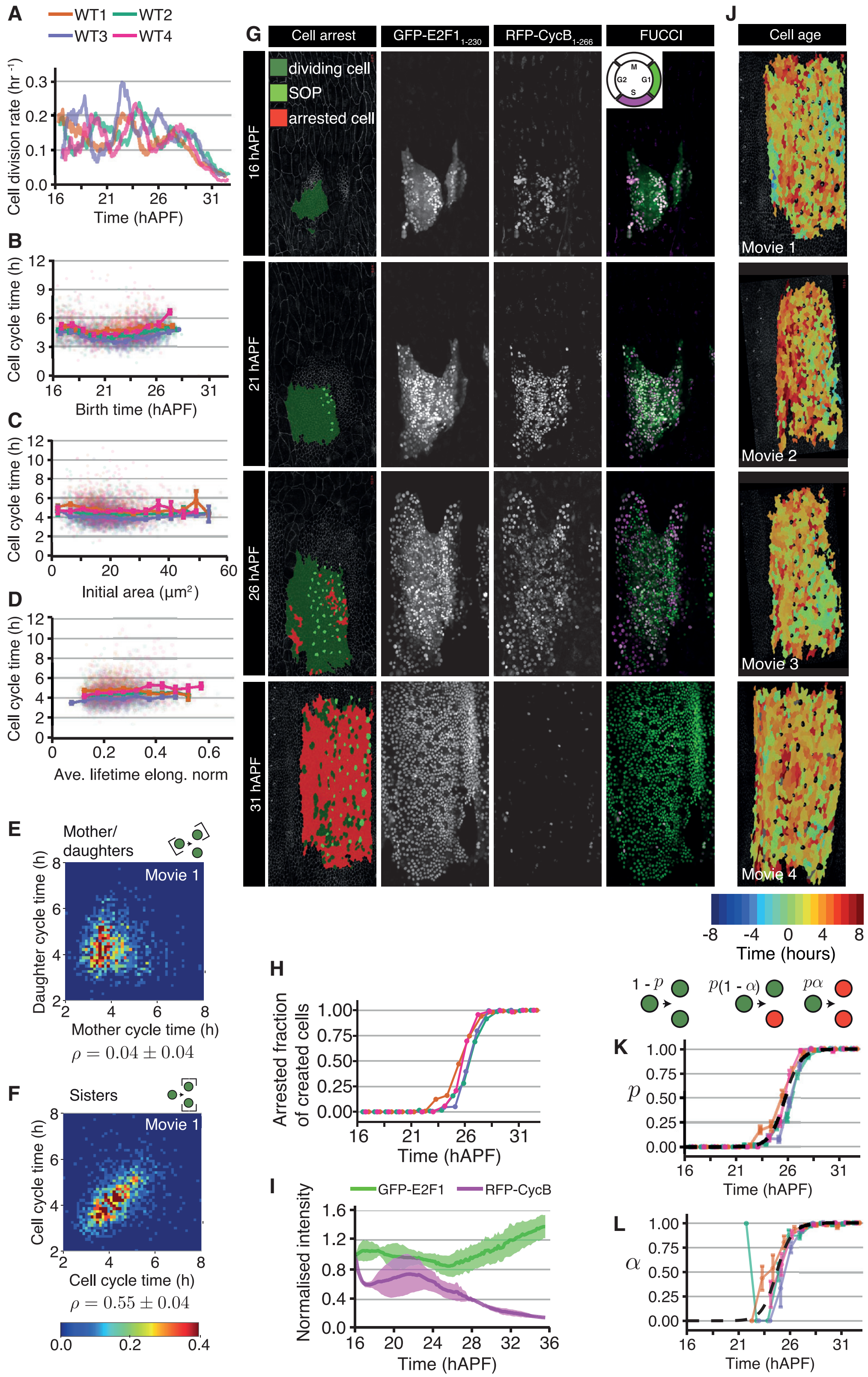

Figure 6. Histoblast cell-cycle times and transition to proliferative arrest

(A) Cell division rate in each wild-type movie as a function of time (after application of moving average, with a top-hat smoothing kernel with window size 0.875 h). The cell division rate oscillates up to ~28 hAPF before decaying. Data from noborder ROIs. See also Figures 1 and S6 and Methods S1.

(legend continued on next page)

transition abruptly to proliferative arrest, rather than gradually increasing cell-cycle times as reported in other tissues like the *Drosophila* wing disc.

Simulations recapitulate cell number increase from 0 to 36 hAPF

We wanted to know whether (1) a pause in proliferation between the cleavage and expansion phases, (2) cell-cycle times randomly chosen from a narrow distribution, and (3) a stochastic transition to cell-cycle arrest with a probability changing over time could quantitatively account for histoblast growth. We performed numerical simulations of tissue growth where (1) sister cells take a cycle time at their birth out of a bivariate normal probability distribution with a time-varying mean, CV, and fixed sister correlation coefficient; (2) cells pause their divisions between 12.5 and 14.7 hAPF but still age, giving rise to a burst of cell division around 15 hAPF; (3) cells stop proliferating according to the experimentally measured probability distribution; and (4) cells transition to SOPs with a fixed probability in a time window (Figure 7A; Methods S1). The mean and SD of the simulated cycle time probability distribution before 3.3 hAPF and after 14.7 hAPF were taken according to experimentally measured distributions (Figure 7B; Methods S1). Between these two phases, the mean cell-cycle time was taken as the starting value of the expansion phase at ~15 hAPF (Figure 7B). This model of tissue growth could closely match increases in the total number of cells, the total number of arrested cells, and the number of SOPs over time (Figures 7C–7E; see Figure S7 for effect of changing model parameters). The simulated cell division rate exhibited oscillations comparable in period and amplitude with experimental data (Figures 7F and 7G). As expected, oscillations in the simulated cell division rate were strongly dependent on the CV of cell-cycle time, becoming much flatter with less precise cycle times (Figures S7Q–S7S). We conclude that the essential features of histoblast expansion are accounted for by the simple rules used in our simulations. We do not exclude that additional synchronization mechanisms further participate in sustaining the oscillatory behavior of the cell division rate, as the magnitude of oscillatory peaks in cell division did not decrease over time as clearly in experiments as in simulations (Figure 7F).

DISCUSSION

Understanding how coordinated cellular behaviors dictate developmental tissue growth is key to elucidating the basis of tissue size and shape. Our quantitative analysis has allowed us to identify the major landmarks of abdominal growth. Following ~3 cleavage divisions after the onset of pupariation, a pause in cell division rate occurs until the degradation of the basal ECM by the LECs following head eversion (Figures 1, 4, and 5). This initiates an expansion phase during which cell division rates display an oscillatory profile (Figure 6). The expansion phase ends after ~4 cell divisions (Figure S7A), with cells stochastically transitioning to arrest (Figure 7). Beyond this global picture of abdominal growth, our dataset and simulations provide several important insights on the regulation of tissue growth and mechanics.

The switch between the prepupal cleavage divisions to the expansion phase represents a key step in abdominal development. Similar to the analogous switch taking place at the mid-blastula transition in many metazoan embryos,[7] this involves both a lengthening of the cell cycle and a resumption of tissue growth. The depletion of cyclin E stores accumulated during the larval growth period is thought to account for the increase in cell-cycle length.[22] Here, we show that the remodeling of the basal ECM is an essential step to allow the switch between cleavage divisions and expansion phase (Figure 5). Dynamic remodeling of the ECM is instrumental in orchestrating organ developmental processes such as cell migration and rearrangements.[32,40] We observe partial degradation of the basal ECM after head eversion is complete; with collagen IV and perlecan being completely degraded, while some laminin persists beneath the histoblasts. This degradation is required for the histoblasts to re-enter the cell cycle and for LECs to undergo extrusion from the epithelial layer (Figure 5; Video S6). The two *Drosophila* MMP family members, MMP1 and MMP2, promote remodeling of many tissues during metamorphosis.[32,41] In the abdominal epithelium, MMP expression is limited to LECs,

(B) Cell-cycle time as a function of birth time for each wild-type movie. Larger dots: binned data. Error bars: SEM for the bin. Faint smaller dots: individual data points. Data from noborder ROIs.

(C) Cell-cycle time as a function of initial cell apical area for each wild-type movie. Larger dots: binned data. Error bars: SEM for the bin. Faint smaller dots: individual data points. Data from noborder ROIs.

(D) Cell-cycle time as a function of the cell apical elongation, averaged over the lifetime of the cell. Larger dots: binned data. Error bars: SEM for the bin. Faint smaller dots: individual data points. Data from noborder ROIs.

(E and F) Probability density of pairs of cell cycle times, where pairs are taken from mother-daughters (E) and sisters (F). Cells are taken from the visible anterior nests. ρ, Pearson correlation coefficient (Methods S1). Mean ± SD are calculated over the values for the four wild types. Spearman correlation coefficients are ρ_S = 0.06 ± 0.04 (mother-daughters), ρ_S = 0.62 ± 0.03 (sisters) See also Figure S6.

(G) Left column: confocal images of histoblasts in live pupa expressing *E-cad::GFP* at the time points indicated overlaid with cell labels for SOPs and arrested cells. Middle-left to right columns: example confocal images of pupa expressing the FUCCI cell-cycle reporter markers GFP-E2F1 (middle-left) and RFP-CycB (middle-right) at the time points indicated. Schematic on far right shows expected combined color for cells at the different stages of the cell cycle. Scale bars, 50 μm. See also Video S7, Figure S1, and Methods S1.

(H) Fraction of created cells that is arrested as a function of time, for all wild-type movies (color code as in A–D).

(I) Quantification of the normalized total intensity for each FUCCI marker (ribbons: SD, n = 3).

(J) Snapshots of the final frame for each WT movie, with arrested cells colored by the time of their appearance, relative to each movie's "switch time" for the probability of arrested cell creation (see K).

(K and L) Schematic defining the parameters p and α characterizing arrested cell creation. Probability p that a division creates at least one arrested cell as a function of time (K). Each movie's p curve can be fitted with its own Hill function (not shown). Probability α that a cell division gives rise to two arrested cells, conditioned on the cell division giving rise to at least one arrested cell (L). Black dashed line, manual fit to the average movie behavior. See also Methods S1.

CellPress
OPEN ACCESS

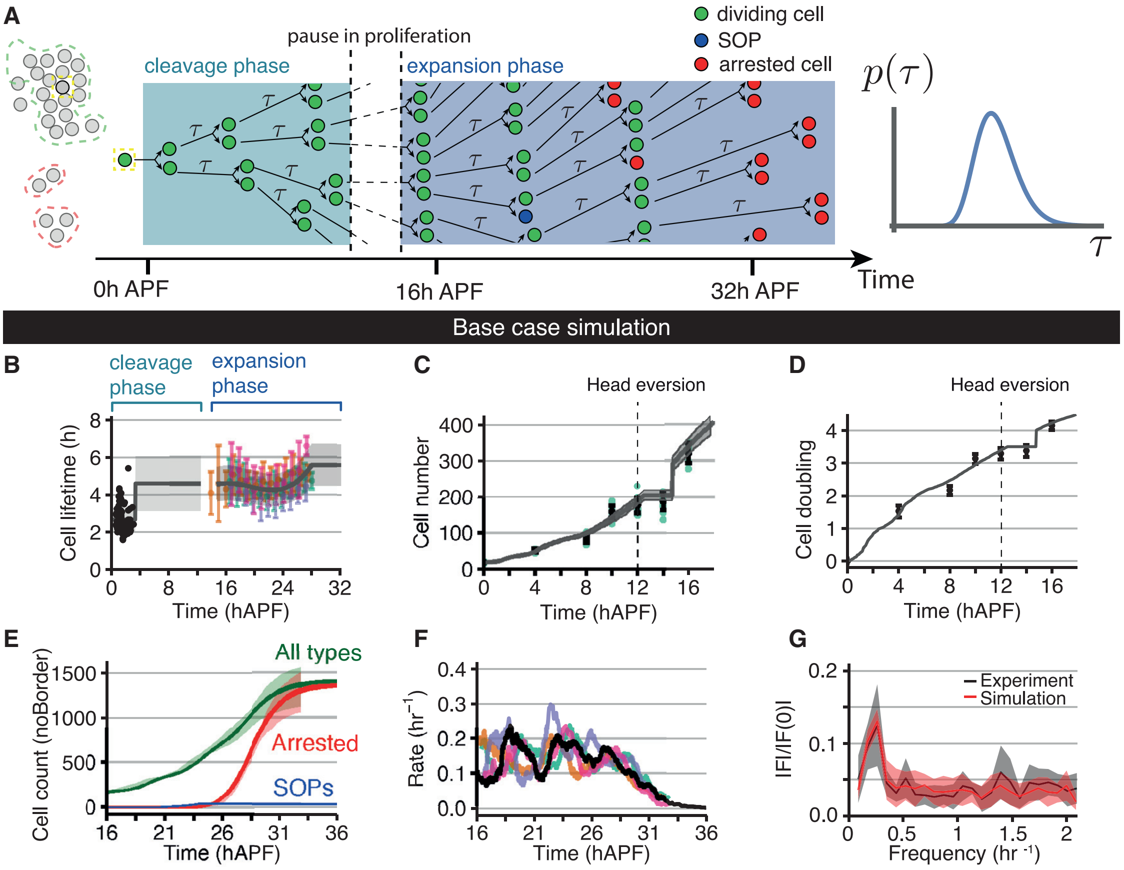

Figure 7. Kinetics of histoblast growth can be explained by changes in cell-cycle times and stochastic transition of individual cells to an arrested state

(A) Schematic of histoblast growth simulations. Cells take their cycle time from a probability distribution changing in time and become arrested or SOPs according to time-evolving probabilities. See also Figure S7 and Methods S1.

(B) Black points: experimental measurements of cycle times available in the first 3 hAPF. Colored points and error bars: binned mean and SD of cycle times from wild-type movie 1 to movie 4. Gray line and ribbon: mean and SD of cell-cycle time inputted to the simulation. In the time period covered by the colored points, the gray line is a 3rd-order polynomial fit to the data points.

(C) Quantification of anterior nest cell numbers from 0 hAPF until 16 hAPF (green dots, with average and SD indicated in black) with trajectories from representative simulations as described in Methods S1 (mean gray line, with SD indicated with ribbon). During the first 12 h of pupal development, histoblasts undergo three divisions followed by a transient pause in cell number increase until the expansion phase begins at around 15 hAPF.

(D) Quantification of anterior nest cell doublings from 0 to 16 hAPF with mean of representative simulations, as in (C).

(E) Comparison of normalized cell numbers for different cell classes in the noborder ROI between experimental measurements (lighter ribbons) and base case simulations (darker lines and ribbons). Cell numbers for different wild types are normalized, as described in Methods S1.

(F) Comparison of the experimental cell division rates within the noborder ROI from each wild-type movie (colored lines) with a representative simulation (black line). Moving average applied as in Figure 6A.

(G) Absolute value of the Fourier transforms of the division rate data prior to 28.5 hAPF, normalized to its value at 0 frequency. The height and sharpness of the main peak around 0.25 h^{-1} characterizes the oscillatory component of waves of division. Black: mean and SD from n = 4 experiments; red: mean and SD from n = 10 simulations.

and this expression is essential to trigger histoblast nest expansion (Figure 5).

How is the degradation of the basal ECM triggered at the correct time? One possibility is through a similar mechanism to fat body cells, which secrete MMP1 and MMP2 upon the pupation ecdysone pulse, causing the destruction of the cell-cell junctions and ECM that hold them together between 6 and 12 hAPF.[42,43] Alternatively, MMP expression and activity have been shown to be dependent on ECM/cellular mechanical properties and compaction.[44–48] As ECM degradation mainly occurs after head eversion, when the abdominal basal ECM and LECs undergo a massive compaction (Figure 5A; Video S5), it is possible that MMP expression in the LECs and subsequent ECM degradation are induced by compression of the LECs and basement membrane.

How does ECM degradation enable tissue growth? One possibility is that loss of signaling from the ECM, via integrins, signals the onset of the expansion divisions. However, this would be unexpected as in many systems integrin signaling promotes rather than inhibits growth and division.[49] Alternatively, basement membrane remodeling might free a source of trapped growth factor/morphogen, or allow a ligand present in the hemolymph

to access the histoblasts. Indeed, the ECM has been shown to act as a growth factor reservoir[50,51] or to restrict morphogen diffusion[52–54] in several tissues. It is interesting to consider this repressive effect of the ECM on developmental proliferation in the context of the proposed role of the normal cellular microenvironment in limiting cancer formation.[55] It is likely that oncogenic transformation involves the exploitation of developmental ECM remodeling mechanisms to convert the ECM from an anti-tumor to a pro-tumor environment that promotes proliferation and tissue invasion.[55,56]

Once growth has been initiated upon ECM degradation, we observed that the histoblast division rate exhibits temporal oscillations between ~16 and ~28 hAPF. Individual cell-cycle times are not correlated between mothers and daughters, nor are they dependent on cell geometry, but have a relatively tight statistical distribution (CV of 0.22). Simulations of a growing population of cells with such a narrow cell-cycle time distribution, initiating their cycle in a synchronized manner, likewise exhibit an oscillatory cell division rate (Figure S6E). However, our simulations show a dampening in amplitude of the proliferation rate due to cell divisions becoming less synchronized, which is not observed in the experimental data (Figures 7 and S7). It would be interesting to explore if further synchronization mechanisms contribute to sustain the oscillatory behavior observed experimentally. These oscillations cease once cells start exiting the cell cycle, and growth termination occurs (Figure 6).

The *Drosophila* wing imaginal disc is arguably the system in which growth and final size control have been studied most extensively.[11] A striking aspect of that system is that cell division decelerates progressively during roughly half of the larval growth period,[11] similarly to post-natal growth of the mouse skin, where the cell-cycle time of epidermal progenitors gradually increases over 60 days following birth.[57] In contrast, we find that termination of abdominal tissue growth is not mediated by a progressive increase in cell-cycle time, but by the sharp, stochastic transition of a growing population of cells to G1/G0 arrest, with no visible large-scale pattern (Figure 6). This is reminiscent of models proposed for growth and differentiation of neurons in the retina,[58] growth of mouse intestinal crypts,[59] or the expansion of the mouse embryonic skin.[60] In these tissues, a relatively abrupt transition in modes of progenitor divisions switch the tissue from expansion to maintenance and differentiation. This suggests that different developing tissues can achieve reproducible sizes using radically different growth termination strategies. It is possible that the sharp transition we observe here allows for fast expansion of the histoblast nest, at the cost of a less refined control over the final number of cells than would be allowed by a progressive slowdown in proliferation.[59]

Mechanical control of growth is an attractive model for tissue size determination and is supported by the study of the effects of cellular density on tissue culture cell proliferation[3,61] as well as in the wing disc.[29,62] In this scenario, crowding leads to cell-cycle arrest as cells reach confluence through compaction, driving a reduction in cell area. By analogy to cells in culture gradually filling empty space on the culture dish surface, it is tempting to speculate that histoblasts, which grow within a confined planar surface, are subjected to mechanical crowding leading to growth termination. However, we did not find a signature of cell area affecting the cell division rate (Figures 6 and S6). Furthermore, laser ablation experiments indicate that the tissue tension, normalized to cell elasticity, is either constant (in the AP direction) or slightly increasing (in the DV direction) (Figures 2, 3, and S3). Decreased Myo II accumulation has been proposed to mediate the effect of cell crowding on proliferation in the wing disc,[29,62] and we observe a similar reduction in junctional Myo II. Although an area of lower apical Myo II levels emerges in the medial part of the anterior dorsal nest from around 21 hAPF (Figures 2K and 2L), this does not lead to a similar pattern of increased cell-cycle time or early exit from the proliferation phase (Figure 6J). Finally, the transition from growth to arrest in the abdomen occurs between ~24 and ~28 hAPF, whereas the displacement of the last LECs and fusion of the dorsal nests at the midline takes place between 32 and 36 hAPF.[19,25] Thus, changes in cell geometry or mechanical tension do not appear to provide a universal growth termination cue. Instead, the histoblasts could sense the decline of a pro-growth signal secreted by the disappearing LECs, or the accumulation of a self-secreted inhibitor (chalone).[1] In addition, systemic signals such as the steroid hormone ecdysone or limiting nutrient availability could also play a role in growth termination in the abdomen.[2] Given the robustness of organ size across species, it is likely that several signals work in concert to ensure accurate size control.

STAR★METHODS

Detailed methods are provided in the online version of this paper and include the following:

- KEY RESOURCES TABLE
- RESOURCE AVAILABILITY
  - Lead contact
  - Materials availability
  - Data and Code Availability
- EXPERIMENTAL MODEL AND SUBJECT DETAILS
  - Fly stocks
- METHOD DETAILS
  - Fly rearing
  - Imaging
  - Laser ablations
  - Quantification of cell number
  - Fluorescence quantification
- QUANTIFICATION AND STATISTICAL ANALYSIS

SUPPLEMENTAL INFORMATION

Supplemental information can be found online at https://doi.org/10.1016/j.cub.2022.01.045.

ACKNOWLEDGMENTS

We thank Y. Bellaïche, D. Bohmann, B. Stramer, K. Irvine, M. Uhlirova, and the Bloomington *Drosophila* Stock Centre for fly stocks. We are grateful to M. Renshaw (Crick Advanced Light Microscopy Facility) and the Crick Fly Facility for support, and R. Etournay, M. Popovic, M. Merkel, and H. Brandl for help with Tissue Miner. We are grateful to B. Aerne for generating the UAS-HA fly stock and H. Gong, J. Frith, and B. Tapon for help with skeleton correction and image analysis. We thank J.P. Vincent and J. Briscoe for critical reading of the manuscript. J.R.D. is funded by a Sir Henry Wellcome Fellowship (201358/Z/16/Z). A.F. is funded by the European Union's Horizon 2020 research and innovation program under the Marie Skłodowska-Curie grant agreement MSCA-IF-EF-ST

no. 795060. This work was supported by a Wellcome Trust Investigator award (107885/Z/15/Z) to N.T. Work in the Salbreux and Tapon laboratories was supported by the Francis Crick Institute, which receives its core funding from Cancer Research UK (FC001317 and FC001175), the UK Medical Research Council (FC001317 and FC001175), and the Wellcome Trust (FC001317 and FC001175).

AUTHOR CONTRIBUTIONS

Conceptualization, all authors; acquisition, J.R.D., A.F., G.S., and N.T.; experiments, A.P.A., J.R.D., and A.F.; theory and simulations, J.J.W., A.T.-S., and G.S.; experimental methodology, F.M. and E.M.-B.; software, J.R.D., J.J.W., A.T.-S., A.H., and M.B.S; supervision, G.S. and N.T.; writing – original draft, A.P.A., J.R.D., J.J.W., A.F., A.H., M.B.S., G.S., and N.T.; writing – review & editing, all authors.

DECLARATION OF INTERESTS

The authors declare no competing interests.

Received: March 15, 2021
Revised: November 30, 2021
Accepted: January 18, 2022
Published: February 14, 2022

REFERENCES

1. Penzo-Méndez, A.I., and Stanger, B.Z. (2015). Organ-size regulation in mammals. Cold Spring Harb. Perspect. Biol. *7*, a019240.
2. Boulan, L., Milán, M., and Léopold, P. (2015). The systemic control of growth. Cold Spring Harb. Perspect. Biol. *7*, a019117.
3. Irvine, K.D., and Shraiman, B.I. (2017). Mechanical control of growth: ideas, facts and challenges. Development *144*, 4238–4248.
4. Sauls, J.T., Li, D., and Jun, S. (2016). Adder and a coarse-grained approach to cell size homeostasis in bacteria. Curr. Opin. Cell Biol. *38*, 38–44.
5. Loeffler, D., and Schroeder, T. (2019). Understanding cell fate control by continuous single-cell quantification. Blood *133*, 1406–1414.
6. LeGoff, L., and Lecuit, T. (2015). Mechanical forces and growth in animal tissues. Cold Spring Harb. Perspect. Biol. *8*, a019232.
7. Farrell, J.A., and O'Farrell, P.H. (2014). From egg to gastrula: how the cell cycle is remodeled during the *Drosophila* mid-blastula transition. Annu. Rev. Genet. *48*, 269–294.
8. Guirao, B., Rigaud, S.U., Bosveld, F., Bailles, A., López-Gay, J., Ishihara, S., Sugimura, K., Graner, F., and Bellaïche, Y. (2015). Unified quantitative characterization of epithelial tissue development. eLife *4*, e08519.
9. Etournay, R., Merkel, M., Popović, M., Brandl, H., Dye, N.A., Aigouy, B., Salbreux, G., Eaton, S., and Jülicher, F. (2016). TissueMiner: a multiscale analysis toolkit to quantify how cellular processes create tissue dynamics. eLife *5*, e14334.
10. Mavrakis, M., Pourquié, O., and Lecuit, T. (2010). Lighting up developmental mechanisms: how fluorescence imaging heralded a new era. Development *137*, 373–387.
11. Gou, J., Stotsky, J.A., and Othmer, H.G. (2020). Growth control in the *Drosophila* wing disk. Wiley Interdiscip. Rev. Syst. Biol. Med. *12*, e1478.
12. Mangione, F., and Martín-Blanco, E. (2018). The Dachsous/Fat/Four-jointed pathway directs the uniform axial orientation of epithelial cells in the *Drosophila* abdomen. Cell Rep *25*, 2836–2850.e4.
13. Prat-Rojo, C., Pouille, P.A., Buceta, J., and Martin-Blanco, E. (2020). Mechanical coordination is sufficient to promote tissue replacement during metamorphosis in *Drosophila*. EMBO J *39*, e103594.
14. Bischoff, M., and Cseresnyés, Z. (2009). Cell rearrangements, cell divisions and cell death in a migrating epithelial sheet in the abdomen of *Drosophila*. Development *136*, 2403–2411.
15. Hartenstein, V. (1993). Atlas of *Drosophila* Development (Cold Spring Harbor Laboratory Press).
16. Guerra, M., Postlethwait, J.H., and Schneiderman, H.A. (1973). The development of the imaginal abdomen of *Drosophila melanogaster*. Dev. Biol. *32*, 361–372.
17. Athilingam, T., Tiwari, P., Toyama, Y., and Saunders, T.E. (2021). Mechanics of epidermal morphogenesis in the *Drosophila* pupa. Semin. Cell Dev. Biol. *120*, 171–180.
18. Roseland, C.R., and Schneiderman, H.A. (1979). Regulation and metamorphosis of the abdominal histoblasts of *Drosophila melanogaster*. Wilehm Roux Arch. Dev. Biol. *186*, 235–265.
19. Madhavan, M.M., and Madhavan, K. (1980). Morphogenesis of the epidermis of adult abdomen of *Drosophila*. J. Embryol. Exp. Morphol. *60*, 1–31.
20. Garcia-Bellido, A., and Merriam, J.R. (1971). Clonal parameters of tergite development in *Drosophila*. Dev. Biol. *26*, 264–276.
21. Mandaravally Madhavan, M., and Schneiderman, H.A. (1977). Histological analysis of the dynamics of growth of imaginal discs and histoblast nests during the larval development of *Drosophila melanogaster*. Wilehm Roux Arch. Dev. Biol. *183*, 269–305.
22. Ninov, N., Chiarelli, D.A., and Martín-Blanco, E. (2007). Extrinsic and intrinsic mechanisms directing epithelial cell sheet replacement during *Drosophila* metamorphosis. Development *134*, 367–379.
23. Nakajima, Y., Kuranaga, E., Sugimura, K., Miyawaki, A., and Miura, M. (2011). Nonautonomous apoptosis is triggered by local cell cycle progression during epithelial replacement in *Drosophila*. Mol. Cell. Biol. *31*, 2499–2512.
24. Teng, X., Qin, L., Le Borgne, R., and Toyama, Y. (2017). Remodeling of adhesion and modulation of mechanical tensile forces during apoptosis in *Drosophila* epithelium. Development *144*, 95–105.
25. Michel, M., and Dahmann, C. (2020). Tissue mechanical properties modulate cell extrusion in the *Drosophila* abdominal epidermis. Development *147*, dev179606.
26. Zielke, N., Korzelius, J., van Straaten, M., Bender, K., Schuhknecht, G.F.P., Dutta, D., Xiang, J., and Edgar, B.A. (2014). Fly-FUCCI: a versatile tool for studying cell proliferation in complex tissues. Cell Rep *7*, 588–598.
27. Mangione, F., and Martin-Blanco, E. (2020). Imaging and analysis of tissue orientation and growth dynamics in the developing *Drosophila* epithelia during pupal stages. J. Vis. Exp. Published online June 2, 2020. https://doi.org/10.3791/60282.
28. Merkel, M., Etournay, R., Popović, M., Salbreux, G., Eaton, S., and Jülicher, F. (2017). Triangles bridge the scales: quantifying cellular contributions to tissue deformation. Phys. Rev. E *95*, 032401.
29. Rauskolb, C., Sun, S., Sun, G., Pan, Y., and Irvine, K.D. (2014). Cytoskeletal tension inhibits Hippo signaling through an Ajuba-Warts complex. Cell *158*, 143–156.
30. Umetsu, D., Aigouy, B., Aliee, M., Sui, L., Eaton, S., Jülicher, F., and Dahmann, C. (2014). Local increases in mechanical tension shape compartment boundaries by biasing cell intercalations. Curr. Biol. *24*, 1798–1805.
31. Ninov, N., Menezes-Cabral, S., Prat-Rojo, C., Manjón, C., Weiss, A., Pyrowolakis, G., Affolter, M., and Martín-Blanco, E. (2010). Dpp signaling directs cell motility and invasiveness during epithelial morphogenesis. Curr. Biol. *20*, 513–520.
32. Ramos-Lewis, W., and Page-McCaw, A. (2019). Basement membrane mechanics shape development: lessons from the fly. Matrix Biol *75–76*, 72–81.
33. Wang, Q., Uhlirova, M., and Bohmann, D. (2010). Spatial restriction of FGF signaling by a matrix metalloprotease controls branching morphogenesis. Dev. Cell *18*, 157–164.
34. López-Gay, J.M., Nunley, H., Spencer, M., di Pietro, F., Guirao, B., Bosveld, F., Markova, O., Gaugue, I., Pelletier, S., Lubensky, D.K., et al. (2020). Apical stress fibers enable a scaling between cell mechanical response and area in epithelial tissue. Science *370*, eabb2169.

35. Milán, M., Campuzano, S., and García-Bellido, A. (1996). Cell cycling and patterned cell proliferation in the wing primordium of *Drosophila*. Proc. Natl. Acad. Sci. USA *93*, 640–645.
36. Wartlick, O., Mumcu, P., Kicheva, A., Bittig, T., Seum, C., Jülicher, F., and González-Gaitán, M. (2011). Dynamics of Dpp signaling and proliferation control. Science *331*, 1154–1159.
37. Martín, F.A., Herrera, S.C., and Morata, G. (2009). Cell competition, growth and size control in the *Drosophila* wing imaginal disc. Development *136*, 3747–3756.
38. Mao, Y., Tournier, A.L., Hoppe, A., Kester, L., Thompson, B.J., and Tapon, N. (2013). Differential proliferation rates generate patterns of mechanical tension that orient tissue growth. EMBO J *32*, 2790–2803.
39. Johnston, L.A., and Sanders, A.L. (2003). Wingless promotes cell survival but constrains growth during *Drosophila* wing development. Nat. Cell Biol. *5*, 827–833.
40. Walma, D.A.C., and Yamada, K.M. (2020). The extracellular matrix in development. Development *147*, dev175596.
41. Diaz-de-la-Loza, M.D., Ray, R.P., Ganguly, P.S., Alt, S., Davis, J.R., Hoppe, A., Tapon, N., Salbreux, G., and Thompson, B.J. (2018). Apical and basal matrix remodeling control epithelial morphogenesis. Dev. Cell *46*, 23–39.e5.
42. Bond, N.D., Nelliot, A., Bernardo, M.K., Ayerh, M.A., Gorski, K.A., Hoshizaki, D.K., and Woodard, C.T. (2011). ssFTZ-F1 and matrix metalloproteinase 2 are required for fat-body remodeling in *Drosophila*. Dev. Biol. *360*, 286–296.
43. Jia, Q., Liu, Y., Liu, H., and Li, S. (2014). Mmp1 and Mmp2 cooperatively induce *Drosophila* fat body cell dissociation with distinct roles. Sci. Rep. *4*, 7535.
44. Petersen, A., Joly, P., Bergmann, C., Korus, G., and Duda, G.N. (2012). The impact of substrate stiffness and mechanical loading on fibroblast-induced scaffold remodeling. Tissue Eng. Part A *18*, 1804–1817.
45. Fleischhacker, V., Klatte-Schulz, F., Minkwitz, S., Schmock, A., Rummler, M., Seliger, A., Willie, B.M., and Wildemann, B. (2020). *In vivo* and *in vitro* mechanical loading of mouse Achilles tendons and tenocytes-a pilot study. Int. J. Mol. Sci. *21*, 1313.
46. Wang, Y., Yang, L., Zhang, J., Xue, R., Tang, Z., Huang, W., Jiang, D., Tang, X., Chen, P., and Sung, K.L. (2010). Differential MMP-2 activity induced by mechanical compression and inflammatory factors in human synoviocytes. Mol. Cell. Biomech. *7*, 105–114.
47. Yi, E., Sato, S., Takahashi, A., Parameswaran, H., Blute, T.A., Bartolák-Suki, E., and Suki, B. (2016). Mechanical forces accelerate collagen digestion by bacterial collagenase in lung tissue strips. Front. Physiol. *7*, 287.
48. Fitzgerald, J.B., Jin, M., Dean, D., Wood, D.J., Zheng, M.H., and Grodzinsky, A.J. (2004). Mechanical compression of cartilage explants induces multiple time-dependent gene expression patterns and involves intracellular calcium and cyclic AMP. J. Biol. Chem. *279*, 19502–19511.
49. Hamidi, H., and Ivaska, J. (2018). Every step of the way: integrins in cancer progression and metastasis. Nat. Rev. Cancer *18*, 533–548.
50. Bonnans, C., Chou, J., and Werb, Z. (2014). Remodelling the extracellular matrix in development and disease. Nat. Rev. Mol. Cell Biol. *15*, 786–801.
51. Hynes, R.O. (2009). The extracellular matrix: not just pretty fibrils. Science *326*, 1216–1219.
52. Wang, X., Harris, R.E., Bayston, L.J., and Ashe, H.L. (2008). Type IV collagens regulate BMP signalling in *Drosophila*. Nature *455*, 72–77.
53. Tian, A., and Jiang, J. (2014). Intestinal epithelium-derived BMP controls stem cell self-renewal in *Drosophila* adult midgut. eLife *3*, e01857.
54. Ma, M., Cao, X., Dai, J., and Pastor-Pareja, J.C. (2017). Basement membrane manipulation in *Drosophila* wing discs affects Dpp retention but not growth mechanoregulation. Dev. Cell *42*, 97–106.e4.
55. Bissell, M.J., and Hines, W.C. (2011). Why don't we get more cancer? A proposed role of the microenvironment in restraining cancer progression. Nat. Med. *17*, 320–329.
56. Chang, J., and Chaudhuri, O. (2019). Beyond proteases: basement membrane mechanics and cancer invasion. J. Cell Biol. *218*, 2456–2469.
57. Dekoninck, S., Hannezo, E., Sifrim, A., Miroshnikova, Y.A., Aragona, M., Malfait, M., Gargouri, S., de Neunheuser, C., Dubois, C., Voet, T., et al. (2020). Defining the design principles of skin epidermis postnatal growth. Cell *181*, 604–620.e22.
58. He, J., Zhang, G., Almeida, A.D., Cayouette, M., Simons, B.D., and Harris, W.A. (2012). How variable clones build an invariant retina. Neuron *75*, 786–798.
59. Itzkovitz, S., Blat, I.C., Jacks, T., Clevers, H., and van Oudenaarden, A. (2012). Optimality in the development of intestinal crypts. Cell *148*, 608–619.
60. Lechler, T., and Fuchs, E. (2005). Asymmetric cell divisions promote stratification and differentiation of mammalian skin. Nature *437*, 275–280.
61. Panciera, T., Azzolin, L., Cordenonsi, M., and Piccolo, S. (2017). Mechanobiology of YAP and TAZ in physiology and disease. Nat. Rev. Mol. Cell Biol. *18*, 758–770.
62. Pan, Y., Alégot, H., Rauskolb, C., and Irvine, K.D. (2018). The dynamics of Hippo signaling during *Drosophila* wing development. Development *145*, dev165712.
63. Pinheiro, D., Hannezo, E., Herszterg, S., Bosveld, F., Gaugue, I., Balakireva, M., Wang, Z., Cristo, I., Rigaud, S.U., Markova, O., and Bellaiche, Y. (2017). Transmission of cytokinesis forces via E-cadherin dilution and actomyosin flows. Nature *545*, 103–107.
64. Uhlirova, M., and Bohmann, D. (2006). JNK- and Fos-regulated Mmp1 expression cooperates with Ras to induce invasive tumors in *Drosophila*. EMBO J *25*, 5294–5304.
65. Rogulja, D., and Irvine, K.D. (2005). Regulation of cell proliferation by a morphogen gradient. Cell *123*, 449–461.
66. Buszczak, M., Paterno, S., Lighthouse, D., Bachman, J., Planck, J., Owen, S., Skora, A.D., Nystul, T.G., Ohlstein, B., Allen, A., et al. (2007). The carnegie protein trap library: a versatile tool for *Drosophila* developmental studies. Genetics *175*, 1505–1531.
67. Sarov, M., Barz, C., Jambor, H., Hein, M.Y., Schmied, C., Suchold, D., Stender, B., Janosch, S., Vinay Vikas, K.J., Krishnan, R.T., et al. (2016). A genome-wide resource for the analysis of protein localisation in *Drosophila*. eLife *5*, e12068.
68. Schindelin, J., Arganda-Carreras, I., Frise, E., Kaynig, V., Longair, M., Pietzsch, T., Preibisch, S., Rueden, C., Saalfeld, S., Schmid, B., et al. (2012). Fiji: an open-source platform for biological-image analysis. Nat. Methods *9*, 676–682.
69. Aigouy, B., Umetsu, D., and Eaton, S. (2016). Segmentation and quantitative analysis of epithelial tissues. Methods Mol. Biol. *1478*, 227–239.
70. Brand, A.H., and Perrimon, N. (1993). Targeted gene expression as a means of altering cell fates and generating dominant phenotypes. Development *118*, 401–415.
71. McGuire, S.E., Mao, Z., and Davis, R.L. (2004). Spatiotemporal gene expression targeting with the TARGET and gene-switch systems in *Drosophila*. Sci. STKE, 2004, pl6.
72. Liang, X., Michael, M., and Gomez, G.A. (2016). Measurement of mechanical tension at cell-cell junctions using two-photon laser ablation. Bio Protoc *6*, e2068.
73. Bonnet, I., Marcq, P., Bosveld, F., Fetler, L., Bellaïche, Y., and Graner, F. (2012). Mechanical state, material properties and continuous description of an epithelial tissue. J. R. Soc. Interface *9*, 2614–2623.

STAR★METHODS

KEY RESOURCES TABLE

REAGENT or RESOURCE	SOURCE	IDENTIFIER
**Deposited data**		
Corrected skeletons and Tissue Miner outputs of wild type Movies 1–4	This work	https://doi.org/10.25418/crick.c.5787494.v1
Myo II quantifications	This work	https://doi.org/10.25418/crick.c.5787494.v1
Cell cycle correlations	This work	https://doi.org/10.25418/crick.c.5787494.v1
**Experimental models: Organisms/strains**		
D. melanogaster: *y[1] w[*]; TI[TI]shg[GFP] (E-cad::GFP)*	Bloomington Drosophila stock center	BDSC Cat #60584; RRID: BDSC_60584
D. melanogaster: *y[1] w[*]; TI[TI]shg [3x mKate2] (E-cad::mKate2)*	Gift from Yohanns Bellaïche	[63]
D. melanogaster: *w[*]; P[w[+mW.hs]=GawB] 32B (32B-GAL4)*	Bloomington Drosophila stock center	BDSC Cat #1782; RRID: BDSC_1782
D. melanogaster: *w[*]; P*[w[+mC]= *UAS-Timp.P]3*	Bloomington Drosophila stock center	BDSC Cat #58708; RRID: BDSC_58708
D. melanogaster: w;;*UAS-MMP1*^{RNAi}, *UAS-MMP2*^{RNAi}	Gift from Mirka Uhlirova	[64]
D. melanogaster: *PBac[fTRG10075. sfGFP-FT]VK00033 (Sqh::GFP)*	Vienna *Drosophila* Resource Center	VDRC Cat #318484
D. melanogaster: *w[*]; P[w[+mC]=tubP-GAL80[ts]]2/TM2*	Bloomington Drosophila stock center	BDSC Cat #7017; RRID: BDSC_7017
D. melanogaster: *P[Mmp1.GFP]*	Gift from Dirk Bohmann	[33]
D. melanogaster: *y[1] w[1118]; UAS-HA/TM6b*	This work	N/A
D. melanogaster: *w[*]; vkg::GFP, E-cad:: mTomato*	Gift from Brian Stramer	N/A
D. melanogaster: *w[*]; P[w[+mC]= UAS-mCherry.NLS]3*	Bloomington Drosophila Stock Center	BDSC Cat #38424; RRID: BDSC 38424
D. melanogaster: *w[*]; act>y+>Gal4 UAS-GFP*	Gift from Kenneth Irvine	[65]
D. melanogaster: *y[1] w[*]; P[w[+mC]= UAS-FLP.D]JD1*	Bloomington Drosophila Stock Center	BDSC Cat #4539; RRID: BDSC 4539
D. melanogaster: *y[*] w[*]; [w[+mW.hs]= GawB]NP1248 / CyO,* P[w[-]=*UAS-lacZ. UW14]UW14 (esg*^{NP1248}*-GAL4)*	Kyoto Stock Center (DGRC)	DGRC 112589
D. melanogaster: *w[*] P[w[+mC]=PTT-un1] ZCL1700 (Perlecan-GFP)*	Kyoto Stock Center (DGRC)	DGRC 110807
D. melanogaster: *w[*], vkg (Col IV)::GFP*	Gift from Brian Stramer	[66]
D. melanogaster: *w[*]; PBac[fTRG00681. sfGFP-TVPTBF]VK00033 (lanB1::GFP)*	Brian Stramer	[67]
D. melanogaster: w[1118]; Kr[If-1]/CyO, P{ry[+t7.2]=en1}wg[en11]; P{w[+mC]= UAS-GFP-E2f1.1-230}26 P{w[+mC]= UAS-mRFP1.NLS-CycB.1-266} 17/TM6B, Tb[1]	Bloomington Drosophila Stock Center	BDSC Cat #55122; RRID: BDSC 55122
D. melanogaster: w[1118]; P{w[+mC]=Ubi-GFP-E2f1.1-230}19 P{w[+mC]=Ubi-mRFP1.NLS-CycB.1-266}15/CyO, P{ry[+t7.2] =en1}wg[en11]; MKRS/TM6B, Tb[+]	Bloomington Drosophila Stock Center	BDSC Cat #55123; RRID: BDSC 55123

(*Continued on next page*)

Continued		
REAGENT or RESOURCE	SOURCE	IDENTIFIER
Software and algorithms		
Fiji	[68]	http://fiji.sc
Matlab2014a	Mathworks	https://uk.mathworks.com/products/matlab
Mathematica 11.0.1.0	Wolfram	https://www.wolfram.com/mathematica/
Python, Numpy, Scipy	Python Software Foundation	https://www.python.org<br>https://numpy.org<br>https://www.scipy.org
RStudio	GNU	https://www.r-project.org/
Tissue Analyzer	[69]	https://grr.gred-clermont.fr/labmirouse/software/WebPA/
Tissue Miner	[9]	https://github.com/mpicbg-scicomp/tissue_miner
Skeletor	This work, Mathematica package	https://github.com/drjrdavis/Skeletor
Projection/Skeleton Correction tool/Tracker	This work, Matlab packages	https://doi.org/10.25418/crick.c.5787494.v1
Tissue Miner analysis scripts for wild type Videos S1, S2, S3, and S4	This work, R scripts	https://doi.org/10.25418/crick.c.5787494.v1
Growth_simulations	This work, Python scripts	https://doi.org/10.25418/crick.c.5787494.v1
Anabfem	This work, Python package	https://github.com/FrancisCrickInstitute/anabfem
GraphPad Prism 7	GraphPad Software	https://www.graphpad.com/scientific-software/prism/

RESOURCE AVAILABILITY

Lead contact

Further information and requests for resources and reagents should be directed to and will be fulfilled by the lead contact, Nicolas Tapon (nic.tapon@crick.ac.uk).

Materials availability

All reagents generated in this study are available from the lead contact without restriction.

Data and Code Availability

- All imaging data and quantifications have been deposited on FigShare are publicly available as of the date of publication. The DOI is listed in the key resources table.
- All original code has been deposited on GitHub or FigShare and is publicly available as of the date of publication. DOIs or GitHub links are listed in the key resources table.
- Any additional information required to reanalyze the data reported in this paper is available from the lead contact upon request.

EXPERIMENTAL MODEL AND SUBJECT DETAILS

Fly stocks

All experiments were performed in *Drosophila melanogaster* (see key resources table and figure genotypes table for details of strains used). Flies were maintained at 25°C (unless otherwise indicated) on food generated by the Francis Crick Institute Media Facility (360 g agar, 3600 g maize, 3600 g malt, 1200 mL molasses, 440 g soya, 732 g yeast extract, 280 mL of acid mix (500 mL propionic and 32 mL orthophosphoric acid) and 50 L water).

METHOD DETAILS

Fly rearing

Unless stated all flies were raised at 25°C. Pupae were collected at a pupal stage before head eversion, kept at 25°C and then monitored hourly for head eversion to calculate pupal age as 12 hAPF, four hours prior to imaging at 16 hAPF. For experiments blocking ECM degradation and time-matched controls, we modulated the expression of TIMP, under control of the larval epithelial cell specific

GAL4 promoter *32B-GAL4*, in a temperature-controlled manner. We crossed virgins of *E-cad::GFP*; *32BGAL4* to either males of *UAS-TIMP* or *UAS-HA* (III). For comparing collagen IV degradation we crossed virgins of *E-cad::Tom*, *vkg-GFP*; *32BGAL4* to either males of *UAS-TIMP* or *UAS-HA* (III). Crosses were kept at 25°C. Larvae were raised at 18°C and pupae placed at 25°C, 7 hours prior to imaging. Pupae were monitored hourly for head eversion to calculate pupal age as 12 hAPF, four hours prior to imaging at 16 hAPF. Pupae were kept at 25°C during imaging or rearing for specific developmental times.

For experiments promoting ECM degradation and time-matched controls, we modulated the expression of MMP with the *UAS-GAL4-GAL80^{ts}* system,[70,71] under control of the larval epithelial cell specific GAL4 promoter *32B-GAL4*. We crossed virgins of *E-cad::GFP, tubGAL80^{ts}*; *32BGAL4* to either males of *UAS-MMP1* or *UAS-HA* (III). Crosses were kept at 25°C. Larvae were raised at 18°C and pupae placed at 29°C, 7 hours prior to imaging. Pupae were monitored hourly for head eversion to calculate pupal age as 12 hAPF, just under three hours prior to imaging at 16 hAPF. At 16 hAPF pupae were switched and kept at 25°C during imaging or rearing for specific developmental times.

Imaging

Pupae were dissected and mounted as described.[27] All movies were acquired on a Zeiss LSM 880 confocal microscope at 25°C, with a Plan-Apochromat 40x/1.3 oil DIC M27 objective, unless stated. For the dorsal-lateral field of view, images were acquired as two-tiles of 1024x1024 pixels with a 10% overlap, with 20-30 z-slices 1μm apart, and the tiled stacks were fused in Zen Blue. wild-type *E-cad::GFP* movies were acquired with a frame every 2.5 minutes, TIMP overexpression movies with *E-cad::GFP* every 5 minutes, *E-cad::mCherry* and ECM components and FUCCI markers during the pupal stages every 10 minutes, *mmp1-GFP* every 20 minutes, and the *E-cad::mKate2; Sqh::GFP* movies every 5 hours. The TIMP overexpression movies with *vkg::GFP* were filmed at 29°C, every 20 minutes.

For imaging of the ECM, histoblast proliferation and FUCCI markers during pre-pupal stages, pre-pupae were placed dorsal-laterally with their posterior in Voltalef 10s to image the abdominal segments through the cuticle. Movies were filmed on a Zeiss LSM 880 confocal microscope at 25°C, with a Plan-Apochromat 40x/1.3 oil DIC M27 objective. For ECM, images were acquired as two by four tiles of 512x512 pixels with 10% overlap, 24 z-slices 2μm apart every 30 minutes, and the tiles fused in Zen Blue. For histoblast proliferation and FUCCI markers, images were acquired as two tiles of 1024 x 1024 pixels with 10% overlap every 5 minutes.

Laser ablations

Pupae were dissected and mounted as above. All movies were acquired on a Zeiss LSM 780 confocal microscope with a Coherent Chameleon NIR tunable laser, at 25°C. For single junction ablations, an alpha Plan-Apochromat 63x/1.46 Oil Korr M27 objective was used to acquire a single tile of 512 x 512 pixels with 5x zoom every 500ms for 31 seconds. Junctions were ablated with a wavelength of 780nm at 35% power with a 4 x 25 pixel ROI positioned across the junction with the ablation taking 0.254ms. The vertex location was identified manually and the recoil velocity calculated using the methodology outlined in Liang et al.[72]

For annular ablations, a Plan-Apochromat 40x/1.3 oil DIC M27 objective was used to acquire a single 512 x 512 pixel tile with 5 z-slices 1μm apart every 5s for 5 – 10 minutes. For generating ROIs, the freehand ROI tool was used to draw between two circles/ellipses. For histoblasts, the ROI was a circular annulus with an inner diameter of 45.54μm (110 pixels) and an outer diameter of 57.96μm (140 pixels), and the multi-photon was set at 780nm between 25 – 35% power, and the ablation was of a single z-plane at the apical surface and lasted 9.3ms. For LECs, an elliptical annulus ROI with inner diameter dimensions of 28.98μm (70 pixels) by 57.96μm (155 pixels) and outer dimensions of 38.916μm (94 pixels) by 57.96μm (185 pixels), and the multi-photon was set at 780nm at 40% power, and the ablation was of a single z-plane and lasted 8.3ms. To analyze the various parameters, we followed the methodology outlined in Bonnet et al.[73] Briefly, cells within the inner disc were segmented using Skeletor and the inner disc axes lengths measured. To obtain strain, a bounding ellipse was fitted to calculate the long and short axis and then the ellipse matrix was multiplied by a rotation matrix and transpose rotation matrix, to calculate the deformation along the DV (y) and AP (x) axes; the shear component was close to zero. Strain was then calculated by taking the natural log of the non-ablated axis length (l) divided by the relaxed axis length (Lr); ($\varepsilon = \ln(L/Lr)$. To calculate disc recoil velocity, we measured the change in axis length between successive frames. To calculate relaxation time, we took the inverse of the gradient from a straight line model fitted to recoil velocity as a function of axis length.

Quantification of cell number

For pupal staging, white pupae (0 hAPF) were collected with a paintbrush. After selection, animals were transferred to fresh vials and allowed to develop at 25°C until time of dissection.

Before dissection pupae were gently cleaned with 1xPBS and placed in double sided tape. First, both the anterior and posterior ends were cut with scissors. Then, pupae were bisected laterally along the antero-posterior axis. Animals were then transferred to sterilized 1x PBS and the internal organs were cleaned from the epidermis by flushing with 1x PBS. Using forceps, the epidermis was detached from the pupal case and transferred to an eppendorf. Fixation was performed for 20 minutes in 4% formaldehyde. After fixation, the epidermis was rinsed three times in 1xPBS (3x5 minutes). Finally, the tissue was equilibrated in Vectashield (with DAPI) overnight and mounted on coverslips. For the first hours of pupa development, 0 hAPF animals were selected and imaged every 5 minutes for the following 4 hours to allow for manual scoring the time of first and second divisions.

For quantification of cell number at 16 hAPF in HA, TIMP and MMP overexpressing conditions, pupae were reared as mentioned previously and imaged at 16 hAPF as mentioned previously.

Fluorescence quantification

To quantify apical *Sqh::GFP* intensity throughout development, movies were generated of *E-cad::mKate2; Sqh::GFP* as previously mentioned with a five hour frame rate to minimize photo-bleaching. Using the E-cad channel, the apical surface was projected as mentioned previously, for both E-cad and Sqh channels. The E-cad was then segmented using the AI algorithm and manually corrected. The intensity of Sqh was calculated for each junction, as well as the length and angle of junction relative to the DV axis. To calculate the median and interquartile range of Myo II intensity junctions were binned every 5°.

To quantify ECM dynamics, movies were acquired as mentioned previously. For analyzing pre-pupal dynamics an ROI of the final abdominal position within the field of view was generated and the mean intensity of ECM components measured over-time. Intensity data was normalized to the value at 13 hAPF for each movie which is the first time point after head eversion where all movement has ceased. For analyzing pupal dynamics an ROI was generated for the ECM under histoblasts and LECs, and the intensity for each cell population calculated over-time. Values were subtracted and normalized to the mean value of the five frames with the lowest mean intensity under the LECs.

To quantify FUCCI marker intensity over-time, movies were acquired as previously mentioned, and the total intensity for both the red and green channels were measured over time. These values where normalized to the total intensity in the first frame of the movie. To measure cell-cycle phase with the FUCCI marker, nuclei of cells were manually selected using both channels. The intensity value for each nuclei was then normalized to the mean intensity of all nuclei intensity for each channel. The ratio of RFP/GFP was then taken for each cell. Cell-cycle phase was attributed based on a threshold as indicated in Figures S1B and S1C.

QUANTIFICATION AND STATISTICAL ANALYSIS

All statistical tests were performed using R, Graphpad Prism or Wolfram Mathematica. Statistical tests used and number of repeats are indicated in the figure legends or in the text.

Current Biology, Volume *32*

Supplemental Information

ECM degradation in the *Drosophila* abdominal epidermis initiates tissue growth that ceases with rapid cell-cycle exit

John Robert Davis, Anna P. Ainslie, John J. Williamson, Ana Ferreira, Alejandro Torres-Sánchez, Andreas Hoppe, Federica Mangione, Matthew B. Smith, Enrique Martin-Blanco, Guillaume Salbreux, and Nicolas Tapon

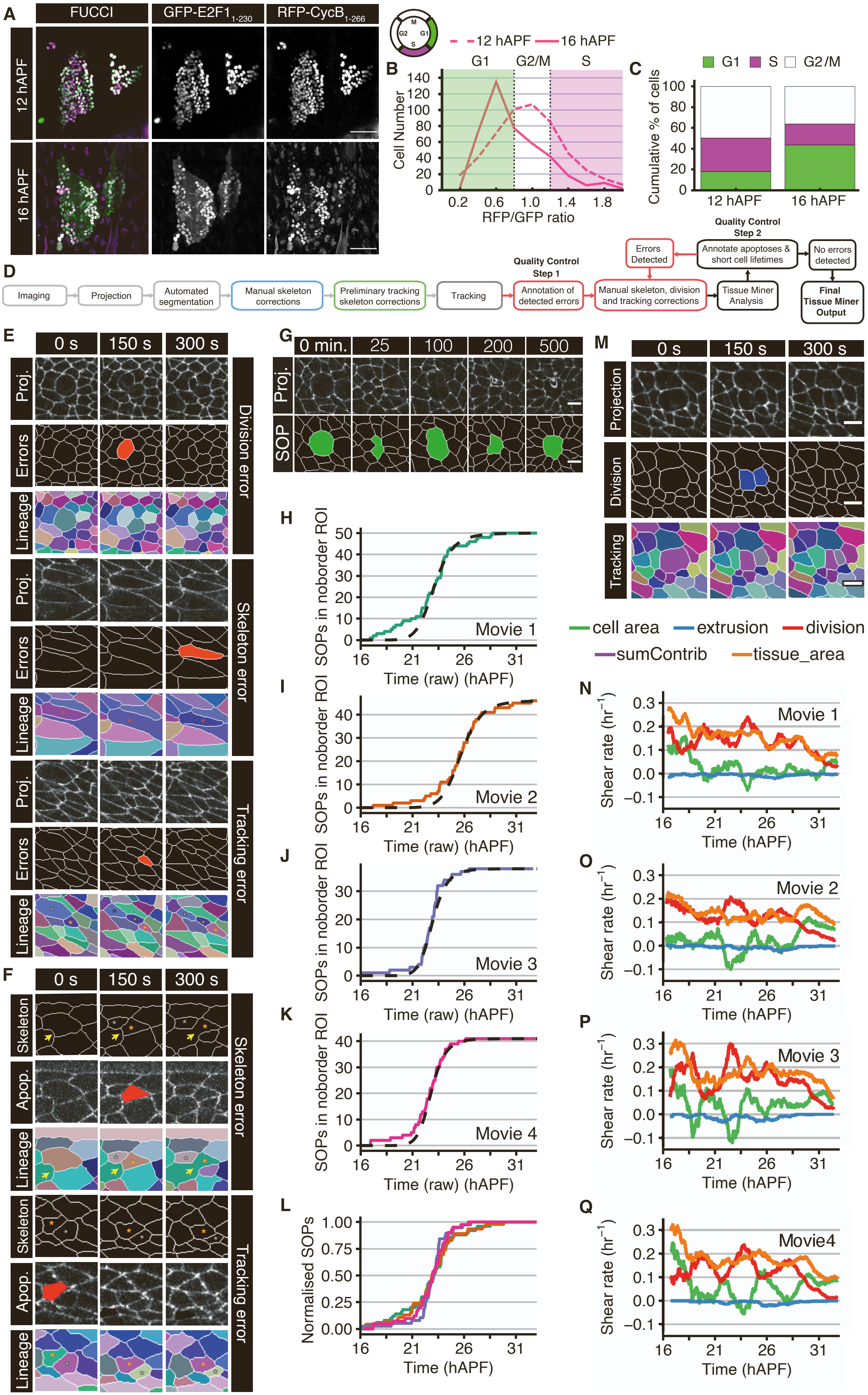

A
FUCCI
GFP-E2F1 1-230
RFP-CycB 1-266
12 hAPF
16 hAPF
B
12 hAPF
16 hAPF
G1
G2/M
S
Cell Number
RFP/GFP ratio
C
G1
S
G2/M
Cumulative % of cells
12 hAPF
16 hAPF
D
Imaging
Projection
Automated segmentation
Manual skeleton corrections
Preliminary tracking skeleton corrections
Tracking
Quality Control Step 1
Annotation of detected errors
Manual skeleton, division and tracking corrections
Errors Detected
Quality Control Step 2
Annotate apoptoses & short cell lifetimes
Tissue Miner Analysis
No errors detected
Final Tissue Miner Output
E
0 s
150 s
300 s
Proj.
Errors
Lineage
Division error
Skeleton error
Tracking error
F
Skeleton
Apop.
G
0 min.
25
100
200
500
SOP
H
SOPs in noborder ROI
Time (raw) (hAPF)
Movie 1
I
Movie 2
J
Movie 3
K
Movie 4
L
Normalised SOPs
Time (hAPF)
M
Projection
Division
Tracking
cell area
extrusion
division
sumContrib
tissue_area
N
Shear rate (hr−1)
Movie 1
O
Movie 2
P
Movie 3
Q
Movie4

Figure S1. Segmentation, tracking and temporal alignment of histoblast movies, Related to Figure 1, Video S1, S2, and Method S1.

(A) Example confocal images of wild-type and pupa expressing the FUCCI cell-cycle reporter markers *GFP-E2F1* (middle) and *RFP-CycB* (right) at the timepoints indicated. Schematic on far-right shows expected combined color for cells at the different stages of the cell cycle. Scale bars = 50μm.

(B) Quantification of normalized RFP/GFP signal ratio per cell at 12 hAPF and 16 hAPF pupae. N = 2 at each timepoint.

(C) Quantification of the percentage of cells at each cell-cycle phase based on normalized RFP/GFP signal ratio with the following thresholds G1 < 0.8 < G2/M < 1.4 < S-phase and highlighted in **B**.

(D) Overview of the pipeline including imaging, skeletonization, correction steps, tracking, quality control steps, and final output for analysis (see **STAR Methods** for details).

(E) Examples of error types detected by Tracker. Top, stills from a movie of a live pupal abdomen expressing *E-cad::GFP* after undergoing contour projection. Middle, error output generated by Tracker, with errors labelled in red. Bottom, lineage output from Tracker with each cell labelled with a unique color. Red and black stars indicate the location of the same cell and daughter cells of the same lineage between frames. Division error: red cell occurs because division has not been accurately detected by Tracker, thus the new cell is detected as an error. Skeleton Error: red cell occurs because of missing junction. When junction returns at 300 s, the cell has a new I.D., which is labelled as an error. Tracking error: red cell occurs due to rapid cell migration and cell division. New daughter cell from division is mistakenly labelled as a pre-existing cell at 150 s (black star, dark purple cell), and pre-existing cell is mistakenly labelled with a new cell I.D. at 150 s (red star, pink cell), which is labelled as an error.

(F) Examples of supposed cell losses (loss of cell I.D) detected by Tissue Miner which are skeleton and tracking errors. Top, skeleton analyzed by Tissue Miner. Middle, stills from a movie of a pupa expressing *E-cad::GFP* after undergoing contour projection, with the lost cell labelled in red by Tissue Miner. Bottom, lineage output from Tracker with each cell labelled with a unique color. Yellow arrows point towards the missing junction. Red and black stars indicate the same cell between frames. Skeleton Error: Missing junction labelled with yellow arrow at 300 s means that the final frame for the brown cell is at 150 s, thus it is labelled as a cell loss event at this time. Tracking Error: Cells are migrating rapidly and Tracker mislabels the green cell as the purple cell at 150 s. The green cell is labelled as an imminent apoptosis at the final frame where it is visible at 0 s.

(G) Top: Stills from a movie of a pupa expressing *E-cad::GFP*, following a sensory organ precursor cell (SOP) undergoing differentiation. Bottom: Skeletonization of SOP as a single cell (green-filled) from the moment of first asymmetric division to simplify the segmentation and tracking process. Scale bar = 5 μm.

(H-L) Time-alignment method for multiple wild-type movies. **(L)** Normalized count of the appearance of sensory organ precursors (SOPs) in the noborder ROI over time. Each movie is time-aligned relative to movie 1 (whose first frame is set to be at 16 hAPF), by an offset determined by fitting Hill functions to the individual movie data shown in **(H-K)**.

(M) Tracking and cell-division masks for input into the Tissue Miner software. Top row: example movie frames showing a cell division. Middle row: cell division mask of the same frames. In the first frame after the division, the newly-produced daughter cells are marked blue. Bottom row: tracking mask, in which each cell is marked with a unique color that persists over time. To match the convention Tissue Miner expects, one daughter cell retains the color of the mother, but will be parsed as a new cell by Tissue Miner. Scale bar = 5 μm.

(N-Q) Contributions to area expansion rate of the noborder ROI from cell area relative rate of change, cell division rate, cell extrusion rate. The sum of these 3 contributions matches the directly measured area expansion rate. Integration of the shear rate leads to the cumulative contributions, e.g. as for movie 1 in **Figure 1F**.

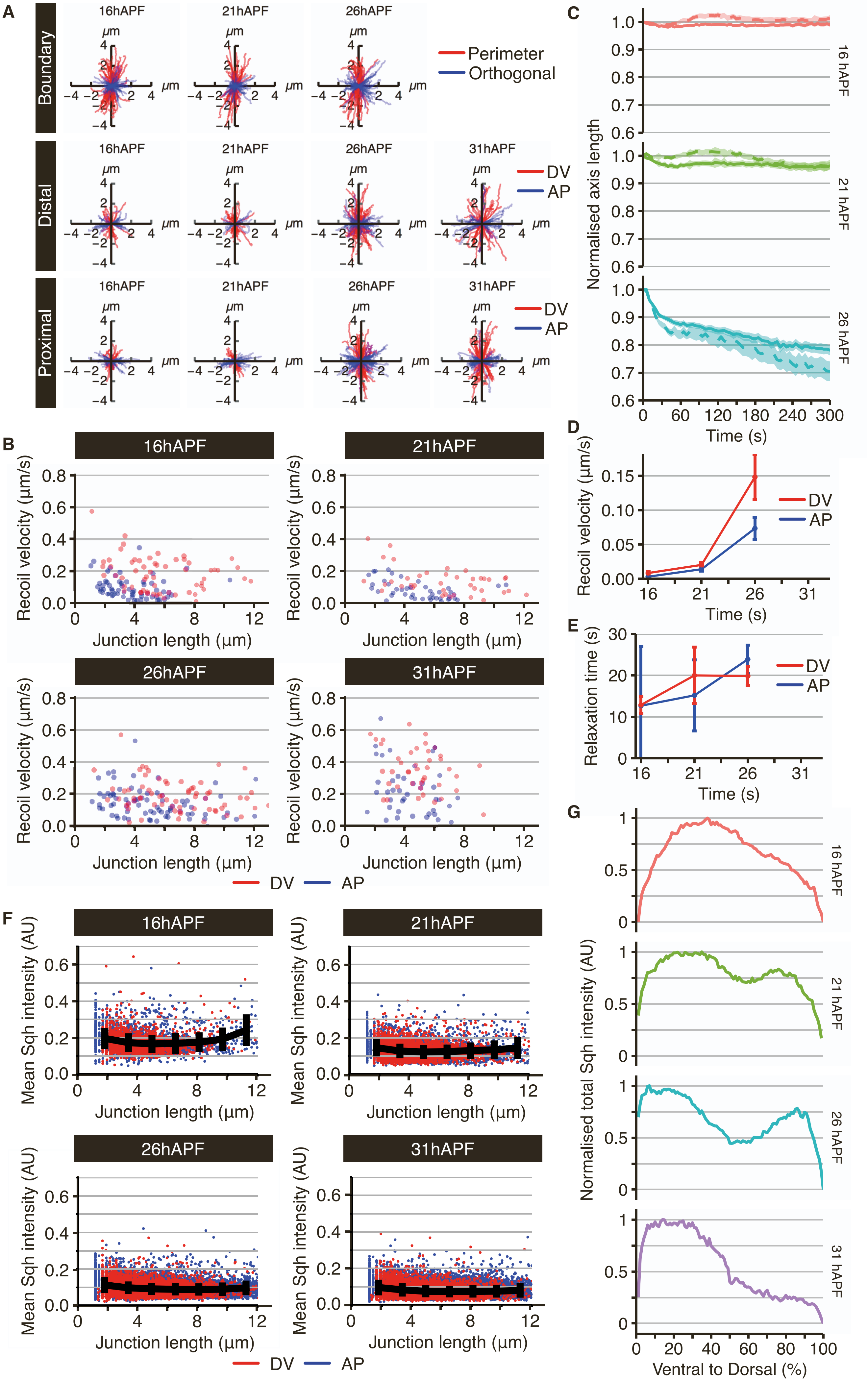

A
Boundary
Distal
Proximal
16hAPF
21hAPF
26hAPF
31hAPF
μm
Perimeter
Orthogonal
DV
AP
B
Recoil velocity (μm/s)
Junction length (μm)
DV
AP
C
Normalised axis length
16 hAPF
21 hAPF
26 hAPF
Time (s)
D
Recoil velocity (μm/s)
Time (s)
DV
AP
E
Relaxation time (s)
Time (s)
DV
AP
F
Mean Sqh intensity (AU)
Junction length (μm)
DV
AP
G
Normalised total Sqh intensity (AU)
16 hAPF
21 hAPF
26 hAPF
31 hAPF
Ventral to Dorsal (%)

Figure S2. Histoblast junction strain increases through development and is independent of junction length or Myo II intensity, Related to Figure 2, Video S3 and S4.

(A) Trajectories of vertices after single junction ablations, relative to their initial positions, in the different populations of histoblasts (see **Figure 2B**) throughout development. Note that for boundary cells, the trajectories of vertices shared with LECs are on the left and experience reduced displacement compared to the vertex within the nest.

(B) Quantification of junction recoil velocity as a function of junction length throughout development reveals no clear correlation.

(C) Normalized length of the short (oriented along the AP axis, dashed) and long (oriented along the DV axis, solid) axes in LECs after annular ablation.

(D, E) Quantification of recoil velocity **(D)** and relaxation time **(E)** along the short (oriented along the AP axis) and long (oriented along the DV axis) axes in LECs after annular ablations.

(F) Sqh::GFP (Myo II) intensity as a function of junction length. Dots are individual data points (red: DV oriented junctions, blue: AP oriented junctions), black line and error bars: binned mean and SD.

(G) Normalized Myo II intensity along the DV axis, across the anterior histoblast nest. Note the dip in intensity at the dorsal side.

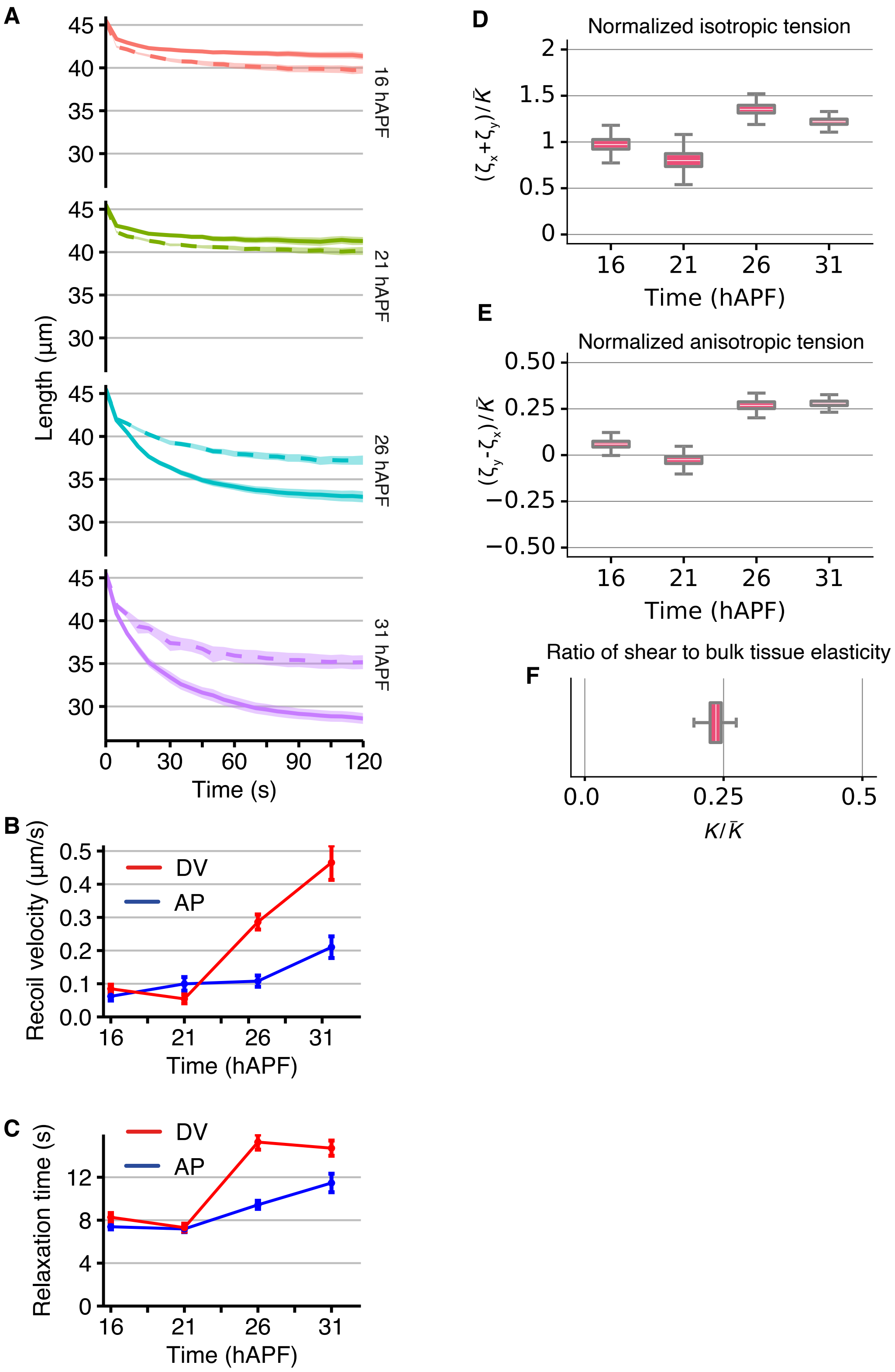

A
B
C
D
E
F
Length (µm)
16 hAPF
21 hAPF
26 hAPF
31 hAPF
Time (s)
Recoil velocity (µm/s)
DV
AP
Time (hAPF)
Relaxation time (s)
Normalized isotropic tension
$(\zeta_x+\zeta_y)/\bar{K}$
Normalized anisotropic tension
$(\zeta_y-\zeta_x)/\bar{K}$
Ratio of shear to bulk tissue elasticity
$K/\bar{K}$

Figure S3. Analysis of annular ablation experiments through histoblast development, Related to Figure 3, and Video S3.

(A) Length of AP (dashed) and DV (solid) axes in excised discs after annular ablation.

(B, C) Quantification of recoil velocity **(B)** and relaxation time **(C)** along the DV and AP axes of excised histoblasts after annular ablation, throughout development.

(D, E) Normalized isotropic (sum of AP and DV tensions, ζ_x+ζ_y, **D**) and anisotropic (difference of DV and AP tensions, ζ_y-ζ_x, **E**) tissue tensions.

(F) Ratio of tissue shear to bulk elastic moduli, obtained from a fit describing the tissue as an elastic material under tension, to measured excised discs deformation following laser ablation (see **Method S1**).

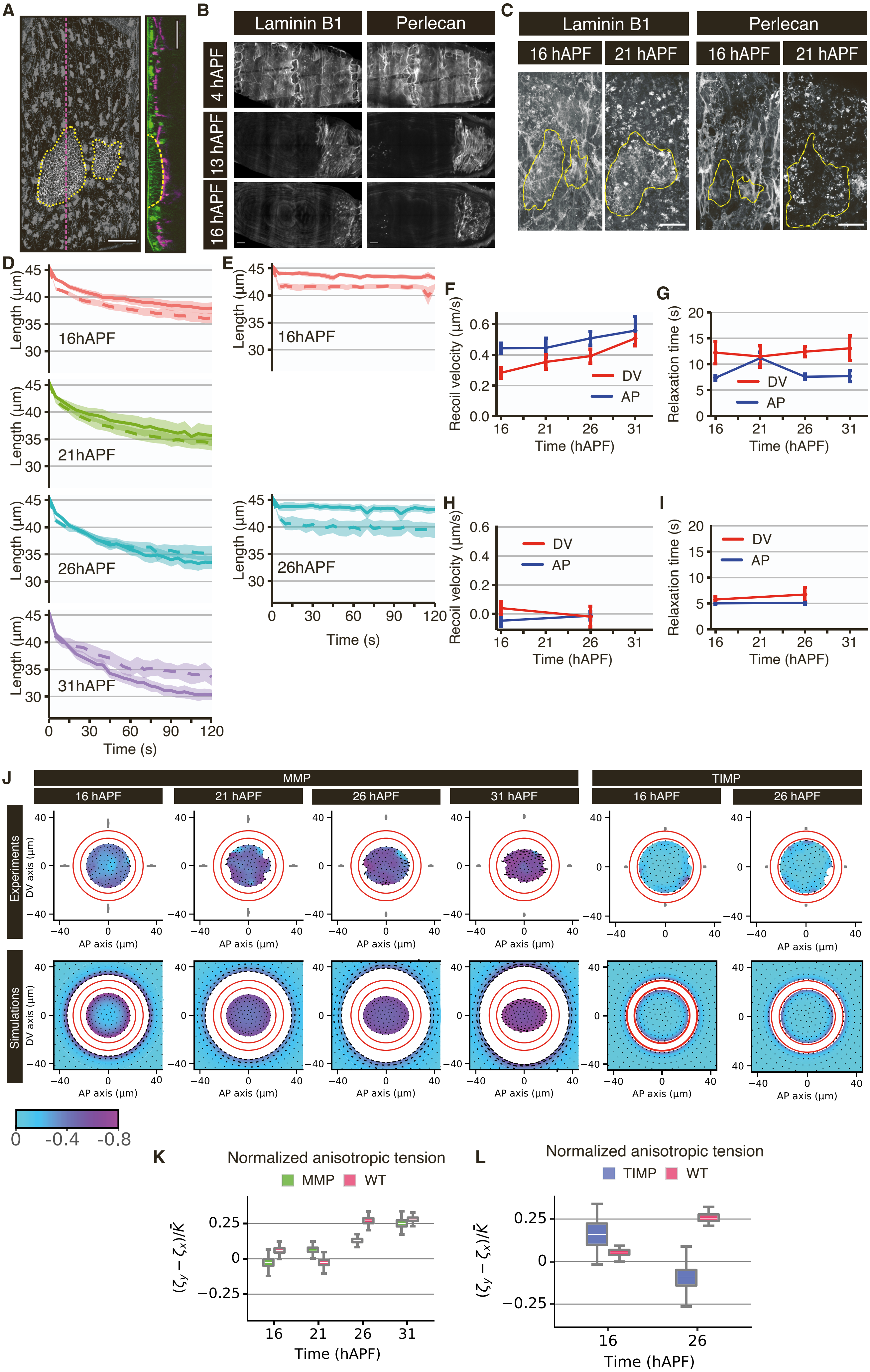

A
B
Laminin B1
Perlecan
4 hAPF
13 hAPF
16 hAPF
C
Laminin B1
Perlecan
16 hAPF
21 hAPF
16 hAPF
21 hAPF
D
Length (µm)
16hAPF
21hAPF
26hAPF
31hAPF
Time (s)
E
Length (µm)
16hAPF
26hAPF
Time (s)
F
Recoil velocity (µm/s)
DV
AP
Time (hAPF)
G
Relaxation time (s)
DV
AP
Time (hAPF)
H
Recoil velocity (µm/s)
DV
AP
Time (hAPF)
I
Relaxation time (s)
DV
AP
Time (hAPF)
J
MMP
TIMP
16 hAPF
21 hAPF
26 hAPF
31 hAPF
16 hAPF
26 hAPF
Experiments
Simulations
DV axis (µm)
AP axis (µm)
0
-0.4
-0.8
K
Normalized anisotropic tension
MMP
WT
$(\zeta_y - \zeta_x)/\bar{K}$
Time (hAPF)
L
Normalized anisotropic tension
TIMP
WT
$(\zeta_y - \zeta_x)/\bar{K}$
Time (hAPF)

Figure S4. Tissue mechanics are affected in pupae expressing MMP1 or TIMP, Related to Figure 4, Video S3 and S5.

(A) Snapshot of a pupa expressing *E-cad::Tom* and *Vkg::GFP* (Collagen IV) at 16 hAPF. Yellow dotted lines outline histoblasts. Magenta dotted line indicates position of orthogonal view. Left panel: Maximum projection of *E-cad::Tom*. Right panel: orthogonal projection. Scale bars = 50 μm.

(B) Example confocal images for Perlecan and Laminin B1 basal ECM components during the pre-pupal to early pupal stages. The head everts at 12 hAPF, pushing the abdomen posteriorly and this process is complete by 13 hAPF.

(C) Example confocal images for Perlecan and Laminin B1 basal ECM components during pupal stages. Histoblast nests are outlined in yellow. Scale bars = 50μm.

(D) Length of AP (dashed) and DV (solid) axes in excised cells from MMP1 expressing pupae under the control of *32B-GAL4* after annular ablation.

(E) Length of AP (dashed) and DV (solid) axes in excised cells from TIMP expressing pupae under the control *32B-GAL4* after annular ablation.

(F-I) Quantification of recoil velocity **(F, H)** and relaxation time **(G, I)** of excised histoblasts after laser ablation, along the DV and AP axes throughout development, in MMP1 **(F, G)** and TIMP **(H, I)** expressing pupae.

(J) Experiment (top) and simulation (bottom) deformation plots for excised histoblast discs, at 4 different time points, in pupae expressing MMP1 (left panels) or TIMP (right panels) under the control of *32B-GAL4*. Representation is as in **Figure 3E**. From left to right, n=11, 7, 7, 6, 7, 5 experiments.

(K, L) Normalized anisotropic tension (difference of DV and AP tensions, ζ_y-ζ_x), in pupae expressing MMP1 (green, **K**) and TIMP (blue, **L**), compared to parameters for WT (red, same as **Figure S3E**).

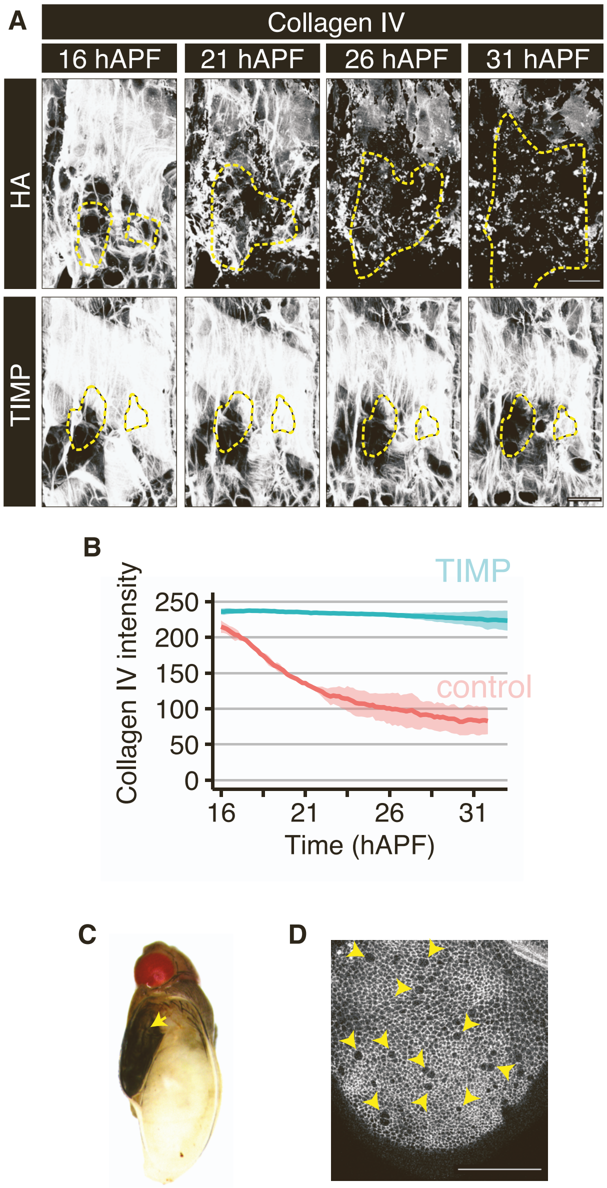
A
Collagen IV
16 hAPF
21 hAPF
26 hAPF
31 hAPF
HA
TIMP
B
TIMP
control
Collagen IV intensity
250
200
150
100
50
0
16
21
26
31
Time (hAPF)
C
D

Figure S5. Blocking ECM degradation in histoblasts with TIMP overexpression does not affect wing disk development, Related to Figure 5 and Video S6.

(A) Snapshots of pupae expressing *Vkg::GFP* (Collagen IV), and overexpressing HA (control) or TIMP under the control of *32B-GAL4* throughout development. Yellow dotted line outlines the histoblasts as seen with *E-cad::Tom* (not shown). Scale bar = 50 μm.

(B) Quantification of the mean intensity of Collagen IV from movies of pupae expressing HA (control) or TIMP under the control of *32B-GAL4* (Error bars: SD, HA n=2, TIMP n=2).

(C) Pharate pupa overexpressing TIMP under the control of *32B-GAL4*. Yellow arrows indicate the normal development of the wing.

(D) Still of wing disc from a movie of a pupa expressing *E-cad::GFP*, and overexpressing TIMP under the control of *32B-GAL4* at the same developmental time as histoblast expansion is inhibited. Note that cell divisions are still occurring (yellow arrowheads). Scale bar = 50 μm.

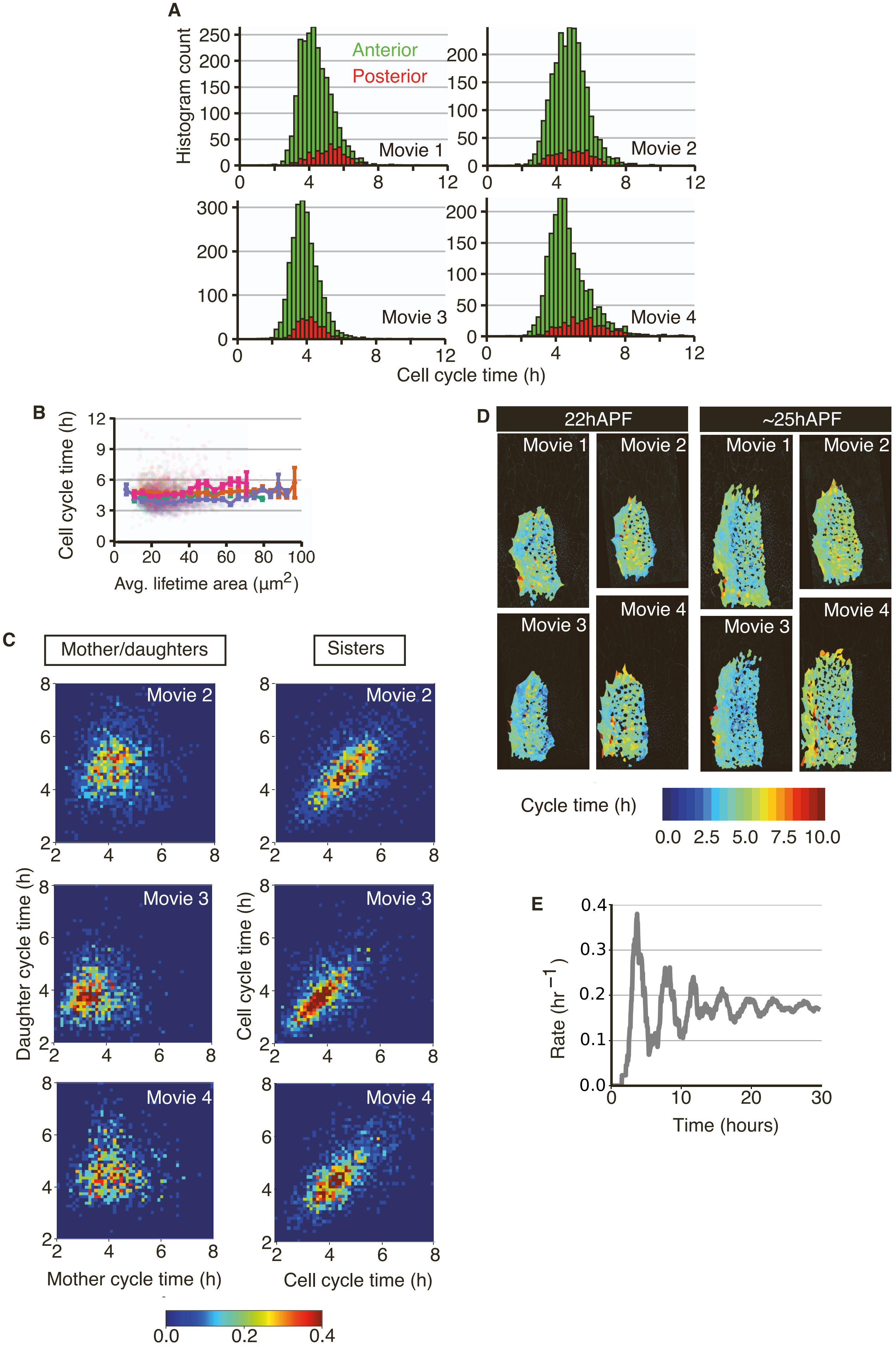
A
Histogram count
Anterior
Posterior
Movie 1
Movie 2
Movie 3
Movie 4
Cell cycle time (h)
B
Cell cycle time (h)
Avg. lifetime area (μm2)
C
Mother/daughters
Sisters
Movie 2
Movie 3
Movie 4
Daughter cycle time (h)
Cell cycle time (h)
Mother cycle time (h)
Cell cycle time (h)
0.0
0.2
0.4
D
22hAPF
~25hAPF
Movie 1
Movie 2
Movie 3
Movie 4
Cycle time (h)
0.0 2.5 5.0 7.5 10.0
E
Rate (hr −1)
Time (hours)

Figure S6. Cell cycle time analyses during abdominal development, Related to Figure 6 and Movie 7.

(A) Histograms of cell cycle times (defined only for cells that are observed appearing and disappearing via division events) in the anterior and posterior nests in movies 1-4.

(B) Cell cycle time as a function of the cell apical area, averaged over the lifetime of the cell.

Solid lines: binned data. Error bars: SEM for the bin. Transparent dots: individual data points. Color code for different movies is as in **Figure 6**.

(C) Probability density of pairs of cell cycle times, where pairs are taken between mother-daughters and sisters. Experimental data from movies 2-4 (see **Figure 6E-F** for movie 1). Cells are taken from the visible anterior nest. High densities of pair along the diagonal indicate high levels of correlation in the pair of cell cycle times.

(D) Snapshots from each wild-type movie with histoblast cells colored according to their cycle time at two intermediate times 22 hAPF and ~25 hAPF. Uncolored (black) cells are SOPs, arrested cells, cells which cannot be tracked from birth to mitosis, or cells with a cycle time larger than 10 hours.

(E) Cell division rate for one realization of a simulation of a cell population growing during 30 h. Cell cycle times are taken from a normal probability distribution with mean 4 h and coefficient of variation 0.2, roughly similar to the experimental distribution in **(A)** (see **Method S1** for details). At t=0 h the simulation starts with 100 cells, at the beginning of their cell cycle. The initial cell synchrony results in damped oscillations in the cell division rate. A moving average is applied as in **Figure 6A**.

A

Anterior nest growth trajectory key quantities	Mean ± SD
(*i*) Starting cell number at 0hAPF	18 ± 2
(*ii*) Cell number after cleavage divisions at 12hAPF	180 ± 20
(*iii*) Estimated cell number end of development 40hAPF	2900 ± 200
(*iv*) Average cleavage divisions per cell (up to 12hAPF)	3.3 ± 0.2
(*v*) Average expansion divisions per cell (14hAPF to end)	4.0 ± 0.2
(*vi*) Average cycle time cleavage - early direct measurements (hours)	2.7 ± 0.3
(*vii*) Average cycle time cleavage - estimate over 0-12hAPF (hours)	3.6 ± 0.2
(*viii*) Average cycle time expansion (hours)	4.5 ± 0.6

B

Parameter	Details on parameters for tissue growth simulations	Base Case Value
N_0	Anterior nests cells at 0hAPF	18
$[\text{ttd}]_0$	Mean time to first division at 0hAPF	1.58h
μ_0	Mean cell cycle time up to 3.3hAPF	2.67h
CV_0	Cell cycle time CV up to 3.3hAPF	0.24
μ_1	Mean cell cycle time between 3.3hAPF and 12.5hAPF	4.6h
CV_1	Cell cycle time CV between 3.3 and 12.5hAPF	0.32
$t_{pause\text{-}on}$	Pause in cell division starts	12.5hAPF
t_{main}	Pause in cell division ends	14.7hAPF
η_{sub}	Subset at t_{main} to be noborder ROI	95
$\mu_2(t)$	Mean cell cycle time after 14.7hAPF	Tracks expt. data
CV_2	Cell cycle time CV after 14.7hAPF	0.2
ρ_s	Correlation coefficient of sister cycle times	0.55
s_p	Hill function switch times for arrested cell creation	25.7hAPF
s_a	Hill function switch times for arrested cell creation	24.7hAPF
$h_p = h_a$	Hill function coefficients for arrested cell creation	34
p_{SOP}	Probability that a new non-arrested cell is an SOP during SOP generation period	0.09
$t_{SOP\text{-}on}$	SOP generation period start time	20.6hAPF
$t_{SOP\text{-}off}$	SOP generation period stop time	24hAPF

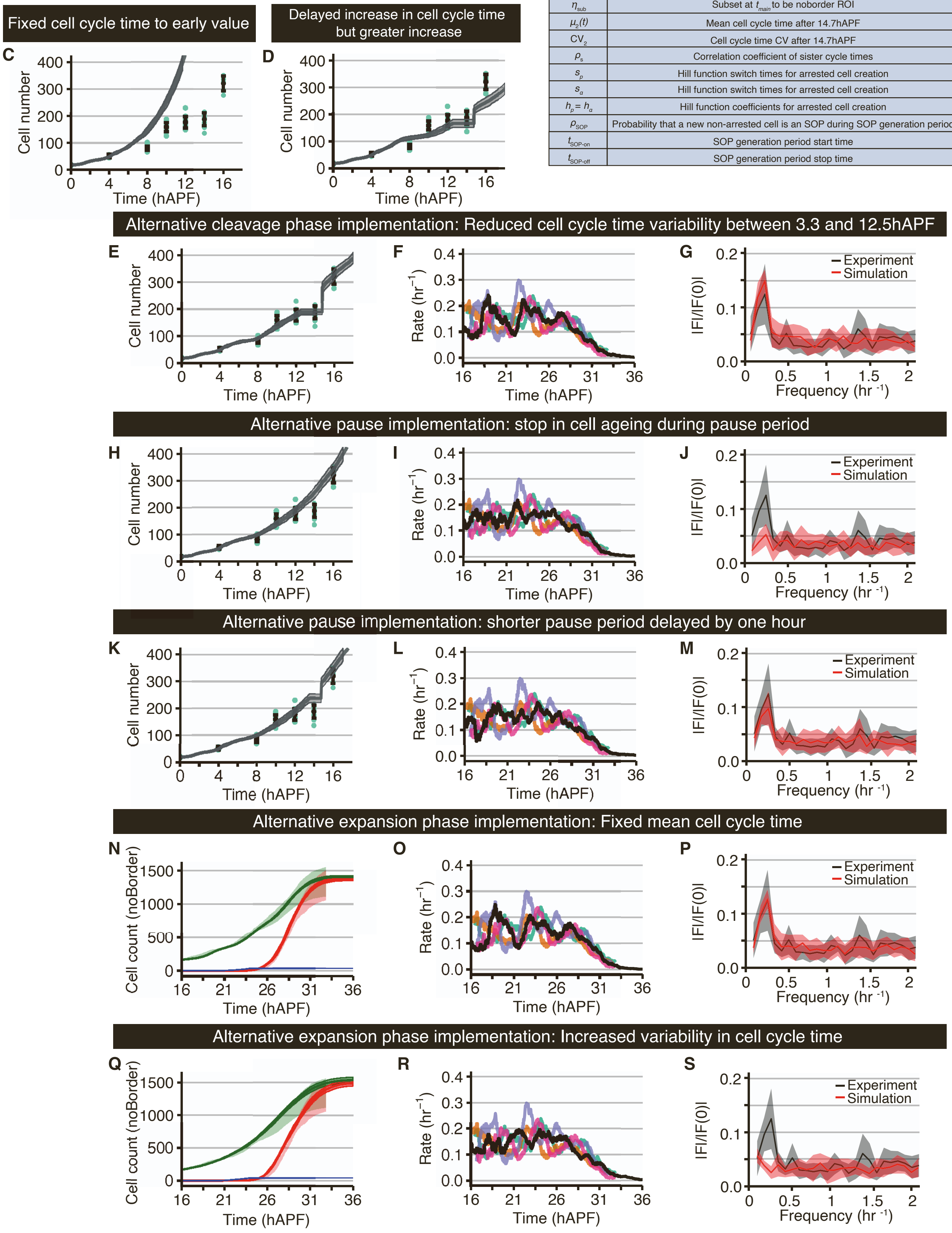

Figure S7. Simulations of histoblast proliferation kinetics, Related to Figure 7 and Method S1.

(A) Anterior nest growth trajectory key quantities. (***i***) Anterior only cell counts from **Figure 7C** (n = 15). (***ii***) Anterior only cell counts from **Figure 7C** (n = 13). (***iii***) Estimate from simulation. The base case simulation reported in **Figure 7** is parameterised to the noborder ROI. Assuming this is a representative subset of the entire anterior nest, the simulation yields an extrapolation for the final cell number in the anterior nest. The reported standard deviation is from n = 10 simulation runs, whose variability is significantly smaller than the experimental variability (see **Figure 7E**). (***iv***) Comparing measured cell number at 12 and 0 hAPF and using the formula $N(t) / N_0 = 2^{n_{div.}}$ where N is cell number and $n_{div.}$ the average number of divisions per cell yields this estimate. Compare **Figure 7D**. (***v***) Using the estimated final cell number from part (***iii***) and the measured cell number at 14 hAPF (prior to the rapid onset of expansion divisions) yields this estimate. (***vi***) Direct measurements available in the first few hAPF (see **Figure 7B**) yields this estimate. Three movies were used, each yielding 20-40 cycle time measurements to generate an average for that movie. The value and uncertainty reported here are the average and standard deviation of those per-movie averages. (***vii***) Using the formula $N(t) / N_0 = 2^{n_{div.}}$ as in part (*iv*), the mean divisions per cell $n_{div.} = t / \mu_{\text{cleavage}}$ where t is the time and μ_{cleavage} the mean cycle time of cleavage divisions. Evaluation at t = 12 hAPF yields the reported estimate. It is larger than for the direct measurement of part (*vi*). This may indicate that the average cycle time of cleavage divisions really becomes longer, or that some cells do not complete the mean number of cleavage divisions. Either is compatible with the apparent slowing down of cell number increase from 0-12 hAPF which can be seen in **Figure 7C**. (***viii***) The coloured data points in **Figure 7B** represent measurements of cycle time during the expansion phase from the four wild-type movies, in the noborder ROI. A simple average of these data points yields the reported value. Colour code represents stages of histoblast growth as highlighted in **Figure7A**.

(B) Table of parameters for the base case of the tissue growth simulation (**Figure 3E-H**). CV: coefficient of variation. Colour code represents stages of histoblast growth as highlighted in **Figure7A**.

(C) Number of anterior cells before 16 hAPF, experiment and simulations. Simulations are as in **Figure 7**, but the mean cycle time is set to be constant and equal to the value measured experimentally before 3 hAPF. The cell number increases much too quickly.

(D) Number of cells before 16 hAPF, experiment and simulations. Simulation results are as in **Figure 7**, but the mean cell cycle time increases to 6.6 h at 4.3 hAPF, instead of increasing to 4.6 h at 3.3 hAPF. This choice leads to a roughly correct number of cells produced around 14 hAPF, but agreement with the measured number of cells over time appears overall less good than in **Figure 7C**.

(E-G) Simulation result with an alternative implementation of the cleavage phase. **(E)** Number of cells in the histoblast before 16 hAPF, as in **Figure 7C**; **(F)** Division rate as a function of time after 16 hAPF, color scheme as in **Figure 7F** and moving average is applied as in **Figure 6A**. **(G)** Normalized absolute value of the Fourier transform of the division rate. Simulation results are as in Figure 7 but the coefficient

of variation of cell cycle time between 3.3 hAPF and 14.7 hAPF has been set to 0.2 instead of 0.32. This results in a peak in the Fourier transform of the division rate which is slightly too high.

(H-J) Simulation result with an alternative implementation of the pause period around 12 hAPF. **(H)** Number of cells in the histoblast before 16 hAPF, as in **Figure 7C**; **(I)** Division rate as a function of time after 16 hAPF, color scheme as in **Figure 7F** and moving average is applied as in **Figure 6A**. **(J)** Normalized absolute value of the Fourier transform of the division rate. Whereas in the base case where divisions were prevented but cell aging continued, here cell aging is paused from 12.2 hAPF and is restarted at 12.7 hours. In this implementation divisions plateau for a short period around 12 hAPF, but the cell number after the pause increases more gradually. The age-stopping pause does not lead to the sudden burst of simultaneous divisions shown in **Figure 7C** and therefore the "re-synchronization", due to setting a large number of cell ages to zero simultaneously, does not occur.

(K-M) Simulation result with an alternative implementation of the pause period around 12 hAPF. **(K)** Number of cells in the histoblast before 16 hAPF, as in **Figure 7C**; **(L)** Division rate as a function of time after 16 hAPF, color scheme as in **Figure 7F** and moving average is applied as in **Figure 6A**; **(M)** Normalized absolute value of the Fourier transform of the division rate. Simulations as in **Figure 7**, but results are obtained with a shorter pause time as the pause starting time is delayed from 12.5 hAPF to 13.5 hAPF; resulting in less pronounced oscillations.

(N-P) Simulation result with an alternative implementation of the expansion phase after 16hAPF. **(N)** Number of cells after 16 hAPF, as in **Figure 7E**; **(O)** Division rate as a function of time after 16 hAPF, color scheme as in **Figure 7F** and moving average is applied as in **Figure 6A**; **(P)** Normalized absolute value of the Fourier transform of the division rate. Simulation results are as in **Figure 7** but the mean cycle time in the main phase is constant at 4.42 h, a value chosen by calculating the average (weighted by number of cells) of the binned cycle time data from **Figure 7B**. There are only subtle differences from the base case **Figure 7F**, for example the second division rate peak is slightly lower.

(Q-S) Simulation result with an alternative implementation of the expansion phase after 16 hAPF. **(Q)** Number of cells after 16 hAPF, as in **Figure 7E**; **(R)** Division rate as a function of time after 16 hAPF, color scheme as in **Figure 7F** and moving average is applied as in **Figure 6A**; **(S)** Normalized absolute value of the Fourier transform of the division rate. Simulation results are as in **Figure 7** but the CV after 14.7 hAPF is doubled to 0.4. The peaks in the division rate become noticeably less sharp and more disordered compared to the base case **Figure 7F**, and this is also reflected in the Fourier transformed data.
